# In-hospital mortality, readmission, and prolonged length of stay risk prediction leveraging historical electronic patient records

**DOI:** 10.1093/jamiaopen/ooae074

**Published:** 2024-09-14

**Authors:** Rajeev Bopche, Lise Tuset Gustad, Jan Egil Afset, Birgitta Ehrnström, Jan Kristian Damås, Øystein Nytrø

**Affiliations:** Department of Computer Science, Norwegian University of Science and Technology, Trondheim, 7491, Norway; Faculty of Nursing and Health Sciences, Nord University, Levanger, 7600, Norway; Department of Medicine and Rehabilitation, Levanger Hospital, Nord-Trøndelag Hospital Trust, Levanger, 7601, Norway; Department of Medical Microbiology, St Olavs Hospital, Trondheim University Hospital, Trondheim, 7030, Norway; Department of Clinical and Molecular Medicine, Norwegian University of Science and Technology, Trondheim, 7491, Norway; Department of Clinical and Molecular Medicine, Norwegian University of Science and Technology, Trondheim, 7491, Norway; Department of Infectious Diseases, Clinic of Medicine, St Olavs Hospital, Trondheim, 7006, Norway; Clinic of Anaesthesia and Intensive Care, St Olavs Hospital, Trondheim University Hospital, Trondheim, 7006, Norway; Department of Clinical and Molecular Medicine, Norwegian University of Science and Technology, Trondheim, 7491, Norway; Department of Infectious Diseases, Clinic of Medicine, St Olavs Hospital, Trondheim, 7006, Norway; Department of Computer Science, Norwegian University of Science and Technology, Trondheim, 7491, Norway; Department of Computer Science, The Arctic University of Norway, Tromsø, 9037, Norway

**Keywords:** healthcare informatics, electronic patient records, tree-based models, predictive analytics, machine learning, eXplainable Artificial Intelligence, mortality, readmission, prolonged length of stay, medical history

## Abstract

**Objective:**

This study aimed to investigate the predictive capabilities of historical patient records to predict patient adverse outcomes such as mortality, readmission, and prolonged length of stay (PLOS).

**Methods:**

Leveraging a de-identified dataset from a tertiary care university hospital, we developed an eXplainable Artificial Intelligence (XAI) framework combining tree-based and traditional machine learning (ML) models with interpretations and statistical analysis of predictors of mortality, readmission, and PLOS.

**Results:**

Our framework demonstrated exceptional predictive performance with a notable area under the receiver operating characteristic (AUROC) of 0.9625 and an area under the precision-recall curve (AUPRC) of 0.8575 for 30-day mortality at discharge and an AUROC of 0.9545 and AUPRC of 0.8419 at admission. For the readmission and PLOS risk, the highest AUROC achieved were 0.8198 and 0.9797, respectively. The tree-based models consistently outperformed the traditional ML models in all 4 prediction tasks. The key predictors were age, derived temporal features, routine laboratory tests, and diagnostic and procedural codes.

**Conclusion:**

The study underscores the potential of leveraging medical history for enhanced hospital predictive analytics. We present an accurate and intuitive framework for early warning models that can be easily implemented in the current and developing digital health platforms to predict adverse outcomes accurately.

## Objectives

### Background and significance

Important healthcare indicators, such as 30-day mortality, 30-day readmissions, and prolonged length of stay (PLOS), are essential for managing patient care and allocating resources efficiently.^1–3^ Accurate forecasts of these indicators are pivotal for the early identification of high-risk patients, leading to timely medical actions and improved patient outcomes.^4^^,^^5^ Electronic health record (EHR) encompasses diverse information systems, ranging from single-department files to extensive, longitudinal patient data collections. Electronic patient record (EPR), a subset of EHR, compiles an individual's healthcare data accumulated over time within the primary healthcare facility responsible for a patient's comprehensive care and medical record-keeping. In this context, all the patient’s health interactions, treatments, and medical history are documented and maintained within this institution.^6^ In Norway, patients often have long and continuous histories within one health system or hospital’s records, allowing for retrospective medical history analysis for patients served by the health system. EPRs have been used for secondary applications to address disease progression modeling,^7^ patient trajectory modeling,^8^ disease inference,^9^ risk stratification, and survival prediction.^10^ These data-driven analyses are increasingly needed in various health services and research. However, data in an EPR may be sparse and require context-dependent interpretation, which may cause incompleteness and, to a lesser extent, inconsistency and inaccuracy.^11^

Previous work on leveraging longitudinal medical data by Chicco et al. showed that traditional machine learning (ML) models predicted the survival of patients diagnosed with sepsis using minimal clinical records of patients.^10^ Some studies have tackled the problems of representing medical data and codes. For example, Tran et al worked on building a low-dimensional representation of medical events using a modified restricted Boltzmann machine (RBM). After that, they trained a logistic regression classifier for suicide risk stratification.^12^ In comparison, Jia et al used patient similarity-based frameworks to group similar patient histories.^13^ Other works focused on visualizing the medical history or building patient disease trajectories.^14^ The study by Choi et al. presented disease-specific applications that treated the medical history as a sequence of events and then trained ML models to predict diagnostic outcomes in the next event.^15^

In works predicting adverse outcomes in hospitals, a study by Cai et al^16^ developed a non-disease-specific Bayesian network (BN) model to predict mortality, readmission, and length of stay (LOS) from EHRs. Utilizing data from 32 634 patients admitted via emergency department to a Sydney hospital between 2008 and 2011, the model achieved an average daily accuracy of 80% with an area under the receiver operating characteristic curve (AUROC) of 0.82 for mortality, which was the most predictable outcome. Tavakolian et al introduced an optimized hybrid deep model termed Genetic Algorithm-Optimized Convolutional Neural Network (GAOCNN) for predicting hospital readmission and LOS. Tested on 3 distinct healthcare datasets, this model achieved impressive prediction accuracies, reaching 97.2% for hospital readmission prediction in diabetic patients and 89.0%, 99.4%, and 94.1% for LOS prediction in diabetic, COVID-19, and intensive care unit (ICU) patients, respectively.^17^ Clark et al explored a multistate model for predicting mortality, LOS, and readmission for surgical patients using the American College of Surgeons National Surgical Quality Improvement Program data.^18^ Most models focused on recent patient data, and none of the studies utilized the predictors from the complete medical history of their patients, apart from demographics and information on co-morbidities. In our previous work, through feature engineering from historical medical records and employing an array of ML classifiers, we showcased the efficacy of the eXtreme Gradient Boosting (XGBoost) model in predicting 30-day mortality using EHR trajectory features.^19^

The domain of health informatics (HI) has experienced significant advancements with the integration of artificial intelligence (AI) techniques, especially in predictive analytics. Various AI algorithms have been applied in HI, ranging from traditional ML models to more complex deep learning (DL) architectures.^20^^,^^21^ Tree-based models have gained popularity due to their robustness in handling medical data.^22^ While AI models can produce accurate predictions, their “black box” nature has been a concern in the medical domain due to the critical nature of healthcare decisions.^23^ The integration of Explainable AI (XAI) in healthcare has gained significant attention, particularly its potential to enhance transparency and trust in predictive models using EHR. Several recent XAI architectures and techniques have been developed to address the unique challenges associated with EHR data. For example, Shapley Additive exPlanations (SHAP) values are derived from cooperative game theory and provide a unified measure of feature importance. SHAP is widely used in EHR applications because it offers consistent and interpretable explanations for model predictions across various ML models, including tree-based models and neural networks.^24^ Local Interpretable Model agnostic Explanations (LIME) explain individual predictions by locally approximating the model around a specific instance. It is model-agnostic and applies to various predictive models used in EHR analysis. LIME's local explanations help clinicians understand model decisions on a case-by-case basis.^25^ Integrated gradients is a technique designed for DL models that assign importance scores to input features by integrating gradients of the model’s output to the input. This method has been applied to EHR data to provide insights into the contributions of different clinical features.^26^ Attention mechanisms, particularly in recurrent neural networks (RNNs) and transformer models, have been employed to enhance interpretability in sequential EHR data processing. These mechanisms allow models to focus on relevant portions of the input data, providing intuitive explanations for predictions.^27^ ProtoDash is an interpretable prototype selection method that identifies representative examples from the dataset to explain predictions. It has been used in the context of EHR to provide exemplar-based explanations, helping clinicians relate model predictions to known clinical cases.^28^ Anchors is a high-precision model-agnostic explanation method that provides if-then rules (anchors) to describe the conditions under which a model makes specific predictions. This technique has been applied to EHR data to generate clear and actionable explanations for clinicians.^29^ The significance of XAI in healthcare is profound as it ensures both healthcare practitioners and patients can trust and understand AI-driven decisions.^30^ Though predictive analytics have shown promise, they also come with challenges.^31^ Data quality, missing values, and class imbalances are significant challenges in healthcare predictions.^32^^,^^33^ The ethical implications concerning patient data security and consent are also paramount.^34^ The recent introduction of XAI in HI offers transformative potential.^35^ It allows for complex data within EPRs to be analyzed in a way that can be understood by healthcare professionals, supporting informed clinical decisions.^36^^,^^37^ Despite the potential benefits, applying comprehensive data available within EPRs for predictive analysis has yet to be commonplace in hospital settings.^38^ Preliminary research suggests significant healthcare interactions can precede critical health events, such as cancer diagnoses.^39^ Few studies have leveraged the complete information from patients' medical history, and most only consider predictors from a specified time window.^16^^,^^17^ We theorize that historical EPRs contain patterns and early indicators of impending adverse hospital outcomes. To explore this, we conducted a retrospective analysis of the patient’s medical histories. We introduce the XAI-based Risk Analysis and Interpretation (XRAI) framework to predict the risk of hospital adverse events.

### Objectives and outcomes

The primary goal of this study is to investigate the predictive capabilities of historical EPRs maintained at hospitals to predict adverse outcomes such as mortality, readmission, and PLOS. Specifically, the study focuses on patients from a Norwegian hospital who were suspected of bloodstream infections (BSIs) at least once in the hospital, leveraging their comprehensive medical histories to develop predictive models. By doing so, the research aims to:

- Develop and validate an XAI framework that integrates ML models to predict 30-day mortality at discharge and admission, 30-day readmission following discharge, and PLOS at admission.
- Identify and analyze the critical predictors from historical EPRs, including demographics, laboratory test results, diagnostic and procedural codes, and derived temporal features.
- Demonstrate the application and performance of the XAI framework in a real-world hospital setting using a de-identified dataset from St Olavs University Hospital in Trondheim, Norway.
- Provide model explanations to ensure transparency and interpretability of the predictions for healthcare professionals.

The context of our predictions is to assess whether historical administrative data can predict hospital adverse events. The findings will inform strategies to enhance early warning systems, improve patient outcomes, and optimize resource allocation in healthcare settings.

## Methods

### Data

This study harnessed EPRs from St Olavs University Hospital, Trondheim, Norway, encompassing 35 591 patients with suspected BSIs identified via physician-initiated blood cultures between 2015 and 2020. In Norway, blood culture is a commonly ordered test in hospitalized patients when a severe infection is suspected, ensuring that the study population includes diverse patient demographics and clinical conditions. Detailed data are available for patients undergoing blood culture tests. Moreover, BSIs are a significant cause of morbidity and mortality in hospitalized patients. Identifying and predicting adverse outcomes in this patient population is clinically essential. The EPRs encompassed curated data from the inception of electronic records in 1999 until 2020, exclusively included hospital care episodes, ICU admission details, microbiology test results, laboratory test results, and patient demographics comprising of gender, date of birth, and date of death. Primary care and other specialist care episodes were not available in the dataset. Diagnoses and Procedures within these records were classified using the International Classification of Diseases, 10th Revision (ICD-10), facilitating standardized disease identification critical for the analytical models. We obtained the death dates from the Hemit Health IT data warehouse, which sourced the data from the National Patient Register (NPR), ensuring comprehensive coverage of deaths inside and outside the hospital. This study adhered to the transparent reporting of a multivariable prediction model for individual prognosis or diagnosis (TRIPOD).^40^ The EPRs were de-identified and accessed through a private cloud computing platform for ethical and privacy reasons.

### XRAI framework

**Figure 1.**
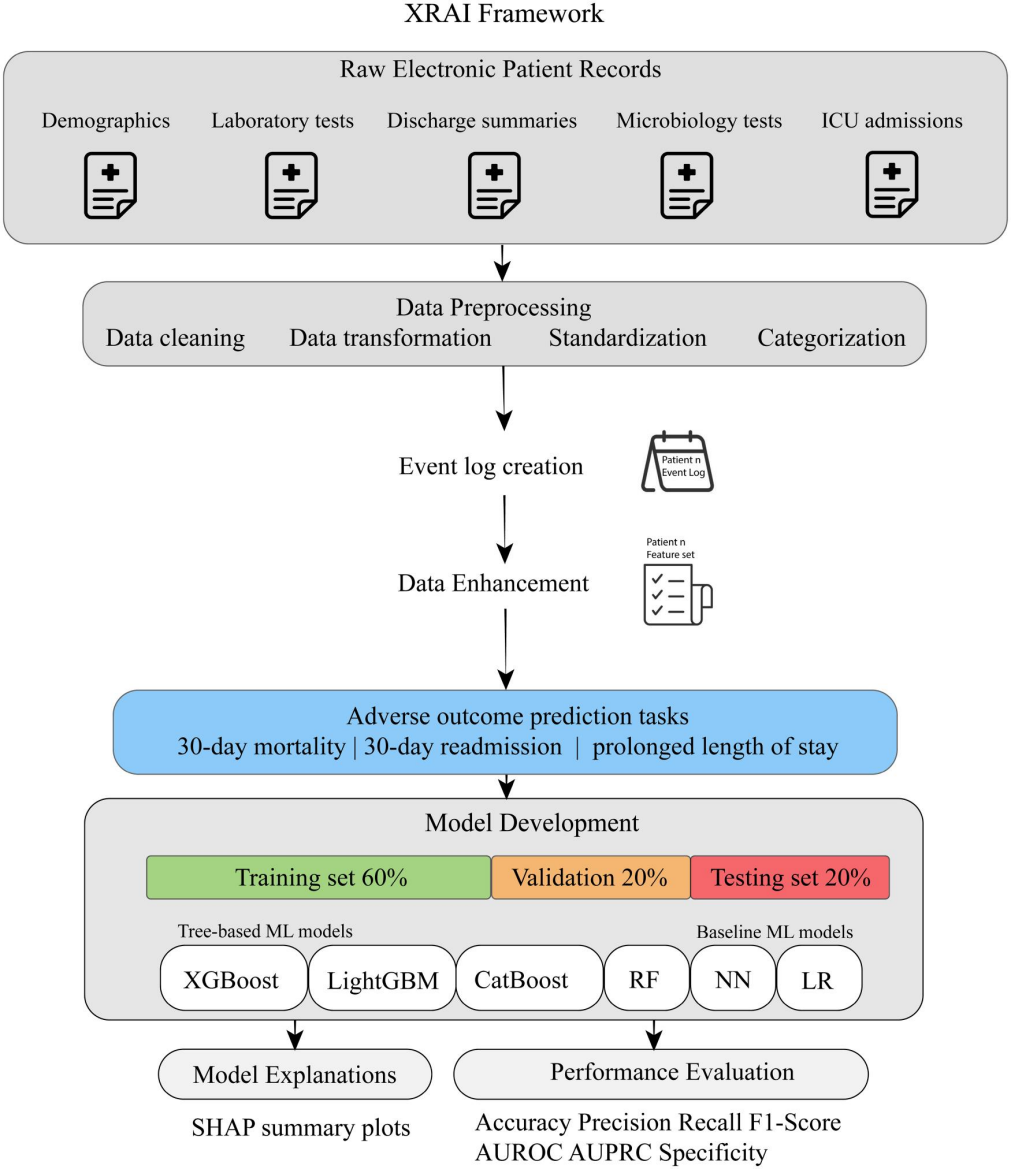
The architecture of the XRAI framework.

The XRAI (eXplainable Risk Analysis and Interpretation) framework uses historical EPR to predict adverse hospital outcomes, including mortality, readmission, and PLOS. This framework combines various data types and ML models, providing interpretable predictions to support clinical decision-making. The framework integrates diverse data from EPRs, including demographics, laboratory test results, microbiology tests, discharge summaries, and ICU admissions. Detailed patient statistics are provided in section “Patient characteristics.” These data are processed to create comprehensive patient profiles used for predictive modeling. Data preprocessing includes cleaning, standardizing, and categorizing information, ensuring the quality and consistency necessary for robust predictive analytics. Further details on the data preprocessing are given in Supplementary material S1. Four event logs were created for each patient ID, from episode discharge summaries, ICU admissions, laboratory tests, and microbiology test results, where hospital visits/admissions, ICU admissions, and particular laboratory and microbiology tests were considered separate and overlapping medical events, respectively. Event logs were used as a transitory setup for data augmentation and enrichment. More specifically, identifying index episodes, calculating temporal features, and visually analyzing patient histories. Figure 1 depicts the workflow followed by the XRAI framework.

Data enhancement within the XRAI framework involves extracting meaningful predictors from raw data. Key features include age, laboratory test results, microbiology test results, comorbidities, diagnostic and procedural codes, and hospital and ICU length of stay (LOS). Temporal features such as time to the most recent hospital admission and cumulative LOS from previous admissions are also included. These features are crucial for capturing the patient's health trajectory and risk factors. More details on the data enhancement step are given in Supplementary Information 1. The framework employs tree-based ML models, including XGBoost, LightGBM, and CatBoost, which have demonstrated superior performance on tabular data compared to DL techniques.^22^ Tree-based models provide high accuracy and interpretability on tabular data, making them suitable for clinical applications.^23^ Moreover, our previous work using the same dataset for predicting BSI showed that combining data enhancements and tree-based models performed better than complex RNNs and transformer models trained on sequences of medical events.^41^ We employed techniques to handle class imbalance, such as utilizing the class_weights and scale_pos_weight parameters in the tree-based ML models. See Supplementary material S1 for more details on the model development, evaluations, and interpretations. Model interpretability is a core component of the XRAI framework. Techniques such as SHAP are used to elucidate the contributions of individual features to model predictions.^24^^,^^42^^,^^43^ This transparency ensures that healthcare professionals can understand and trust the model outputs, facilitating informed clinical decisions.

### Predicted outcomes

Our study aimed to predict 4 critical outcomes using the XRAI framework: the risk of 30-day mortality at the time of discharge, the risk of 30-day mortality at the time of admission, the risk of readmission within 30 days following discharge, and the risk of PLOS (> 2 days) at the time of admission. An episode is defined as a unique discharge summary entry. The index episode was the last recorded hospital episode of all the patients for the mortality and PLOS prediction tasks and the second last recorded episode of all patients for the readmission prediction task. We observed the following outcomes among the 35 591 patients included in the analysis: For both 30-day mortality prediction tasks, Class 0 (no mortality within 30 days) comprised 28 173 patients (79.2%), while Class 1 (mortality within 30 days) included 7418 patients (20.8%). For 30-day readmission following discharge, Class 0 (no readmission) was represented by 29 655 patients (83.3%), and Class 1 (readmission) had 5936 patients (16.7%). For PLOS, Class 0 (no PLOS) consisted of 26 737 patients (75.1%), whereas Class 1 (PLOS) included 8854 patients (24.9%).

### Predictors

The predictors included age, sex, results of recent laboratory tests, results of microbiology tests, counts of comorbidities, counts of diagnostic and procedural codes, and current, recent, and total hospital and ICU LOS. The most common laboratory tests were bilirubin, C-reactive protein (CRP), creatinine, leukocytes, and thrombocytes. The counts of prior positive results of microbiology tests grouped by their collected sample type were calculated and used as indicators of previous history of infections. The total length of stay variable used in our analysis denotes the total time a patient has spent in the hospital, calculated as the cumulative sum of all previously recorded LOS before the index episode admission, excluding the LOS of the index episode itself. The prediction task modeling details are given in Supplementary material S1.

### Statistical analysis of predictors

To investigate the strength and significance of predictors across the predicted outcomes in our study, we employed statistical analysis, utilizing both non-parametric and categorical data analysis techniques. For numerical features, the Mann-Whitney U test was applied to compare distributions between 2 independent groups defined by the outcome variables. Each feature's mean value for both outcome classes was calculated to quantify the average influence of the feature within each group. For categorical features, such as gender, care level code, and urgency code, we conducted chi-square tests of independence.

## Results

### Patient characteristics

The dataset’s mean patient age was 63.6 years, with a near-equal gender distribution (47.4% female, 52.5% male). To provide a detailed description of the study population, we include Table 1, summarizing the patients' demographics and key clinical characteristics included in the analysis. These characteristics are based on the entire dataset encompassing all care episodes recorded from 1999 to 2020, thus providing a comprehensive overview of the patient population and their interactions with the healthcare system.

**Table 1.** Summary of patient and care episode characteristics (1999-2020).

Characteristics	Value
Total patients	35 591
Age (mean)	63.6
Sex (male%/female%)	52.5/47.4
Median length of stays (days)	0.41
ICU admissions (%)	66.2
30-day mortality (%)	20.8
30-day readmission (%)	10.4
Number of blood cultures	72 495

### Model evaluation

Table 2 depicts the performance metrics for 30-day mortality prediction at discharge and admission. To predict 30-day mortality at the end of the episode, the XGBoost, LightGBM, and CatBoost models demonstrated superior performance with AUROCs ranging from 0.9600 to 0.9625. The XGBoost model achieved the highest AUPRC and F1 score of 0.8575 and 0.7552. Similarly, for predicting 30-day mortality at the start of the episode, the tree-based model AUROCs ranged from 0.9515 to 0.9545. The LightGBM model achieved an AUPRC of 0.8419 and a recall of 0.7324.

**Table 2.** Model performance metrics for the mortality prediction tasks.

Models	Accuracy	Precision	Recall	F1 score	Specificity	AUPRC	AUROC (95% CI)
***30-day mortality prediction at discharge***							
**XGBoost**	0.9130	0.8221	0.7478	0.7832	0.9570	0.8524	0.9600 (0.9553-0.9642)
**LightGBM**	0.9168	0.8332	0.7552	0.7923	0.9598	0.8575	0.9625 (0.9580-0.9666)
**Catboost**	0.9177	0.8399	0.7512	0.7931	0.9619	0.8566	0.9625 (0.9580-0.9667)
**ANN**	0.8911	0.7571	0.7090	0.7323	0.9395	0.8243	0.9322 (0.9251-0.9394)
**RF**	0.9056	0.8532	0.6649	0.7474	0.9696	0.8172	0.9543 (0.9493-0.9591)
**LR**	0.8320	0.6459	0.4428	0.5254	0.9355	0.6891	0.8257 (0.8133-0.8381)
***30-day mortality prediction at admission***							
**XGBoost**	0.9004	0.7885	0.7184	0.7518	0.9488	0.8336	0.9515 (0.9464-0.9564)
**LightGBM**	0.9055	0.8004	0.7324	0.7649	0.9515	0.8419	0.9540 (0.9493-0.9588)
**Catboost**	0.9059	0.8031	0.7311	0.7654	0.9523	0.8417	0.9545 (0.9494-0.9592)
**ANN**	0.9592	0.6794	0.6776	0.6785	0.9150	0.7963	0.9099 (0.9022-0.9181)
**RF**	0.8893	0.8126	0.6147	0.6999	0.9623	0.7885	0.9459 (0.9403-0.9513)
**LR**	0.8137	0.5865	0.3833	0.4636	0.9282	0.6557	0.7901 (0.7765-0.8029)

Table 3 depicts the performance metrics for the readmission and PLOS prediction tasks. The task of predicting readmission risk saw the CatBoost model reaching an AUROC of 0.8198, accompanied by a 95% CI of 0.8064 to 0.8325, but the F1-score and Recall were low for all the models. Lastly, for the prediction of PLOS at the start of the episode, the LightGBM model proved highly effective, with an AUROC of 0.9797 and AUPRC of 0.9365. In all the prediction tasks, XGBoost, LightGBM, and CatBoost performed better than the traditional models like ANN, RF, and LR, with LR giving the worst overall performance.

**Table 3.** Metrics for predicting 30-day readmission and prolonged length of stay.

Models	Accuracy	Precision	Recall	F1 score	Specificity	AUPRC	AUROC (95% CI)
***30-day readmission prediction at discharge***							
**XGBoost**	0.8460	0.5867	0.2803	0.3794	0.9602	0.6202	0.8001 (0.7867-0.8133)
**LightGBM**	0.8525	0.6593	0.2510	0.3636	0.9738	0.6124	0.8118 (0.7982-0.8245)
**Catboost**	0.8563	0.6886	0.2628	0.3804	0.9760	0.6194	0.8198 (0.8064-0.8325)
**ANN**	0.8038	0.4029	0.3506	0.3749	0.8952	0.6229	0.6839 (0.6660-0.7024)
**RF**	0.8449	0.7333	0.1197	0.2058	0.9912	0.5554	0.8009 (0.7864-0.8143)
**LR**	0.8278	0.4124	0.0611	0.1064	0.9824	0.5218	0.6141 (0.5968-0.6294)
***PLOS prediction at admission***							
**XGBoost**	0.9286	0.8200	0.9179	0.8662	0.9322	0.9251	0.9779 (0.9750-0.9809)
**LightGBM**	0.9313	0.8115	0.9470	0.8740	0.9261	0.9365	0.9797 (0.9770-0.9822)
**Catboost**	0.9310	0.8137	0.9414	0.8729	0.9276	0.9345	0.9794 (0.9767-0.9821)
**ANN**	0.8941	0.7783	0.8096	0.7937	0.9225	0.8660	0.9551 (0.9504-0.9593)
**RF**	0.9237	0.8050	0.9196	0.8585	0.9251	0.9224	0.9733 (0.9702-0.9764)
**LR**	0.7665	0.5931	0.2295	0.3309	0.9471	0.5883	0.7388 (0.7251-0.7513)

### Model explanations for the four prediction tasks

**Figure 2.**
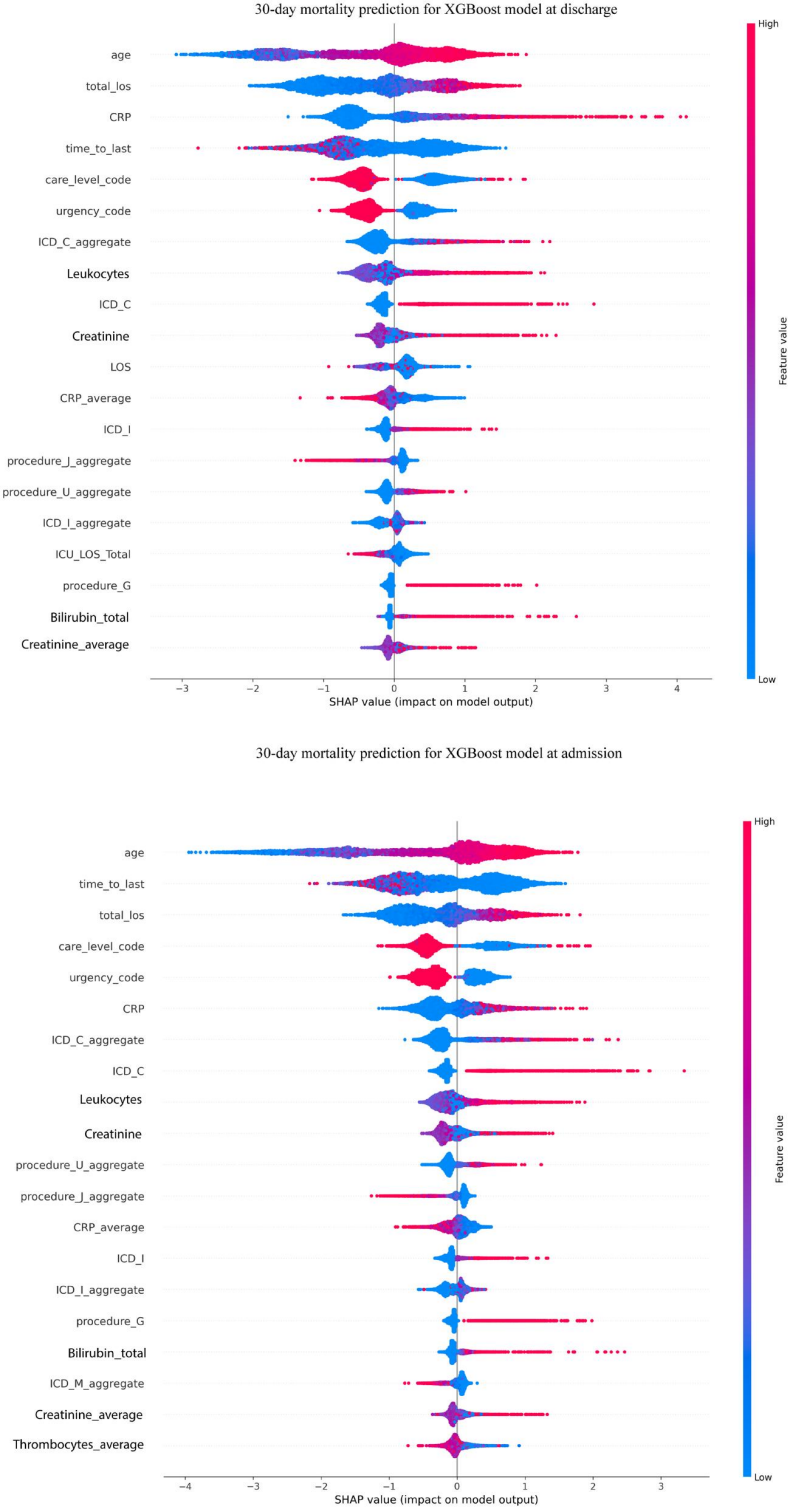
The SHAP summary plots for 30-day mortality prediction at the episode's discharge (top) and admission (bottom). Beeswarm plot detailing the individual SHAP values for each feature and their impact on the model's output.

Figure 2 depicts the SHAP summary plots for 30-day mortality prediction at discharge and admission. For the 30-day mortality prediction at the end of the episode, important features such as age, total length of stay (total_los), CRP levels, and time to the most recent hospital admission (time_to_last) stand out. High values of CRP and older age are associated with an increased risk of mortality, as indicated by the accumulation of red dots on the right side of the zero line. Similarly, in predicting 30-day mortality at the start of the episode, age and total length of stay are prominent, and in predicting 30-day mortality at the end of the episode, age and time to the most recent hospital admission are prominent. The SHAP summary plot highlights the consistent significance of these features across the different stages of hospitalization. Additionally, care level codes and the urgency of the case are influential, suggesting that more acute presentations are associated with higher mortality risk.

**Figure 3.**
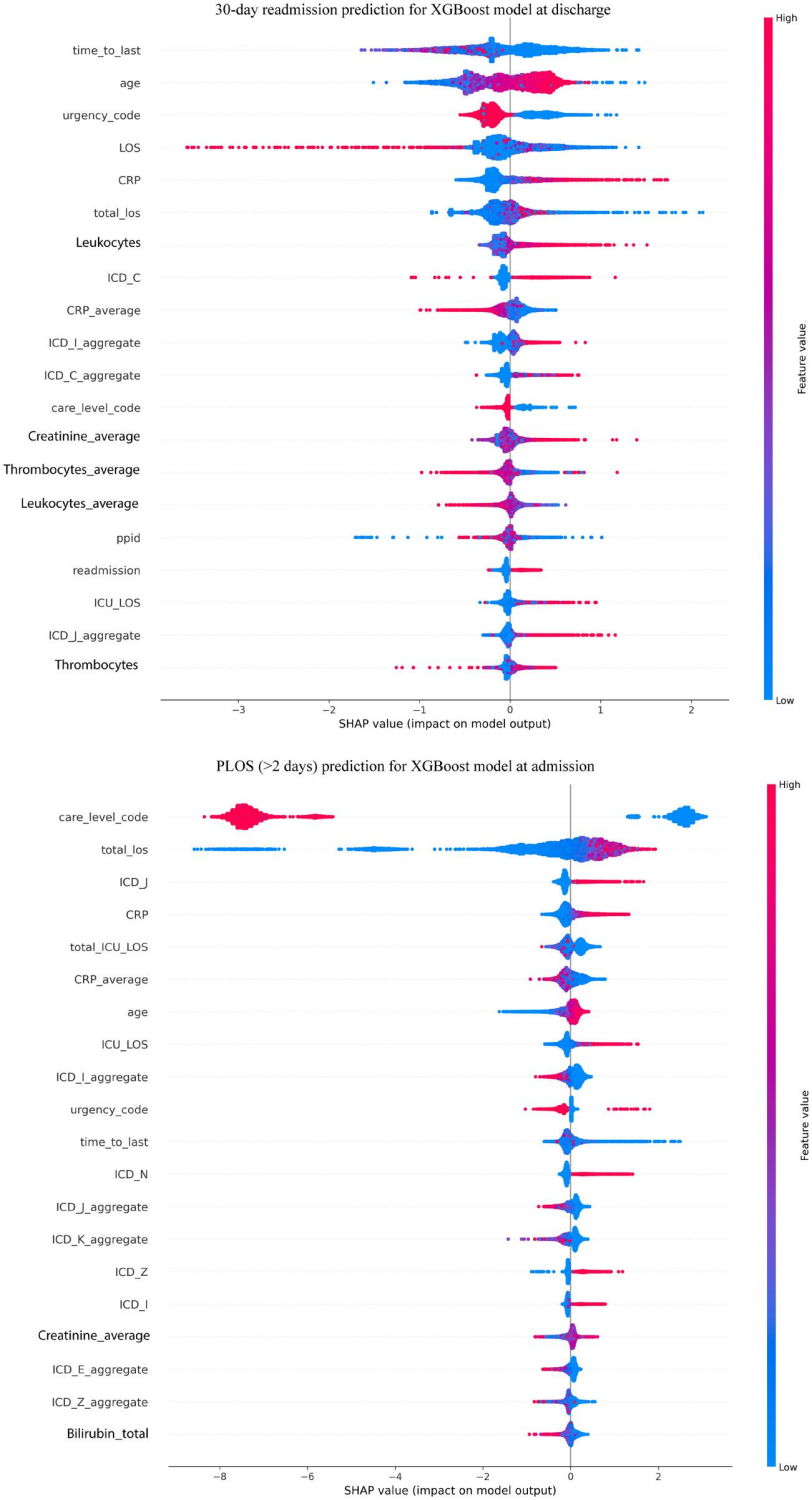
The SHAP summary plots for readmission risk at discharge (top) and PLOS risk at admission (bottom). Beeswarm plot detailing the individual SHAP values for each feature and their impact on the model’s output.

Figure 3 depicts the SHAP summary plots for the readmission risk at discharge and PLOS risk at admission. The readmission risk prediction at the end of the episode emphasizes features like time to the most recent hospital admission, age, urgency code, LOS, CRP levels, leukocyte count, and previous ICD codes related to infections (ICD_C). For the prediction of prolonged length of stay (PLOS) at the start of the episode, features like care level code, total LOS (total_los), ICD codes of ICD chapter X (J00-J99) concerning Diseases of the respiratory system, CRP levels, and ICU length of stay (total_ICU_LOS) provide substantial predictive power. The impact of higher care levels and previous high CRP levels indicate a potentially more complicated hospital course, leading to more extended stays.

### Results of statistical analysis

Notably, age, the total length of previous stays, time to the recent episodes, CRP levels, urgency code, and care level code were identified as significant numerical predictors with a profound impact on the risk assessments for 30-day mortality, readmission, and PLOS. Supplementary Table 1 gives the mean values of the top 10 most significant predictors for the 4 prediction tasks (see Supplementary material S1). The complete statistical analysis is detailed in Tables S3-S6, and Table S2 describes each predictor (see Supplementary material S2). The distribution of predictors across different outcome scenarios provided insightful revelations. For instance, the statistical analysis underscored a strong association between higher CRP levels, advanced age, and an increased risk of 30-day mortality and PLOS, which aligns with clinical expectations. Similarly, the analysis of categorical predictors, such as urgency and care level codes, unveiled significant associations with the outcomes, thus providing a deeper understanding of the factors influencing patient risk profiles. Certain predictors, such as total length of stay, are associated with an increased risk of adverse outcomes. It is important to clarify that “total length of stay” refers to the cumulative length of all previous hospital admissions up to but not including the current hospital admission. This measure helps capture the patient's overall burden of hospitalization before the current episode, which can be an essential factor in predicting outcomes such as 30-day mortality and PLOS. To further investigate the impact of demographic features on model performance and to address potential biases, we conducted ablation studies where we systematically removed demographic features from the models and observed the changes in performance. Specifically, we assessed the following scenarios: When age was excluded from the models, we observed a noticeable decrease in performance for all prediction tasks, as reflected in lower AUROC scores in Table 4. This confirmed that age is a critical factor for predicting adverse outcomes in the hospital. Removing sex from the models did not significantly change performance metrics. This aligns with our SHAP analysis, which indicated that sex was not a crucial feature for our predictions. Removing age and sex together also led to a decline in performance, although the impact was primarily due to the exclusion of age.

**Table 4.** The results of the ablation studies.

Task model: XGBoost	AUROC (age and sex)	AUROC (without age)	AUROC (without sex)	AUROC (without age and sex)
**30-day mortality prediction at discharge** (Task 1)	0.96	0.88	0.95	0.87
**30-day mortality prediction at admissions** (Task 2)	0.95	0.87	0.94	0.87
**30-day readmission prediction at discharge** (Task 3)	0.80	0.76	0.80	0.75
**PLOS prediction at admission** (Task 4)	0.97	0.94	0.97	0.93

## Discussion

The main findings of this study highlight the predictive capacity of medical history features for mortality and PLOS. Across all 4 prediction tasks, the tree-based models have consistently outperformed traditional ML models’ efficiency, underscoring the superiority of tree-based models on tabular data.^44^ The performance for predicting mortality saw a marginal decrease from the episode's conclusion to the start of the episode, underscoring the critical role of current episode information in forecasting mortality outcomes. Moreover, readmission prediction results could have been more impressive when compared with the other 3 tasks. This aligns with the broader literature, suggesting a lack of strong association between hospital readmission rates and mortality across various conditions, affirming the distinct pathways influencing mortality and readmission outcomes.^45^^,^^46^ Our findings on simultaneous good results for mortality and PLOS prediction tasks are consistent with the findings on positive correlations between these 2 events at patient and hospital levels.^47^ While our models have identified various predictors associated with an increased risk of 30-day mortality, readmission, and PLOS, it is important to note that these relationships are associative rather than causal. This means that while certain factors are statistically linked to higher risks, they do not necessarily cause adverse outcomes directly.

This study presented a comprehensive and accurate data modeling methodology compared to techniques such as temporal sequence modeling and patient data simulations, which may introduce unnecessary bias in the original data using imputations.^48^ Our methodology provides a more detailed analysis at the individual patient level by including the entire medical history in the analysis, compared to similar studies that made significant contributions to this field.^16–18^ Cai et al. developed a BN model that, while impressive, achieved slightly lower accuracy and AUROC values compared to our models for mortality prediction.^16^ They provided a daily prediction using features from recent and current medical episodes to predict outcomes of the next episode; in contrast, we included all the synthesized information from the complete medical history available to predict impending adverse outcomes. While Tavakolian et al's GAOCNN approach to predicting hospital readmission and LOS using specific disease population datasets achieved high accuracy,^17^ our study's emphasis on ease of adoption and explainability provides more foundational value for clinical decision-making processes. The multistate model by Clark et al. for surgical patients offers a comprehensive view of hospital quality of care.^18^ Our framework complements such models by harnessing predictors of adverse hospital outcomes from the medical history of the general hospital population. The comparison with other works is intended to provide contextual insights rather than direct equivalence. By highlighting similarities and differences, we aim to offer a broader understanding of the current state of predictive modeling in healthcare. The predictive models developed in this study can be applied to all hospitalized patients with available EPRs. While our analysis focused on patients with at least one blood culture taken over 5 years, the framework is scalable to utilize additional relevant data to predict adverse events across diverse patient populations. A vital consideration in our study is the hierarchical relationship between 30-day mortality and 30-day readmission. To address this, we selected different index events for these predictions. For 30-day readmission prediction, we used the second last episode as the index event, ensuring that the patient was alive during the index event. This approach removed the possibility of including patients who died within 30 days in the readmission analysis. The framework is designed to be compatible with existing hospital information systems, facilitating seamless integration into current workflows without requiring substantial changes to infrastructure or extensive training for healthcare professionals. This compatibility ensures that the framework can be quickly and efficiently implemented in diverse healthcare settings, promoting widespread use and maximizing its impact on patient care.

Our feature engineering process focused on creating robust features, such as cumulative length of stay and time to most recent admission, which are less sensitive to variations in data collection practices. These measures and standardized coding systems, such as ICD-10, collectively mitigate data source variability concerns and enhance our predictive models' reliability. The limitation of this study is its reliance on data from a single center. While we have compared our results with those reported in other studies, it is essential to acknowledge that these comparisons have inherent limitations due to differences in data sources. Other works may use datasets from multiple centers or institutions with distinct patient demographics and healthcare delivery models. We have employed rigorous methodologies, including comprehensive data preprocessing and data enhancement, to ensure the robustness of our models. These methodological strengths mitigate some concerns related to data source variability and enhance the reliability of our findings within the context of our single-center dataset. Another limitation of our study is the specific focus on patients with a history of suspected BSIs, which may not fully represent the broader hospitalized population. Patients with BSIs are a significant and clinically important subgroup; however, their characteristics may differ from other patient groups. Future research should validate our framework across more diverse patient populations to ensure generalizability and robustness.

## Conclusions

This study presents a simple and intuitive XAI framework that comprehensively captures the complete medical history of a patient to predict the risk of hospital adverse outcomes accurately. The XRAI framework is the first to significantly enhance the predictive analysis by integrating information stored as diagnostic and procedural ICD-10 codes and deriving novel temporal features capturing critical indicators of individual trajectories. We also demonstrate that tree-based ML models predict these critical healthcare outcomes, particularly XGBoost, CatBoost, and LightGBM. The SHAP values for model explanation provide valuable insights into the framework's decision-making process. Our findings underscore the importance of age, total length of hospital stay, recent CRP levels, care level codes, and time to the most recent hospital admission as significant predictors of patient outcomes. To further enhance the generalizability of our findings and adoption of our framework, we need to validate our framework on administrative datasets of hospitals outside Norway and include more diverse data sources, such as imaging data, genomics, and patient-reported outcomes, in future works.

## Supplementary Material

ooae074_Supplementary_Data

## Data Availability

We have made the code and data used in this study available on a public repository. This includes all scripts for data preprocessing, data enhancement, model training, evaluation, interpretations, and ablation studies. This study’s code, outputs/results, and data statistics are available at https://github.com/EngineerRajeev/Hospital_adverse_outcomes_prediction.git to foster reproducibility. Due to privacy concerns, the original patient data cannot be shared publicly. Instructions on obtaining access to the original dataset (if permissible) are available with the second author.
