# Supplementary material for: In-hospital mortality, readmission, and prolonged length of stay risk prediction leveraging historical electronic patient records
Source: JAMIA Open. 2024 Sep 14;7(3):ooae074. doi: 10.1093/jamiaopen/ooae074 (PMC11401612; doi:10.1093/jamiaopen/ooae074)
Supplement: ooae074_Supplementary_Data [file ooae074_supplementary_data.zip › Supplementary_Information_2.docx]

Inhospital Mortality, Readmission, and Prolonged Length of Stay Risk Prediction Leveraging Historical Electronic Patient Records

Supplementary Information 2

* corresponding author: rajeev.bopche@ntnu.no

**Supplementary Table 2. The features descriptions.**

| **Feature Name** | **Feature Description** |
| --- | --- |
| **urgency_code** | Urgency code for the current epsiode |
| **care_level_code** | Care level code for the current episode |
| **LOS** | Length of Stay of the current episode |
| **time_to_last** | Time to the most recent hospital episode |
| **total_los** | Total cumulative hospital Length of Stay excluding current episode |
| **age** | Age at the time of prediction |
| **prior_comorbidities_counts** | Counts of comorbidities based on the disease groups |
| **Gender** | 1 for male and 0 for female |
| **Readmission** | 1 if the current episode is a readmission. (within 30 days of previous episode) |
| **ICU_LOS_Total** | Total cumulative ICU Length of Stay (LOS) |
| **ICU_LOS** | Recent/Current ICU Length of Stay (LOS) |
| **procedure_A** | Count of procedure codes starting with the letter A in current/recent episode [The nervous system] |
| **procedure_B** | Count of procedure codes starting with the letter B in current/recent episode [Endocrine organs] |
| **procedure_C** | Count of procedure codes starting with the letter C in current/recent episode [The eye and the eye region] |
| **procedure_D** | Count of procedure codes starting with the letter D in current/recent episode [Ear, nose, sinuses and larynx] |
| **procedure_E** | Count of procedure codes starting with the letter E in current/recent episode [Teeth, jaws, mouth and pharynx] |
| **procedure_F** | Count of procedure codes starting with the letter F in current/recent episode [The heart and the great intrathoracic vessels] |
| **procedure_G** | Count of procedure codes starting with the letter G in current/recent episode [Chest wall, pleura, diaphragm, trachea, bronchi, lungs and mediastinum] |
| **procedure_H** | Count of procedure codes starting with the letter H in current/recent episode [Mother] |
| **procedure_I** | Count of procedure codes starting with the letter I in current/recent episode [Mental health and addiction] |
| **procedure_J** | Count of procedure codes starting with the letter J in current/recent episode [Digestive organs and spleen] |
| **procedure_K** | Count of procedure codes starting with the letter K in current/recent episode [Urinary organs, male genitalia and the retroperitoneal space] |
| **procedure_L** | Count of procedure codes starting with the letter L in current/recent episode [Female genitalia] |
| **procedure_M** | Count of procedure codes starting with the letter M in current/recent episode [Birth care and procedures during pregnancy] |
| **procedure_N** | Count of procedure codes starting with the letter N in current/recent episode [The locomotor system] |
| **procedure_O** | Count of procedure codes starting with the letter O in current/recent episode [Habilitation and rehabilitation in the specialist health service, including private rehabilitation institutions with agreement] |
| **procedure_P** | Count of procedure codes starting with the letter P in current/recent episode [Peripheral vessels and the lymphatic system] |
| **procedure_Q** | Count of procedure codes starting with the letter Q in current/recent episode [Hud] |
| **procedure_R** | Count of procedure codes starting with the letter R in current/recent episode [Blood with components] |
| **procedure_S** | Count of procedure codes starting with the letter S in current/recent episode [Imaging examinations] |
| **procedure_T** | Count of procedure codes starting with the letter T in current/recent episode [Nuclear medicine] |
| **procedure_W** | Count of procedure codes starting with the letter W in current/recent episode [Measures not classified in other chapters] |
| **procedure_Y** | Count of procedure codes starting with the letter Y in current/recent episode [Removal of organs or tissues for transplantation] |
| **procedure_Z** | Count of procedure codes starting with the letter Z in current/recent episode [Additional codes] |
| **ICD_A** | Count of Diagnostic codes starting with the letter A in current/recent episode [Certain infectious and parasitic diseases] |
| **ICD_B** | Count of Diagnostic codes starting with the letter B in current/recent episode [Certain infectious and parasitic diseases] |
| **ICD_C** | Count of Diagnostic codes starting with the letter C in current/recent episode [Cancer disease] |
| **ICD_D** | Count of Diagnostic codes starting with the letter D in current/recent episode [Diseases of the blood and blood-forming organs and certain conditions involving the immune system] |
| **ICD_E** | Count of Diagnostic codes starting with the letter E in current/recent episode [Endocrine diseases, nutritional diseases and metabolic disorders] |
| **ICD_F** | Count of Diagnostic codes starting with the letter F in current/recent episode [Mental disorders and behavioral disorders] |
| **ICD_G** | Count of Diagnostic codes starting with the letter G in current/recent episode [Diseases of the nervous system] |
| **ICD_H** | Count of Diagnostic codes starting with the letter H in current/recent episode [Diseases of the eye and ears] |
| **ICD_I** | Count of Diagnostic codes starting with the letter I in current/recent episode [Diseases of the circulatory system] |
| **ICD_J** | Count of Diagnostic codes starting with the letter J in current/recent episode [Diseases of the respiratory system] |
| **ICD_K** | Count of Diagnostic codes starting with the letter K in current/recent episode [Diseases of the digestive system] |
| **ICD_L** | Count of Diagnostic codes starting with the letter L in current/recent episode [Diseases of the skin and subcutaneous tissue] |
| **ICD_M** | Count of Diagnostic codes starting with the letter M in current/recent episode [Diseases of the musculoskeletal system and connective tissue] |
| **ICD_N** | Count of Diagnostic codes starting with the letter N in current/recent episode [Diseases of the urinary and genital organs] |
| **ICD_O** | Count of Diagnostic codes starting with the letter O in current/recent episode [Pregnancy, birth and maternity] |
| **ICD_P** | Count of Diagnostic codes starting with the letter P in current/recent episode [Certain conditions that occur in the perinatal period] |
| **ICD_Q** | Count of Diagnostic codes starting with the letter Q in current/recent episode [Congenital malformations, deformities and chromosomal abnormalities] |
| **ICD_R** | Count of Diagnostic codes starting with the letter R in current/recent episode [Symptoms, signs, abnormal clinical and laboratory findings, not elsewhere classified] |
| **ICD_S** | Count of Diagnostic codes starting with the letter S in current/recent episode [Injuries] |
| **ICD_T** | Count of Diagnostic codes starting with the letter T in current/recent episode [Poisonings and certain other consequences of external causes] |
| **ICD_U** | Count of Diagnostic codes starting with the letter U in current/recent episode [Codes for special purposes] |
| **ICD_V** | Count of Diagnostic codes starting with the letter V in current/recent episode [External causes of diseases, injuries and deaths] |
| **ICD_W** | Count of Diagnostic codes starting with the letter W in current/recent episode [External causes of diseases, injuries and deaths] |
| **ICD_X** | Count of Diagnostic codes starting with the letter X in current/recent episode [External causes of diseases, injuries and deaths] |
| **ICD_Y** | Count of Diagnostic codes starting with the letter Y in current/recent episode [External causes of diseases, injuries and deaths] |
| **ICD_Z** | Count of Diagnostic codes starting with the letter Z in current/recent episode [Factors that have an impact on health status and contact with the health service] |
| **urinarytractinfection** | Count of urinary tract infection episodes in the medical history |
| **cardiovascular** | Count of cardiovascular disease episodes |
| **lung** | Count of lung disease episodes |
| **centralnervoussystem** | Count of CNS disease episodes |
| **organdysfunction** | Count of organdysfunction episodes |
| **skinandsofttissueinfection** | Count of the skin and soft tissue infection episodes |
| **pneumonia** | Count of the pneumonia episodes |
| **endocarditis** | Count of the endocarditis episodes |
| **sepsis** | Count of the sepsis episodes |
| **infection** | Count of the infection episodes |
| **dementia** | Count of the dementia episodes |
| **explicitsepsis** | Count of the explicit sepsis episodes |
| **intraabdominalinfection** | Count of the intra abdominal infection episodes |
| **cancer** | Count of the cancer disease episodes |
| **BILIRUBIN KONJUGERT_average** | Bilirubin Laboratory tests results overall average |
| **BILIRUBIN TOTAL_average** | Bilirubin Laboratory tests results overall average |
| **BILIRUBIN UKONJUGERT_average** | Bilirubin Laboratory tests results overall average |
| **CRP_average** | CRP Laboratory tests results overall average |
| **CRP-HØYSENSITIV_average** | CRP Laboratory tests results overall average |
| **KREATININ_average** | Creatinine Laboratory tests results overall average |
| **LAKTAT_average** | Lactate Laboratory tests results overall average |
| **LAKTAT BLODGASS_average** | Lactate blood gas Laboratory tests results overall average |
| **LAKTAT BLODGASS VENØST_average** | Lactate blood gas venous Laboratory tests results overall average |
| **LAKTAT PNA_average** | Lactate PNA Laboratory tests results overall average |
| **LEUKOCYTTER_average** | Leukocytes Laboratory tests results overall average |
| **PH_average** | PH Laboratory tests results overall average |
| **PH PNA_average** | PH PNA Laboratory tests results overall average |
| **PO2_average** | PO2 Laboratory tests results overall average |
| **PO2 PNA_average** | PO2 PNA Laboratory tests results overall average |
| **TROMBOCYTTER_average** | Thrombocytes Laboratory tests results overall average |
| **BILIRUBIN KONJUGERT** | Bilirubin Conjugate latest Laboratory tests results |
| **BILIRUBIN TOTAL** | Bilirubin total latest Laboratory tests results |
| **BILIRUBIN UKONJUGERT** | Bilirubin Unconjugate latest Laboratory tests results |
| **CRP** | CRP latest Laboratory tests results |
| **CRP-HØYSENSITIV** | Highly sensitive CRP latest Laboratory tests results |
| **KREATININ** | Creatinine latest Laboratory tests results |
| **LAKTAT** | Lactate latest Laboratory tests results |
| **LAKTAT BLODGASS** | Latest Laboratory tests results |
| **LAKTAT BLODGASS VENØST** | Latest Laboratory tests results |
| **LAKTAT PNA** | Latest Laboratory tests results |
| **LEUKOCYTTER** | Leukocytes latest Laboratory tests results |
| **PH** | PH latest Laboratory tests results |
| **PH PNA** | PH PNA latest Laboratory tests results |
| **PO2** | PO2 latest Laboratory tests results |
| **PO2 PNA** | PO2 PNA latest Laboratory tests results |
| **TROMBOCYTTER** | Thrombocytes latest Laboratory tests results |
| **Positive_annet_test** | Total number of previous positive results see (Supplementary List 1) for the groups of microbiology tests by collection sample. |
| **Positive_anus_test** | Total number of previous positive results see (Supplementary List 1) for the groups of microbiology tests by collection sample. |
| **Positive_bein_test** | Total number of previous positive results see (Supplementary List 1) for the groups of microbiology tests by collection sample. |
| **Positive_biopsi_test** | Total number of previous positive results see (Supplementary List 1) for the groups of microbiology tests by collection sample. |
| **Positive_blod_test** | Total number of previous positive results see (Supplementary List 1) for the groups of microbiology tests by collection sample. |
| **Positive_blood_culture_test_test** | Total number of previous positive results see (Supplementary List 1) for the groups of microbiology tests by collection sample. |
| **Positive_bronki_test** | Total number of previous positive results see (Supplementary List 1) for the groups of microbiology tests by collection sample. |
| **Positive_melk_test** | Total number of previous positive results see (Supplementary List 1) for the groups of microbiology tests by collection sample. |
| **Positive_edta_test** | Same as above |
| **Positive_faeces_test** | Same as above |
| **Positive_hal_test** | Same as above |
| **Positive_hud_test** | Same as above |
| **Positive_led_test** | Same as above |
| **Positive_naso_test** | Same as above |
| **Positive_plasma_test** | Same as above |
| **Positive_tunge_test** | Same as above |
| **Positive_urin_test** | Same as above |
| **Positive_ear_test** | Same as above |
| **Positive_eye_test** | Same as above |
| **ICD_A_aggregate** | Cumulative count in the complete history |
| **ICD_B_aggregate** | Cumulative count in the complete history |
| **ICD_C_aggregate** | Cumulative count in the complete history |
| **ICD_D_aggregate** | Cumulative count in the complete history |
| **ICD_E_aggregate** | Cumulative count in the complete history |
| **ICD_F_aggregate** | Cumulative count in the complete history |
| **ICD_G_aggregate** | Cumulative count in the complete history |
| **ICD_H_aggregate** | Cumulative count in the complete history |
| **ICD_I_aggregate** | Cumulative count in the complete history |
| **ICD_J_aggregate** | Cumulative count in the complete history |
| **ICD_K_aggregate** | Cumulative count in the complete history |
| **ICD_L_aggregate** | Cumulative count in the complete history |
| **ICD_M_aggregate** | Cumulative count in the complete history |
| **ICD_N_aggregate** | Cumulative count in the complete history |
| **ICD_O_aggregate** | Cumulative count in the complete history |
| **ICD_P_aggregate** | Cumulative count in the complete history |
| **ICD_Q_aggregate** | Cumulative count in the complete history |
| **ICD_R_aggregate** | Cumulative count in the complete history |
| **ICD_S_aggregate** | Cumulative count in the complete history |
| **ICD_T_aggregate** | Cumulative count in the complete history |
| **ICD_U_aggregate** | Cumulative count in the complete history |
| **ICD_V_aggregate** | Cumulative count in the complete history |
| **ICD_W_aggregate** | Cumulative count in the complete history |
| **ICD_X_aggregate** | Cumulative count in the complete history |
| **ICD_Y_aggregate** | Cumulative count in the complete history |
| **ICD_Z_aggregate** | Cumulative count in the complete history |
| **procedure_A_aggregate** | Cumulative count in the complete history |
| **procedure_B_aggregate** | Cumulative count in the complete history |
| **procedure_C_aggregate** | Cumulative count in the complete history |
| **procedure_D_aggregate** | Cumulative count in the complete history |
| **procedure_E_aggregate** | Cumulative count in the complete history |
| **procedure_F_aggregate** | Cumulative count in the complete history |
| **procedure_G_aggregate** | Cumulative count in the complete history |
| **procedure_H_aggregate** | Cumulative count in the complete history |
| **procedure_I_aggregate** | Cumulative count in the complete history |
| **procedure_J_aggregate** | Cumulative count in the complete history |
| **procedure_K_aggregate** | Cumulative count in the complete history |
| **procedure_L_aggregate** | Cumulative count in the complete history |
| **procedure_M_aggregate** | Cumulative count in the complete history |
| **procedure_N_aggregate** | Cumulative count in the complete history |
| **procedure_O_aggregate** | Cumulative count in the complete history |
| **procedure_P_aggregate** | Cumulative count in the complete history |
| **procedure_Q_aggregate** | Cumulative count in the complete history |
| **procedure_R_aggregate** | Cumulative count in the complete history |
| **procedure_T_aggregate** | Cumulative count in the complete history |
| **procedure_U_aggregate** | Cumulative count in the complete history |
| **procedure_W_aggregate** | Cumulative count in the complete history |
| **procedure_X_aggregate** | Cumulative count in the complete history |
| **procedure_Y_aggregate** | Cumulative count in the complete history |
| **procedure_Z_aggregate** | Cumulative count in the complete history |

**Supplementary Table 3. Sorted statistical summary table for 30-day mortality predictors at discharge**

|  | **Stat** | **P-Value** | **Mean (Class 0)** | **Mean (Class 1)** | **Chi2 Stat** | **Proportion (Class 0)** | **Proportion (Class 1)** |
| --- | --- | --- | --- | --- | --- | --- | --- |
| **urgency_code** |  | 0.0 |  |  | 3476.8719636480846 | 0.6887445426472154 | 0.6350768401186303 |
| **ICD_J_aggregate** | 75051088.0 | 0.0 | 2.1058460227877753 | 4.130493394445942 |  |  |  |
| **ICD_I_aggregate** | 70409711.5 | 0.0 | 5.215170553366699 | 8.984631976273928 |  |  |  |
| **CRP** | 54503172.5 | 0.0 | 20.847211869520457 | 82.77698391300441 |  |  |  |
| **ICD_C_aggregate** | 66088361.5 | 0.0 | 5.717992404074823 | 21.923159881369642 |  |  |  |
| **procedure_R_aggregate** | 80991734.0 | 0.0 | 1.203457210804671 | 1.8061472094904287 |  |  |  |
| **care_level_code** |  | 0.0 |  |  | 5309.82522571323 | 0.7020906541724345 | 0.6952008627662443 |
| **LOS** | 55965554.0 | 0.0 | 1.1788725020410022 | 5.038571717444051 |  |  |  |
| **time_to_last** | 139810218.5 | 0.0 | 4029.568381074078 | 1528.2231059584794 |  |  |  |
| **LEUKOCYTTER** | 66826795.0 | 0.0 | 5.593829434801651 | 11.083697312842686 |  |  |  |
| **ICD_I** | 78565754.5 | 0.0 | 0.20370567564689596 | 0.746157994068482 |  |  |  |
| **ICD_C** | 71549881.0 | 0.0 | 0.10201256522202108 | 0.6658128875707738 |  |  |  |
| **age** | 55954082.5 | 0.0 | 55.44148652965605 | 75.13345915341063 |  |  |  |
| **total_los** | 55653342.5 | 0.0 | 29.37551319821564 | 55.32891839669267 |  |  |  |
| **ICD_J** | 80707904.0 | 0.0 | 0.14293827423419586 | 0.49015907252628743 |  |  |  |
| **procedure_G** | 92445021.5 | 6.461147808177588e-307 | 0.05373939587548362 | 0.2100296575896468 |  |  |  |
| **KREATININ_average** | 77213942.0 | 3.872568598236364e-263 | 70.35451359054105 | 98.55273301141723 |  |  |  |
| **ICD_D_aggregate** | 81522390.0 | 1.0225342241721986e-248 | 1.3226138501402052 | 2.241978970072796 |  |  |  |
| **procedure_G_aggregate** | 84138332.5 | 2.5276146830778575e-213 | 1.2656799062932595 | 2.038285252089512 |  |  |  |
| **ICD_E_aggregate** | 83236161.5 | 2.527435546142632e-204 | 2.262733823164022 | 2.9621191695874898 |  |  |  |
| **procedure_U_aggregate** | 84930257.0 | 9.117993254886273e-203 | 0.7292798069073226 | 1.3982205446211917 |  |  |  |
| **prior_comorbidities_counts** | 82260989.5 | 1.0661305606478129e-197 | 0.88620310226103 | 1.2905095713130224 |  |  |  |
| **Positive_urin_test** | 83689866.5 | 3.4100969888982916e-182 | 1.5700493380186704 | 2.1791588029118363 |  |  |  |
| **cancer** | 93554126.5 | 8.975823485430375e-180 | 0.06680154758101729 | 0.18172014019951468 |  |  |  |
| **procedure_R** | 98428871.0 | 3.090305100186988e-166 | 0.015582295105242608 | 0.08344567268805608 |  |  |  |
| **KREATININ** | 84065630.0 | 9.475821574673034e-152 | 55.175925945645346 | 95.89530825918935 |  |  |  |
| **procedure_P_aggregate** | 90861981.0 | 9.09574276985113e-142 | 0.33454016256699676 | 0.6221353464545699 |  |  |  |
| **cardiovascular** | 89793234.5 | 1.5143070950098356e-134 | 0.24228871614666525 | 0.3968724723645187 |  |  |  |
| **Positive_edta_test** | 95068501.0 | 1.990367851231134e-127 | 0.1289177581372236 | 0.2961714747910488 |  |  |  |
| **procedure_W_aggregate** | 86694332.0 | 4.942459850275391e-125 | 3.9896354665814786 | 7.32704232946886 |  |  |  |
| **ICD_N** | 93989762.0 | 1.2466367731610224e-119 | 0.11560714158946508 | 0.23726071717444056 |  |  |  |
| **ICD_E** | 95326940.5 | 6.173932779419837e-118 | 0.08564938061264331 | 0.20490698301428956 |  |  |  |
| **BILIRUBIN TOTAL** | 92208196.5 | 6.624664274016081e-117 | 3.4652740803842925 | 9.925024714658038 |  |  |  |
| **CRP_average** | 86630808.5 | 5.239982296305585e-114 | 55.51249149748861 | 68.26720536652978 |  |  |  |
| **procedure_T_aggregate** | 90388872.0 | 3.177158173687538e-113 | 0.6153764242359706 | 1.0806147209490429 |  |  |  |
| **ICD_N_aggregate** | 88194014.5 | 5.312688476904842e-110 | 3.7141589465090687 | 7.733620922081424 |  |  |  |
| **procedure_P** | 101035132.0 | 1.8426844194823795e-108 | 0.007631420154048202 | 0.05217039633324346 |  |  |  |
| **procedure_Y_aggregate** | 102541509.5 | 8.605980998851064e-105 | 0.0004969296844496504 | 0.028174710164464815 |  |  |  |
| **procedure_Y** | 102736544.5 | 2.316684852241728e-104 | 3.549497746068931e-05 | 0.02386087894311135 |  |  |  |
| **Positive_blood_culture_test_test** | 95138371.0 | 7.578312641078385e-102 | 0.11745288041742094 | 0.2537071987058506 |  |  |  |
| **ICD_O_aggregate** | 110650265.0 | 1.5626028601862474e-96 | 0.49135697298832215 | 0.009706120248045295 |  |  |  |
| **procedure_M_aggregate** | 110566746.5 | 8.144818502358003e-96 | 0.46778830795442444 | 0.012671879212725803 |  |  |  |
| **procedure_F_aggregate** | 90623111.5 | 7.971645053399038e-94 | 1.4430838036417848 | 1.916149905634942 |  |  |  |
| **ICU_LOS_Total** | 89041514.0 | 1.5579416093539213e-89 | 1.9911159029330665 | 3.2515221982565077 |  |  |  |
| **ICD_A_aggregate** | 92787483.5 | 4.850371328140499e-89 | 0.38441060589926523 | 0.5923429495820977 |  |  |  |
| **ICD_Z** | 93957829.0 | 6.706717631485326e-82 | 0.19075000887374435 | 0.3025074143974117 |  |  |  |
| **Positive_hal_test** | 98708929.0 | 7.892420251186258e-77 | 0.05359741596564086 | 0.12901051496360205 |  |  |  |
| **ICD_A** | 99770196.5 | 1.52009926562003e-71 | 0.03041919568381074 | 0.0764356969533567 |  |  |  |
| **Positive_plasma_test** | 96860961.0 | 6.1160736593606e-70 | 0.15855606431689917 | 0.3034510649770828 |  |  |  |
| **ICU_LOS** | 90988090.5 | 6.596822692764581e-70 | 0.7053310498231149 | 1.5972521793834782 |  |  |  |
| **ICD_D** | 99455368.0 | 5.680102566866028e-67 | 0.03911546516167962 | 0.09989215421946616 |  |  |  |
| **PH_average** | 110996020.5 | 1.4136239744891743e-63 | 0.7421480177940367 | 0.28555532318614063 |  |  |  |
| **procedure_T** | 102468272.0 | 1.458061868973886e-62 | 0.004188407340361339 | 0.025208951199784307 |  |  |  |
| **ICD_P_aggregate** | 109021797.0 | 6.802837309796926e-62 | 0.3350725872289071 | 0.04286869776220006 |  |  |  |
| **organdysfunction** | 97949570.0 | 1.6610888574467235e-57 | 0.09239342633017428 | 0.15772445403073604 |  |  |  |
| **ICD_Z_aggregate** | 92240451.5 | 1.6101120291431334e-55 | 8.39953146629752 | 13.943246157994068 |  |  |  |
| **LAKTAT BLODGASS_average** | 108530444.0 | 2.1309691051356098e-54 | 0.0844358944540068 | 0.018023595363715325 |  |  |  |
| **ICD_M** | 109881934.0 | 1.711999836259085e-51 | 0.09125758705143222 | 0.03531949312483149 |  |  |  |
| **ICD_R_aggregate** | 92940110.0 | 2.996441892051741e-51 | 2.7780854009157703 | 2.8854138581827984 |  |  |  |
| **ICD_H** | 108544372.5 | 1.7543104142969675e-49 | 0.05530117488375395 | 0.011863035858722027 |  |  |  |
| **ICD_F** | 100167796.0 | 6.506232912619054e-47 | 0.048805594008447806 | 0.09355621461310326 |  |  |  |
| **Positive_bronki_test** | 101868686.0 | 2.357204198734685e-44 | 0.016150214744613638 | 0.045969263952547856 |  |  |  |
| **LEUKOCYTTER_average** | 93662522.5 | 4.56726134117201e-43 | 8.708393988205863 | 9.692758263061663 |  |  |  |
| **procedure_X_aggregate** | 101497771.5 | 3.882770842424184e-37 | 0.02903489156284386 | 0.0637638177406309 |  |  |  |
| **PH** | 107260758.5 | 1.6351621965853378e-36 | 0.23660756752919415 | 0.04115462388784039 |  |  |  |
| **BILIRUBIN TOTAL_average** | 94705696.0 | 8.210433321430327e-36 | 14.062026597157073 | 13.774103774248708 |  |  |  |
| **infection** | 96302059.5 | 4.3075193822821885e-35 | 0.396549888190821 | 0.49770827716365595 |  |  |  |
| **ICD_Q_aggregate** | 108181231.0 | 2.187826908231105e-32 | 0.4477691406665957 | 0.1275276354812618 |  |  |  |
| **dementia** | 102049888.0 | 1.430375398988515e-31 | 0.019912682355446704 | 0.04367754111620383 |  |  |  |
| **procedure_D** | 106844171.0 | 1.1024833607018874e-28 | 0.04319738756965889 | 0.009301698571043408 |  |  |  |
| **procedure_C** | 106377213.5 | 3.708607221870702e-28 | 0.027047172825045256 | 0.0018873011593421407 |  |  |  |
| **ICD_F_aggregate** | 98508343.5 | 6.300614687658106e-26 | 3.6310297092961346 | 2.371528713939067 |  |  |  |
| **ICD_K_aggregate** | 96903020.5 | 3.4506788715844646e-25 | 2.7748908529443086 | 2.577514154758695 |  |  |  |
| **procedure_U** | 103359168.5 | 1.0006194954820554e-23 | 0.004756326979732368 | 0.016985710434079267 |  |  |  |
| **procedure_O** | 106600839.5 | 1.0802149080806325e-23 | 0.039186455116601 | 0.010245349150714478 |  |  |  |
| **TROMBOCYTTER_average** | 112253578.0 | 6.297300651517788e-23 | 240.82809185651757 | 230.0840842441166 |  |  |  |
| **Positive_naso_test** | 100421835.0 | 2.3892956241893825e-22 | 0.1282078585880098 | 0.17902399568616878 |  |  |  |
| **ICD_O** | 105716330.0 | 4.987008878310634e-20 | 0.020232137152592908 | 0.0004044216770018873 |  |  |  |
| **procedure_L_aggregate** | 108312258.5 | 3.421784574536348e-19 | 0.3592446668796365 | 0.20463736856295497 |  |  |  |
| **ICD_R** | 100540188.0 | 5.245791812563757e-19 | 0.12011500372697263 | 0.16486923699110273 |  |  |  |
| **LAKTAT BLODGASS VENØST_average** | 105674826.5 | 2.4144969319409624e-17 | 0.02260626942716178 | 0.004740143024047042 |  |  |  |
| **procedure_M** | 105483960.0 | 3.89391677880088e-17 | 0.016185709722074327 | 0.0 |  |  |  |
| **procedure_W** | 101404125.0 | 2.4533262939378747e-16 | 0.08607532034217158 | 0.1365597196009706 |  |  |  |
| **procedure_Z_aggregate** | 110298205.0 | 3.5800802194477833e-16 | 3.829872573030916 | 2.597600431383122 |  |  |  |
| **CRP-HØYSENSITIV** | 105749587.5 | 1.8379629814007103e-15 | 0.14602988677102186 | 0.40281881908870315 |  |  |  |
| **Gender** |  | 3.3766183934485417e-15 |  |  | 62.033511631887876 | 0.5139672736307812 | 0.5653815044486384 |
| **ICD_L** | 106464572.5 | 1.7570879969753303e-14 | 0.044617186668086464 | 0.023321650040442166 |  |  |  |
| **ICD_B_aggregate** | 100296963.5 | 1.3652839047064898e-13 | 0.5155290526390516 | 0.5246697222971152 |  |  |  |
| **procedure_K_aggregate** | 100408301.0 | 2.1931128269290067e-13 | 1.5470485926241437 | 4.92922620652467 |  |  |  |
| **ICD_H_aggregate** | 100033294.5 | 8.440886746405153e-13 | 1.9927590245980193 | 2.0548665408465894 |  |  |  |
| **ICD_W_aggregate** | 102595212.0 | 1.8070273855213364e-12 | 0.041529123629006494 | 0.06457266109463468 |  |  |  |
| **ICD_Y_aggregate** | 102294515.5 | 1.0684703916042478e-11 | 0.07024456039470416 | 0.09112968455109194 |  |  |  |
| **ICD_M_aggregate** | 109235060.5 | 1.5408889040072222e-11 | 3.5540056082064386 | 2.1884605014828793 |  |  |  |
| **sepsis** | 103765887.0 | 1.8332224667866904e-11 | 0.004898306889575125 | 0.011863035858722027 |  |  |  |
| **procedure_L** | 105175953.0 | 5.234422414085725e-11 | 0.010471018350903347 | 0.0012132650310056618 |  |  |  |
| **TROMBOCYTTER** | 100103729.0 | 1.2616789336503937e-09 | 118.09874111146608 | 121.01644648153143 |  |  |  |
| **procedure_O_aggregate** | 108276514.0 | 2.19729290443262e-09 | 3.365704752777482 | 2.3685629549743865 |  |  |  |
| **procedure_F** | 102957228.5 | 5.169091803468203e-09 | 0.04131615376424236 | 0.07400916689134537 |  |  |  |
| **ICD_G** | 102650295.0 | 6.364643323736832e-09 | 0.05583359954566429 | 0.07764896198436236 |  |  |  |
| **procedure_I_aggregate** | 106982345.5 | 1.5476062983633245e-08 | 1.6968373975082527 | 0.9770827716365598 |  |  |  |
| **procedure_A** | 103420001.5 | 2.9039856170592678e-08 | 0.028147517126326623 | 0.040711782151523324 |  |  |  |
| **procedure_C_aggregate** | 101684380.5 | 7.145732828447206e-08 | 1.0571469137117098 | 1.059719600970612 |  |  |  |
| **procedure_E** | 104999648.0 | 7.37792037756632e-08 | 0.00724097540198062 | 0.0010784578053383662 |  |  |  |
| **LAKTAT BLODGASS** | 105120165.5 | 3.067351022151198e-07 | 0.014583111489724205 | 0.01186078907162757 |  |  |  |
| **procedure_B_aggregate** | 105417300.5 | 3.5983887950573667e-07 | 0.034785077911475525 | 0.016042059854408198 |  |  |  |
| **Positive_ear_test** | 105150635.0 | 4.2571542220096327e-07 | 0.014872395556028822 | 0.006605554057697492 |  |  |  |
| **procedure_J** | 106061536.5 | 5.209436631655601e-07 | 0.07123841976360346 | 0.06848207063898624 |  |  |  |
| **ICD_G_aggregate** | 101544903.5 | 8.105848268610126e-07 | 1.64366592127214 | 1.4228902669183068 |  |  |  |
| **explicitsepsis** | 104208325.0 | 1.3854254924359366e-06 | 0.0013843041209668833 | 0.004044216770018873 |  |  |  |
| **ICD_Q** | 105217534.5 | 1.7769583708007634e-06 | 0.01448195080396124 | 0.009301698571043408 |  |  |  |
| **procedure_0** | 104409138.0 | 1.8065877350138772e-06 | 0.0 | 0.0008088433540037746 |  |  |  |
| **procedure_E_aggregate** | 105927272.0 | 9.63951782672537e-06 | 0.11141873424910376 | 0.08708546778107307 |  |  |  |
| **ICD_U** | 104190510.5 | 1.072528293856268e-05 | 0.001952223760337912 | 0.004987867349689944 |  |  |  |
| **ICD_U_aggregate** | 103843306.5 | 2.322706947488587e-05 | 0.02541440386185355 | 0.06484227554596926 |  |  |  |
| **procedure_Q** | 105256300.0 | 3.257405883344266e-05 | 0.023994604763425974 | 0.01712051765974656 |  |  |  |
| **ICD_P** | 105030545.5 | 3.3008008191774997e-05 | 0.01803144855003017 | 0.019681854947425182 |  |  |  |
| **ICD_S_aggregate** | 101948491.0 | 9.759260890999675e-05 | 1.0059631562133957 | 0.9421677001887301 |  |  |  |
| **procedure_A_aggregate** | 106855752.5 | 0.00010726486312645504 | 0.9360380506158379 | 0.8627662442706929 |  |  |  |
| **BILIRUBIN UKONJUGERT_average** | 103899777.0 | 0.0006018164126598498 | 0.648784970555815 | 0.5192190168059675 |  |  |  |
| **ICD_V_aggregate** | 104926858.0 | 0.0006810252641772957 | 0.019948177332907394 | 0.021838770558101916 |  |  |  |
| **BILIRUBIN KONJUGERT_average** | 103837037.0 | 0.0006814873558297512 | 0.6633617027797626 | 1.6190808393996583 |  |  |  |
| **procedure_H_aggregate** | 105171323.0 | 0.0007212888704293516 | 0.043126397614737515 | 0.02480452952278242 |  |  |  |
| **ICD_B** | 103712851.5 | 0.0018690745578963171 | 0.035211017641003796 | 0.04543003504987867 |  |  |  |
| **procedure_J_aggregate** | 102456777.5 | 0.0027657524115532192 | 1.3723068185851701 | 1.5163116743057428 |  |  |  |
| **procedure_X** | 104434351.5 | 0.003718840069792487 | 0.00010648493238206793 | 0.0006740361283364788 |  |  |  |
| **procedure_N_aggregate** | 106353592.5 | 0.004506992072759957 | 1.197990984275725 | 0.9684551091938528 |  |  |  |
| **ICD_L_aggregate** | 102845052.5 | 0.005577842351054158 | 1.6548113441947965 | 1.4595578322998113 |  |  |  |
| **PO2_average** | 104465484.0 | 0.00585046974121691 | 0.0 | 0.002709625235912645 |  |  |  |
| **intraabdominalinfection** | 104605676.5 | 0.008772158504936182 | 0.0012068292336634367 | 0.00013480722566729578 |  |  |  |
| **Positive_faeces_test** | 103887389.5 | 0.009199805290354357 | 0.04089021403471409 | 0.04179023995686169 |  |  |  |
| **Positive_led_test** | 104667341.0 | 0.011212380499635225 | 0.004720832002271679 | 0.0016176867080075492 |  |  |  |
| **ICD_W** | 104328414.5 | 0.013045389208181941 | 0.00205870869271998 | 0.0037746023186842814 |  |  |  |
| **LAKTAT PNA_average** | 104173956.5 | 0.013287299526931213 | 0.011117026940687896 | 0.015378134267996769 |  |  |  |
| **pneumonia** | 104010138.0 | 0.01430067147449932 | 0.020516096972278422 | 0.025074143974117013 |  |  |  |
| **PH PNA_average** | 104179400.0 | 0.015095476990477558 | 0.06274660135590827 | 0.08502696144513348 |  |  |  |
| **PO2 PNA_average** | 104181846.0 | 0.015901618586103467 | 0.08315408369715689 | 0.09798463197627395 |  |  |  |
| **LAKTAT_average** | 104832485.0 | 0.018279466712618517 | 0.016336107138886086 | 0.015110248114427134 |  |  |  |
| **Positive_tunge_test** | 104279640.0 | 0.0201032681240971 | 0.0049338018670358145 | 0.00700997573469938 |  |  |  |
| **ICD_Y** | 104306232.0 | 0.021168465698354056 | 0.003301032903844106 | 0.0052574818010245345 |  |  |  |
| **urinarytractinfection** | 105275162.5 | 0.022632431523348557 | 0.06978312568771519 | 0.06228093825829065 |  |  |  |
| **Positive_blod_test** | 104441769.5 | 0.023189303091335756 | 0.00017747488730344657 | 0.0006740361283364788 |  |  |  |
| **LAKTAT BLODGASS VENØST** | 104607932.5 | 0.025263014951503338 | 0.0023959109785965287 | 0.0007009975734699381 |  |  |  |
| **LAKTAT** | 104596786.5 | 0.03729786455498676 | 0.0019202782806232918 | 0.0015907252628740902 |  |  |  |
| **procedure_H** | 104549292.0 | 0.046843265692264754 | 0.0006389095942924076 | 0.0 |  |  |  |
| **Positive_biopsi_test** | 104602027.0 | 0.048815044929333695 | 0.0021296986476413587 | 0.0008088433540037746 |  |  |  |
| **procedure_Q_aggregate** | 103377301.5 | 0.05041406474743897 | 1.07517836226174 | 1.0842545160420598 |  |  |  |
| **skinandsofttissueinfection** | 104985557.0 | 0.06432417343655313 | 0.04074823412487133 | 0.03612833647883527 |  |  |  |
| **Positive_eye_test** | 104686549.5 | 0.0711166822854214 | 0.0071699854470592414 | 0.005392289026691831 |  |  |  |
| **ICD_T** | 104118058.0 | 0.08845808524750652 | 0.02679870798282043 | 0.03302777028848746 |  |  |  |
| **procedure_5_aggregate** | 104390684.5 | 0.09510903970403844 | 0.002520143399708941 | 0.0041790239956861685 |  |  |  |
| **LAKTAT PNA** | 104446213.5 | 0.1007463368456161 | 0.00033365278813047954 | 0.001550283095173901 |  |  |  |
| **PO2 PNA** | 104446235.0 | 0.10090076219001072 | 0.003251339935399141 | 0.006362901051496361 |  |  |  |
| **PH PNA** | 104446250.5 | 0.10101220873651749 | 0.002641181272849892 | 0.005931517929361013 |  |  |  |
| **Positive_hud_test** | 104173359.0 | 0.10153556488252885 | 0.024988464132325278 | 0.02615260177945538 |  |  |  |
| **Positive_annet_test** | 104842672.0 | 0.11636063044840872 | 0.034394633159407946 | 0.029387975195470477 |  |  |  |
| **centralnervoussystem** | 104539664.0 | 0.17465592535761387 | 0.0007098995492137863 | 0.00026961445133459155 |  |  |  |
| **ICD_X** | 104535955.0 | 0.20149195848001156 | 0.0006744045717530969 | 0.00026961445133459155 |  |  |  |
| **procedure_I** | 104599916.5 | 0.24847136594611963 | 0.005714691371170979 | 0.004313831221353465 |  |  |  |
| **ICD_T_aggregate** | 103897761.0 | 0.333648089710753 | 0.9484257977496184 | 0.8030466433000809 |  |  |  |
| **procedure_K** | 104682388.5 | 0.3531162826937859 | 0.027579597486955595 | 0.025074143974117013 |  |  |  |
| **procedure_Z** | 104279583.0 | 0.37305717826447526 | 0.03393319845241898 | 0.03626314370450256 |  |  |  |
| **lung** | 104395922.0 | 0.4292301896754306 | 0.00876725943279026 | 0.009301698571043408 |  |  |  |
| **ICD_K** | 104793352.5 | 0.4674863224327619 | 0.10975047030845135 | 0.11957400916689134 |  |  |  |
| **ICD_S** | 104305001.0 | 0.47410818356777207 | 0.043623327299187165 | 0.05230520355891076 |  |  |  |
| **procedure_B** | 104510733.5 | 0.587741174093966 | 0.000567919639371029 | 0.0005392289026691831 |  |  |  |
| **CRP-HØYSENSITIV_average** | 104278146.5 | 0.5954081187949578 | 1.5298287915461108 | 3.1688857896394973 |  |  |  |
| **BILIRUBIN KONJUGERT** | 104524746.5 | 0.5961748344073067 | 0.03514002768608242 | 0.12995416554327313 |  |  |  |
| **Positive_melk_test** | 104405552.5 | 0.5997749612039122 | 0.018457388279558444 | 0.02291722836344028 |  |  |  |
| **ICD_X_aggregate** | 104566435.0 | 0.6298775519508257 | 0.03095162034572108 | 0.0319493124831491 |  |  |  |
| **procedure_D_aggregate** | 104264702.5 | 0.6753327193466161 | 1.080076669151315 | 1.0532488541385818 |  |  |  |
| **ICD_V** | 104507782.0 | 0.6835816332286178 | 0.0006744045717530969 | 0.0005392289026691831 |  |  |  |
| **endocarditis** | 104478898.0 | 0.7580835652481792 | 0.0012068292336634367 | 0.0013480722566729577 |  |  |  |
| **BILIRUBIN UKONJUGERT** | 104504153.0 | 0.8303720964799104 | 0.09210946651048876 | 0.036397950930169856 |  |  |  |
| **Positive_anus_test** | 104461411.0 | 0.8666174357440665 | 0.022858765484683918 | 0.021838770558101916 |  |  |  |
| **procedure_N** | 104467335.0 | 0.9012484926199882 | 0.035707947325453446 | 0.04044216770018873 |  |  |  |
| **ppid** | 104557209.0 | 0.9356647615510758 | 18170.396052958506 | 18159.356834726343 |  |  |  |
| **Positive_bein_test** | 104494406.5 | 0.9630362622199063 | 0.00014197990984275724 | 0.00013480722566729578 |  |  |  |

**Supplementary Table 4. Sorted statistical summary table for 30-day mortality predictors at admission**

|  | **Stat** | **P-Value** | **Mean (Class 0)** | **Mean (Class 1)** | **Chi2 Stat** | **Proportion (Class 0)** | **Proportion (Class 1)** |
| --- | --- | --- | --- | --- | --- | --- | --- |
| **ICD_C** | 71549881.0 | 0.0 | 0.10201256522202108 | 0.6658128875707738 |  |  |  |
| **CRP** | 64524392.5 | 0.0 | 30.335534021935885 | 74.67476633414219 |  |  |  |
| **LEUKOCYTTER** | 74426042.0 | 0.0 | 6.576182751807295 | 10.83327446751144 |  |  |  |
| **ICD_I** | 78565754.5 | 0.0 | 0.20370567564689596 | 0.746157994068482 |  |  |  |
| **ICD_J** | 80707904.0 | 0.0 | 0.14293827423419586 | 0.49015907252628743 |  |  |  |
| **procedure_R_aggregate** | 80991734.0 | 0.0 | 1.203457210804671 | 1.8061472094904287 |  |  |  |
| **ICD_J_aggregate** | 75051088.0 | 0.0 | 2.1058460227877753 | 4.130493394445942 |  |  |  |
| **ICD_I_aggregate** | 70409711.5 | 0.0 | 5.215170553366699 | 8.984631976273928 |  |  |  |
| **age** | 55954082.5 | 0.0 | 55.44148652965605 | 75.13345915341063 |  |  |  |
| **total_los** | 55653342.5 | 0.0 | 29.37551319821564 | 55.32891839669267 |  |  |  |
| **time_to_last** | 139810218.5 | 0.0 | 4029.568381074078 | 1528.2231059584794 |  |  |  |
| **care_level_code** |  | 0.0 |  |  | 5309.82522571323 | 0.7020906541724345 | 0.6952008627662443 |
| **urgency_code** |  | 0.0 |  |  | 3476.8719636480846 | 0.6887445426472154 | 0.6350768401186303 |
| **ICD_C_aggregate** | 66088361.5 | 0.0 | 5.717992404074823 | 21.923159881369642 |  |  |  |
| **procedure_G** | 92445021.5 | 6.461147808177588e-307 | 0.05373939587548362 | 0.2100296575896468 |  |  |  |
| **ICD_D_aggregate** | 81522390.0 | 1.0225342241721986e-248 | 1.3226138501402052 | 2.241978970072796 |  |  |  |
| **KREATININ_average** | 78101941.5 | 1.7937638791577205e-246 | 69.55719831856099 | 96.03102039289094 |  |  |  |
| **BILIRUBIN TOTAL** | 84048749.5 | 2.224428000205524e-239 | 4.395248405683929 | 10.34822953176957 |  |  |  |
| **KREATININ** | 79927593.0 | 9.871251480293905e-217 | 57.850612051728 | 95.90825536982118 |  |  |  |
| **procedure_G_aggregate** | 84138332.5 | 2.5276146830778575e-213 | 1.2656799062932595 | 2.038285252089512 |  |  |  |
| **ICD_E_aggregate** | 83236161.5 | 2.527435546142632e-204 | 2.262733823164022 | 2.9621191695874898 |  |  |  |
| **procedure_U_aggregate** | 84930257.0 | 9.117993254886273e-203 | 0.7292798069073226 | 1.3982205446211917 |  |  |  |
| **prior_comorbidities_counts** | 82260989.5 | 1.0661305606478129e-197 | 0.88620310226103 | 1.2905095713130224 |  |  |  |
| **cancer** | 93554126.5 | 8.975823485430375e-180 | 0.06680154758101729 | 0.18172014019951468 |  |  |  |
| **procedure_R** | 98428871.0 | 3.090305100186988e-166 | 0.015582295105242608 | 0.08344567268805608 |  |  |  |
| **Positive_urin_test** | 85159359.0 | 1.7940187801159525e-161 | 1.4234905760834842 | 2.040442167700189 |  |  |  |
| **procedure_P_aggregate** | 90861981.0 | 9.09574276985113e-142 | 0.33454016256699676 | 0.6221353464545699 |  |  |  |
| **cardiovascular** | 89793234.5 | 1.5143070950098356e-134 | 0.24228871614666525 | 0.3968724723645187 |  |  |  |
| **procedure_W_aggregate** | 86694332.0 | 4.942459850275391e-125 | 3.9896354665814786 | 7.32704232946886 |  |  |  |
| **ICD_N** | 93989762.0 | 1.2466367731610224e-119 | 0.11560714158946508 | 0.23726071717444056 |  |  |  |
| **ICD_E** | 95326940.5 | 6.173932779419837e-118 | 0.08564938061264331 | 0.20490698301428956 |  |  |  |
| **procedure_T_aggregate** | 90388872.0 | 3.177158173687538e-113 | 0.6153764242359706 | 1.0806147209490429 |  |  |  |
| **ICD_N_aggregate** | 88194014.5 | 5.312688476904842e-110 | 3.7141589465090687 | 7.733620922081424 |  |  |  |
| **procedure_P** | 101035132.0 | 1.8426844194823795e-108 | 0.007631420154048202 | 0.05217039633324346 |  |  |  |
| **procedure_Y_aggregate** | 102541509.5 | 8.605980998851064e-105 | 0.0004969296844496504 | 0.028174710164464815 |  |  |  |
| **procedure_Y** | 102736544.5 | 2.316684852241728e-104 | 3.549497746068931e-05 | 0.02386087894311135 |  |  |  |
| **ICD_O_aggregate** | 110650265.0 | 1.5626028601862474e-96 | 0.49135697298832215 | 0.009706120248045295 |  |  |  |
| **procedure_M_aggregate** | 110566746.5 | 8.144818502358003e-96 | 0.46778830795442444 | 0.012671879212725803 |  |  |  |
| **procedure_F_aggregate** | 90623111.5 | 7.971645053399038e-94 | 1.4430838036417848 | 1.916149905634942 |  |  |  |
| **Positive_edta_test** | 96825745.5 | 4.626669279080359e-92 | 0.121215348028254 | 0.2636829334052305 |  |  |  |
| **ICD_A_aggregate** | 92787483.5 | 4.850371328140499e-89 | 0.38441060589926523 | 0.5923429495820977 |  |  |  |
| **ICD_Z** | 93957829.0 | 6.706717631485326e-82 | 0.19075000887374435 | 0.3025074143974117 |  |  |  |
| **Positive_blood_culture_test_test** | 96757959.5 | 1.4437130155803911e-74 | 0.11180917900117134 | 0.22661094634672418 |  |  |  |
| **total_ICU_LOS** | 90616578.5 | 9.549015002490682e-74 | 1.8076231379926433 | 2.9272321829783507 |  |  |  |
| **ICD_A** | 99770196.5 | 1.52009926562003e-71 | 0.03041919568381074 | 0.0764356969533567 |  |  |  |
| **ICD_D** | 99455368.0 | 5.680102566866028e-67 | 0.03911546516167962 | 0.09989215421946616 |  |  |  |
| **PH_average** | 110946234.0 | 2.497893010108104e-65 | 0.711623004791627 | 0.2570426635391474 |  |  |  |
| **procedure_T** | 102468272.0 | 1.458061868973886e-62 | 0.004188407340361339 | 0.025208951199784307 |  |  |  |
| **Positive_hal_test** | 99488660.0 | 3.2377610744579957e-62 | 0.05040286799417883 | 0.11674305742787813 |  |  |  |
| **ICD_P_aggregate** | 109021797.0 | 6.802837309796926e-62 | 0.3350725872289071 | 0.04286869776220006 |  |  |  |
| **organdysfunction** | 97949570.0 | 1.6610888574467235e-57 | 0.09239342633017428 | 0.15772445403073604 |  |  |  |
| **ICD_Z_aggregate** | 92240451.5 | 1.6101120291431334e-55 | 8.39953146629752 | 13.943246157994068 |  |  |  |
| **LAKTAT BLODGASS_average** | 108309454.0 | 2.352992528384936e-52 | 0.08016756706157388 | 0.014720721913844152 |  |  |  |
| **Positive_plasma_test** | 98160723.5 | 1.1178466511556898e-51 | 0.15003726972633372 | 0.26934483688325694 |  |  |  |
| **ICD_M** | 109881934.0 | 1.711999836259085e-51 | 0.09125758705143222 | 0.03531949312483149 |  |  |  |
| **ICD_R_aggregate** | 92940110.0 | 2.996441892051741e-51 | 2.7780854009157703 | 2.8854138581827984 |  |  |  |
| **ICD_H** | 108544372.5 | 1.7543104142969675e-49 | 0.05530117488375395 | 0.011863035858722027 |  |  |  |
| **ICD_F** | 100167796.0 | 6.506232912619054e-47 | 0.048805594008447806 | 0.09355621461310326 |  |  |  |
| **CRP_average** | 93391150.5 | 3.456787092532515e-45 | 54.92968674355863 | 60.5767158248939 |  |  |  |
| **PH** | 107810685.5 | 2.0896554961794823e-44 | 0.27922538127521584 | 0.04523860878943112 |  |  |  |
| **TROMBOCYTTER** | 94768752.5 | 5.035339015328122e-38 | 140.32223878654509 | 163.23831670710885 |  |  |  |
| **procedure_X_aggregate** | 101497771.5 | 3.882770842424184e-37 | 0.02903489156284386 | 0.0637638177406309 |  |  |  |
| **infection** | 96302059.5 | 4.3075193822821885e-35 | 0.396549888190821 | 0.49770827716365595 |  |  |  |
| **Positive_bronki_test** | 102283452.5 | 7.178507778505122e-34 | 0.015724275015085366 | 0.04017255324885414 |  |  |  |
| **ICD_Q_aggregate** | 108181231.0 | 2.187826908231105e-32 | 0.4477691406665957 | 0.1275276354812618 |  |  |  |
| **dementia** | 102049888.0 | 1.430375398988515e-31 | 0.019912682355446704 | 0.04367754111620383 |  |  |  |
| **BILIRUBIN TOTAL_average** | 95565379.5 | 3.690748862302253e-30 | 13.487312738558972 | 12.612933964125258 |  |  |  |
| **procedure_D** | 106844171.0 | 1.1024833607018874e-28 | 0.04319738756965889 | 0.009301698571043408 |  |  |  |
| **procedure_C** | 106377213.5 | 3.708607221870702e-28 | 0.027047172825045256 | 0.0018873011593421407 |  |  |  |
| **ICD_F_aggregate** | 98508343.5 | 6.300614687658106e-26 | 3.6310297092961346 | 2.371528713939067 |  |  |  |
| **ICD_K_aggregate** | 96903020.5 | 3.4506788715844646e-25 | 2.7748908529443086 | 2.577514154758695 |  |  |  |
| **procedure_U** | 103359168.5 | 1.0006194954820554e-23 | 0.004756326979732368 | 0.016985710434079267 |  |  |  |
| **procedure_O** | 106600839.5 | 1.0802149080806325e-23 | 0.039186455116601 | 0.010245349150714478 |  |  |  |
| **ICD_O** | 105716330.0 | 4.987008878310634e-20 | 0.020232137152592908 | 0.0004044216770018873 |  |  |  |
| **procedure_L_aggregate** | 108312258.5 | 3.421784574536348e-19 | 0.3592446668796365 | 0.20463736856295497 |  |  |  |
| **ICD_R** | 100540188.0 | 5.245791812563757e-19 | 0.12011500372697263 | 0.16486923699110273 |  |  |  |
| **CRP-HØYSENSITIV** | 105832562.5 | 1.288711929787807e-18 | 0.11413161537642413 | 0.187502022108385 |  |  |  |
| **LAKTAT BLODGASS VENØST_average** | 105644995.5 | 6.1576740822351074e-18 | 0.02119061140943795 | 0.0038532398669902036 |  |  |  |
| **Positive_naso_test** | 101077106.5 | 1.700408356522734e-17 | 0.11716892059773543 | 0.16122944189808575 |  |  |  |
| **procedure_M** | 105483960.0 | 3.89391677880088e-17 | 0.016185709722074327 | 0.0 |  |  |  |
| **procedure_W** | 101404125.0 | 2.4533262939378747e-16 | 0.08607532034217158 | 0.1365597196009706 |  |  |  |
| **procedure_Z_aggregate** | 110298205.0 | 3.5800802194477833e-16 | 3.829872573030916 | 2.597600431383122 |  |  |  |
| **Gender** |  | 3.3766183934485417e-15 |  |  | 62.033511631887876 | 0.5139672736307812 | 0.5653815044486384 |
| **ICD_L** | 106464572.5 | 1.7570879969753303e-14 | 0.044617186668086464 | 0.023321650040442166 |  |  |  |
| **ICD_B_aggregate** | 100296963.5 | 1.3652839047064898e-13 | 0.5155290526390516 | 0.5246697222971152 |  |  |  |
| **procedure_K_aggregate** | 100408301.0 | 2.1931128269290067e-13 | 1.5470485926241437 | 4.92922620652467 |  |  |  |
| **ICD_H_aggregate** | 100033294.5 | 8.440886746405153e-13 | 1.9927590245980193 | 2.0548665408465894 |  |  |  |
| **TROMBOCYTTER_average** | 110046734.0 | 1.7144484165889526e-12 | 235.96674330079503 | 229.28658489007347 |  |  |  |
| **ICD_W_aggregate** | 102595212.0 | 1.8070273855213364e-12 | 0.041529123629006494 | 0.06457266109463468 |  |  |  |
| **ICD_Y_aggregate** | 102294515.5 | 1.0684703916042478e-11 | 0.07024456039470416 | 0.09112968455109194 |  |  |  |
| **ICD_M_aggregate** | 109235060.5 | 1.5408889040072222e-11 | 3.5540056082064386 | 2.1884605014828793 |  |  |  |
| **sepsis** | 103765887.0 | 1.8332224667866904e-11 | 0.004898306889575125 | 0.011863035858722027 |  |  |  |
| **procedure_L** | 105175953.0 | 5.234422414085725e-11 | 0.010471018350903347 | 0.0012132650310056618 |  |  |  |
| **LAKTAT BLODGASS** | 105309819.5 | 2.4410928420978166e-10 | 0.01990972444065831 | 0.009146670261526015 |  |  |  |
| **procedure_O_aggregate** | 108276514.0 | 2.19729290443262e-09 | 3.365704752777482 | 2.3685629549743865 |  |  |  |
| **procedure_F** | 102957228.5 | 5.169091803468203e-09 | 0.04131615376424236 | 0.07400916689134537 |  |  |  |
| **ICD_G** | 102650295.0 | 6.364643323736832e-09 | 0.05583359954566429 | 0.07764896198436236 |  |  |  |
| **LEUKOCYTTER_average** | 99950989.0 | 7.905770560694441e-09 | 8.751987631339986 | 9.278849674972538 |  |  |  |
| **procedure_I_aggregate** | 106982345.5 | 1.5476062983633245e-08 | 1.6968373975082527 | 0.9770827716365598 |  |  |  |
| **procedure_A** | 103420001.5 | 2.9039856170592678e-08 | 0.028147517126326623 | 0.040711782151523324 |  |  |  |
| **procedure_C_aggregate** | 101684380.5 | 7.145732828447206e-08 | 1.0571469137117098 | 1.059719600970612 |  |  |  |
| **procedure_E** | 104999648.0 | 7.37792037756632e-08 | 0.00724097540198062 | 0.0010784578053383662 |  |  |  |
| **procedure_B_aggregate** | 105417300.5 | 3.5983887950573667e-07 | 0.034785077911475525 | 0.016042059854408198 |  |  |  |
| **procedure_J** | 106061536.5 | 5.209436631655601e-07 | 0.07123841976360346 | 0.06848207063898624 |  |  |  |
| **ICD_G_aggregate** | 101544903.5 | 8.105848268610126e-07 | 1.64366592127214 | 1.4228902669183068 |  |  |  |
| **explicitsepsis** | 104208325.0 | 1.3854254924359366e-06 | 0.0013843041209668833 | 0.004044216770018873 |  |  |  |
| **ICD_Q** | 105217534.5 | 1.7769583708007634e-06 | 0.01448195080396124 | 0.009301698571043408 |  |  |  |
| **Positive_ear_test** | 105050587.5 | 7.654135066836614e-06 | 0.013665566322365385 | 0.006605554057697492 |  |  |  |
| **procedure_E_aggregate** | 105927272.0 | 9.63951782672537e-06 | 0.11141873424910376 | 0.08708546778107307 |  |  |  |
| **ICD_U** | 104190510.5 | 1.072528293856268e-05 | 0.001952223760337912 | 0.004987867349689944 |  |  |  |
| **ICD_U_aggregate** | 103843306.5 | 2.322706947488587e-05 | 0.02541440386185355 | 0.06484227554596926 |  |  |  |
| **procedure_Q** | 105256300.0 | 3.257405883344266e-05 | 0.023994604763425974 | 0.01712051765974656 |  |  |  |
| **ICD_P** | 105030545.5 | 3.3008008191774997e-05 | 0.01803144855003017 | 0.019681854947425182 |  |  |  |
| **LAKTAT PNA** | 104297841.5 | 9.12506704269565e-05 | 0.0009122209207397154 | 0.004650849285521705 |  |  |  |
| **PH PNA** | 104298059.5 | 9.291138991212599e-05 | 0.007130940971852482 | 0.020942302507414398 |  |  |  |
| **PO2 PNA** | 104298089.0 | 9.31383026386289e-05 | 0.009512653959464734 | 0.02310595847937449 |  |  |  |
| **ICD_S_aggregate** | 101948491.0 | 9.759260890999675e-05 | 1.0059631562133957 | 0.9421677001887301 |  |  |  |
| **procedure_A_aggregate** | 106855752.5 | 0.00010726486312645504 | 0.9360380506158379 | 0.8627662442706929 |  |  |  |
| **LAKTAT BLODGASS VENØST** | 104725861.0 | 0.00024747469371452525 | 0.004467042913427751 | 0.002709625235912645 |  |  |  |
| **ICD_V_aggregate** | 104926858.0 | 0.0006810252641772957 | 0.019948177332907394 | 0.021838770558101916 |  |  |  |
| **procedure_H_aggregate** | 105171323.0 | 0.0007212888704293516 | 0.043126397614737515 | 0.02480452952278242 |  |  |  |
| **ICD_B** | 103712851.5 | 0.0018690745578963171 | 0.035211017641003796 | 0.04543003504987867 |  |  |  |
| **procedure_J_aggregate** | 102456777.5 | 0.0027657524115532192 | 1.3723068185851701 | 1.5163116743057428 |  |  |  |
| **procedure_X** | 104434351.5 | 0.003718840069792487 | 0.00010648493238206793 | 0.0006740361283364788 |  |  |  |
| **procedure_N_aggregate** | 106353592.5 | 0.004506992072759957 | 1.197990984275725 | 0.9684551091938528 |  |  |  |
| **ICD_L_aggregate** | 102845052.5 | 0.005577842351054158 | 1.6548113441947965 | 1.4595578322998113 |  |  |  |
| **PO2_average** | 104465484.0 | 0.00585046974121691 | 0.0 | 0.002709625235912645 |  |  |  |
| **Positive_tunge_test** | 104249969.0 | 0.006651533235239747 | 0.004614347069889611 | 0.00700997573469938 |  |  |  |
| **intraabdominalinfection** | 104605676.5 | 0.008772158504936182 | 0.0012068292336634367 | 0.00013480722566729578 |  |  |  |
| **LAKTAT_average** | 104859529.0 | 0.009680545460867954 | 0.016022335639444014 | 0.014193558979889524 |  |  |  |
| **ICD_W** | 104328414.5 | 0.013045389208181941 | 0.00205870869271998 | 0.0037746023186842814 |  |  |  |
| **pneumonia** | 104010138.0 | 0.01430067147449932 | 0.020516096972278422 | 0.025074143974117013 |  |  |  |
| **LAKTAT PNA_average** | 104184430.5 | 0.01629405722340584 | 0.011095729954211483 | 0.015175923429495825 |  |  |  |
| **PH PNA_average** | 104189654.0 | 0.018372021599011575 | 0.06248145387427692 | 0.08403882448099219 |  |  |  |
| **PO2 PNA_average** | 104192080.0 | 0.019324253927759633 | 0.08276718844283537 | 0.09737799946077112 |  |  |  |
| **ICD_Y** | 104306232.0 | 0.021168465698354056 | 0.003301032903844106 | 0.0052574818010245345 |  |  |  |
| **urinarytractinfection** | 105275162.5 | 0.022632431523348557 | 0.06978312568771519 | 0.06228093825829065 |  |  |  |
| **Positive_led_test** | 104637665.0 | 0.02762038785865171 | 0.004259397295282717 | 0.0016176867080075492 |  |  |  |
| **Positive_annet_test** | 104948116.5 | 0.03513862697818076 | 0.03247790437653072 | 0.026557023456457267 |  |  |  |
| **procedure_H** | 104549292.0 | 0.046843265692264754 | 0.0006389095942924076 | 0.0 |  |  |  |
| **procedure_Q_aggregate** | 103377301.5 | 0.05041406474743897 | 1.07517836226174 | 1.0842545160420598 |  |  |  |
| **PO2** | 104479570.5 | 0.0513244353821535 | 0.0 | 0.0014289565920733351 |  |  |  |
| **Positive_biopsi_test** | 104594609.0 | 0.06179343151308095 | 0.00205870869271998 | 0.0008088433540037746 |  |  |  |
| **skinandsofttissueinfection** | 104985557.0 | 0.06432417343655313 | 0.04074823412487133 | 0.03612833647883527 |  |  |  |
| **BILIRUBIN UKONJUGERT_average** | 104194926.5 | 0.07129469009298285 | 0.6289149666851721 | 0.4133054731733621 |  |  |  |
| **ICD_T** | 104118058.0 | 0.08845808524750652 | 0.02679870798282043 | 0.03302777028848746 |  |  |  |
| **LAKTAT** | 104573063.5 | 0.13822830903574057 | 0.0021722926205941864 | 0.0024534915071447827 |  |  |  |
| **Positive_eye_test** | 104645015.5 | 0.14148421015123047 | 0.006602065807688212 | 0.0052574818010245345 |  |  |  |
| **BILIRUBIN KONJUGERT_average** | 104239162.0 | 0.1678381341728712 | 0.6198953063051666 | 1.1386739462568525 |  |  |  |
| **Positive_anus_test** | 104744105.5 | 0.17244461147737822 | 0.021438966386256343 | 0.01725532488541386 |  |  |  |
| **centralnervoussystem** | 104539664.0 | 0.17465592535761387 | 0.0007098995492137863 | 0.00026961445133459155 |  |  |  |
| **BILIRUBIN KONJUGERT** | 104575173.5 | 0.17726409108466723 | 0.05327796116849466 | 0.10380156376381774 |  |  |  |
| **Positive_faeces_test** | 104200150.0 | 0.1855660250464709 | 0.03624037198736379 | 0.03585872202750067 |  |  |  |
| **ICD_X** | 104535955.0 | 0.20149195848001156 | 0.0006744045717530969 | 0.00026961445133459155 |  |  |  |
| **Positive_hud_test** | 104271226.5 | 0.2378165354338586 | 0.023391190146594257 | 0.023726071717444053 |  |  |  |
| **Positive_blod_test** | 104469942.5 | 0.24603295073416176 | 0.00017747488730344657 | 0.0004044216770018873 |  |  |  |
| **procedure_I** | 104599916.5 | 0.24847136594611963 | 0.005714691371170979 | 0.004313831221353465 |  |  |  |
| **BILIRUBIN UKONJUGERT** | 104544197.5 | 0.33188717359774134 | 0.09058318247967913 | 0.02601779455378808 |  |  |  |
| **ICD_T_aggregate** | 103897761.0 | 0.333648089710753 | 0.9484257977496184 | 0.8030466433000809 |  |  |  |
| **procedure_K** | 104682388.5 | 0.3531162826937859 | 0.027579597486955595 | 0.025074143974117013 |  |  |  |
| **procedure_Z** | 104279583.0 | 0.37305717826447526 | 0.03393319845241898 | 0.03626314370450256 |  |  |  |
| **lung** | 104395922.0 | 0.4292301896754306 | 0.00876725943279026 | 0.009301698571043408 |  |  |  |
| **ICD_K** | 104793352.5 | 0.4674863224327619 | 0.10975047030845135 | 0.11957400916689134 |  |  |  |
| **ICD_S** | 104305001.0 | 0.47410818356777207 | 0.043623327299187165 | 0.05230520355891076 |  |  |  |
| **Positive_melk_test** | 104588050.0 | 0.5587532011889409 | 0.017392538955737762 | 0.019681854947425182 |  |  |  |
| **procedure_B** | 104510733.5 | 0.587741174093966 | 0.000567919639371029 | 0.0005392289026691831 |  |  |  |
| **ICD_X_aggregate** | 104566435.0 | 0.6298775519508257 | 0.03095162034572108 | 0.0319493124831491 |  |  |  |
| **procedure_D_aggregate** | 104264702.5 | 0.6753327193466161 | 1.080076669151315 | 1.0532488541385818 |  |  |  |
| **ICD_V** | 104507782.0 | 0.6835816332286178 | 0.0006744045717530969 | 0.0005392289026691831 |  |  |  |
| **CRP-HØYSENSITIV_average** | 104644460.0 | 0.7023105934003187 | 1.4005240259616638 | 2.4888569392883575 |  |  |  |
| **endocarditis** | 104478898.0 | 0.7580835652481792 | 0.0012068292336634367 | 0.0013480722566729577 |  |  |  |
| **procedure_N** | 104467335.0 | 0.9012484926199882 | 0.035707947325453446 | 0.04044216770018873 |  |  |  |
| **ppid** | 104557209.0 | 0.9356647615510758 | 18170.396052958506 | 18159.356834726343 |  |  |  |
| **Positive_bein_test** | 104494406.5 | 0.9630362622199063 | 0.00014197990984275724 | 0.00013480722566729578 |  |  |  |

**Supplementary Table 5. Statistical summary table for readmission predictors.**

|  | **Stat** | **P-Value** | **Mean (Class 0)** | **Mean (Class 1)** | **Chi2 Stat** | **Proportion (Class 0)** | **Proportion (Class 1)** |
| --- | --- | --- | --- | --- | --- | --- | --- |
| **urgency_code** |  | 0.0 |  |  | 1495.6132847379947 | 0.6807283763277694 | 0.49494609164420483 |
| **care_level_code** |  | 0.0 |  |  | 1675.9580309925614 | 0.6700050581689428 | 0.503032345013477 |
| **readmission** | 73300270.0 | 1.5617851875270433e-259 | 0.10706457595683695 | 0.2742587601078167 |  |  |  |
| **CRP** | 64015954.5 | 1.3346958372170159e-253 | 24.322300904850223 | 52.30337488769092 |  |  |  |
| **LOS** | 65080854.5 | 2.7310169368787146e-250 | 1.5301312313831048 | 2.154537286612745 |  |  |  |
| **age** | 64884344.0 | 5.841356260310446e-225 | 55.242454897993596 | 67.17553908355795 |  |  |  |
| **ICD_C** | 73912389.5 | 2.718255576854275e-215 | 0.15420671050413085 | 0.37331536388140163 |  |  |  |
| **ICD_C_aggregate** | 71184081.5 | 5.6036103992561625e-192 | 7.42400944191536 | 16.129885444743934 |  |  |  |
| **LEUKOCYTTER** | 68683534.0 | 9.318289074767002e-162 | 5.750231607935724 | 8.497265554806827 |  |  |  |
| **total_los** | 70764668.0 | 5.3713693944255296e-126 | 30.803217557466315 | 42.78442273135668 |  |  |  |
| **ICD_I_aggregate** | 71841426.0 | 2.090376144569754e-124 | 5.235238576968471 | 7.926212938005391 |  |  |  |
| **time_to_last** | 104469491.5 | 7.37950298752333e-115 | 2926.7478334176362 | 2177.162398921833 |  |  |  |
| **ICD_J** | 79396887.0 | 1.0660688098700882e-102 | 0.12409374473107401 | 0.26398247978436656 |  |  |  |
| **procedure_0_aggregate** | 82334880.0 | 3.569048563069573e-101 | 0.3897824987354578 | 1.3396226415094339 |  |  |  |
| **ICD_I** | 78510831.0 | 2.7391505957388863e-92 | 0.22094081942336874 | 0.44845013477088946 |  |  |  |
| **procedure_R_aggregate** | 78542812.5 | 2.840466792866408e-88 | 1.2972854493340078 | 1.3096361185983827 |  |  |  |
| **KREATININ_average** | 73650858.0 | 3.732200210925262e-88 | 67.64950820517976 | 83.89065381999715 |  |  |  |
| **ICD_J_aggregate** | 76406340.0 | 9.980969169546653e-69 | 2.094587759231158 | 3.401280323450135 |  |  |  |
| **prior_comorbidities_counts** | 76643264.0 | 5.989805463824227e-64 | 0.8601584892935424 | 1.1159029649595686 |  |  |  |
| **ICD_D_aggregate** | 77890238.5 | 2.601211618606366e-60 | 1.360816051256112 | 1.9701819407008085 |  |  |  |
| **ICD_E_aggregate** | 77796466.5 | 1.2492676441250163e-59 | 2.2152419490810993 | 2.711421832884097 |  |  |  |
| **KREATININ** | 76496210.0 | 2.256786006386439e-59 | 52.82314058899562 | 71.55912314690026 |  |  |  |
| **cancer** | 82571646.0 | 4.603968907920082e-55 | 0.07752486933063564 | 0.14487870619946092 |  |  |  |
| **BILIRUBIN TOTAL** | 80492324.0 | 3.238969065590062e-53 | 4.107008374079694 | 6.576819407008086 |  |  |  |
| **cardiovascular** | 79751986.0 | 4.767010405249556e-53 | 0.24535491485415614 | 0.3507412398921833 |  |  |  |
| **Positive_urin_test** | 78606541.0 | 1.4163923017114096e-47 | 1.393019726858877 | 1.7946428571428572 |  |  |  |
| **procedure_U_aggregate** | 79907768.0 | 4.255295383284669e-43 | 0.8004046535154274 | 1.1661051212938005 |  |  |  |
| **CRP_average** | 78219377.0 | 5.63306620682026e-42 | 48.71917617114914 | 56.517417597312715 |  |  |  |
| **ICD_N_aggregate** | 79394841.5 | 5.5224086480021174e-39 | 4.225324565840499 | 5.3382749326145555 |  |  |  |
| **procedure_P_aggregate** | 82215168.0 | 5.122221292510266e-33 | 0.3509357612544259 | 0.5106132075471698 |  |  |  |
| **procedure_R** | 85772847.5 | 8.033711873933326e-33 | 0.020367560276513236 | 0.049528301886792456 |  |  |  |
| **procedure_W_aggregate** | 80167327.5 | 8.52787354188764e-31 | 4.295026133872871 | 6.055424528301887 |  |  |  |
| **ICD_N** | 83911042.0 | 6.278183612780519e-27 | 0.10655875906255269 | 0.16088274932614555 |  |  |  |
| **Positive_edta_test** | 84359958.0 | 1.1093842645937956e-26 | 0.1314112291350531 | 0.22287735849056603 |  |  |  |
| **LEUKOCYTTER_average** | 80499175.0 | 2.2045089545030145e-25 | 8.013050858213386 | 8.927622029236018 |  |  |  |
| **procedure_G_aggregate** | 81996046.0 | 7.355192251622003e-25 | 1.2796493002866296 | 1.6438679245283019 |  |  |  |
| **total_ICU_LOS** | 80942884.5 | 3.033439717215107e-24 | 1.8688276288427987 | 2.3831143306379077 |  |  |  |
| **ICD_H** | 90677588.5 | 5.501753046529051e-23 | 0.05823638509526218 | 0.02543800539083558 |  |  |  |
| **ICU_LOS** | 81336199.0 | 3.3942353360533694e-22 | 0.7634055527454642 | 1.075584007187786 |  |  |  |
| **Positive_hal_test** | 85406185.5 | 8.311769372805821e-22 | 0.05489799359298601 | 0.09838274932614555 |  |  |  |
| **procedure_T_aggregate** | 82642037.0 | 3.696210704360028e-21 | 0.6612712864609678 | 0.9161051212938005 |  |  |  |
| **ICD_P_aggregate** | 90209478.5 | 1.7705250796483557e-20 | 0.29549822964087 | 0.057446091644204854 |  |  |  |
| **TROMBOCYTTER** | 82050085.5 | 1.2642240927501483e-19 | 112.9341623110212 | 127.3829655211141 |  |  |  |
| **procedure_F_aggregate** | 82472784.0 | 1.6993092124089638e-19 | 1.4435339740347328 | 1.7434299191374663 |  |  |  |
| **LAKTAT BLODGASS_average** | 90072910.0 | 2.6706788898267067e-19 | 0.07304264705332761 | 0.030073275607481874 |  |  |  |
| **ICD_Z_aggregate** | 81625740.5 | 4.042991288568356e-19 | 9.001180239419996 | 11.038241239892184 |  |  |  |
| **ICD_M** | 90951164.0 | 5.375035891741112e-19 | 0.08679817905918058 | 0.05053908355795148 |  |  |  |
| **PH_average** | 90946531.0 | 2.9884300676044526e-18 | 0.6176578742451743 | 0.3715277739288312 |  |  |  |
| **procedure_G** | 85919758.0 | 3.315186684805863e-17 | 0.057561962569549825 | 0.08473719676549865 |  |  |  |
| **ICD_A_aggregate** | 83717271.0 | 5.675587630959596e-17 | 0.3704940145000843 | 0.4738881401617251 |  |  |  |
| **infection** | 83057230.0 | 7.3560497479346e-17 | 0.3709323891417973 | 0.4467654986522911 |  |  |  |
| **organdysfunction** | 85115494.0 | 1.1848643025836355e-15 | 0.09158657899173832 | 0.12786388140161725 |  |  |  |
| **ICD_R_aggregate** | 82445780.5 | 1.7140210826138334e-15 | 2.631495531950767 | 2.8684299191374665 |  |  |  |
| **ICD_E** | 85382940.5 | 2.5319967542741406e-15 | 0.0830888551677626 | 0.12415768194070081 |  |  |  |
| **procedure_O** | 89664393.5 | 5.163361719300281e-15 | 0.044916540212443096 | 0.015835579514824796 |  |  |  |
| **procedure_C** | 89293672.5 | 2.6944172761936432e-14 | 0.027617602427921092 | 0.007917789757412398 |  |  |  |
| **ICD_Q_aggregate** | 90018782.5 | 4.3344342892915696e-13 | 0.40283257460799193 | 0.19171159029649595 |  |  |  |
| **Positive_blood_culture_test_test** | 85359079.0 | 1.197280061258437e-12 | 0.11718091384252234 | 0.16795822102425875 |  |  |  |
| **procedure_D** | 89436196.5 | 1.2008087597879019e-12 | 0.04602933737986849 | 0.016340970350404313 |  |  |  |
| **Positive_plasma_test** | 85332056.0 | 1.4753815123662047e-12 | 0.16017534985668522 | 0.21816037735849056 |  |  |  |
| **BILIRUBIN TOTAL_average** | 83087163.0 | 5.276509128431108e-12 | 12.760131229466372 | 11.952399972421341 |  |  |  |
| **ICD_D** | 86276204.0 | 2.2501592832792343e-11 | 0.044545607823301296 | 0.06856469002695417 |  |  |  |
| **ICD_A** | 86716973.0 | 1.1210599359840189e-10 | 0.024919912325071656 | 0.039588948787062 |  |  |  |
| **Gender** |  | 1.3232848857261289e-10 |  |  | 41.27383347983316 | 0.5050075872534142 | 0.5407681940700808 |
| **procedure_J** | 89791491.5 | 2.5104815996225166e-10 | 0.07317484403979092 | 0.04902291105121294 |  |  |  |
| **ICD_O** | 87194609.0 | 2.6056304721421826e-10 | 0.015646602596526723 | 0.02526954177897574 |  |  |  |
| **dementia** | 86931745.0 | 5.480890761370566e-10 | 0.01834429269937616 | 0.03116576819407008 |  |  |  |
| **procedure_P** | 87289003.0 | 7.106615189332912e-10 | 0.009205867475973697 | 0.021731805929919138 |  |  |  |
| **procedure_0** | 87829231.0 | 7.910368308516164e-10 | 0.0002360478839993256 | 0.0023584905660377358 |  |  |  |
| **ICD_F_aggregate** | 84946526.5 | 1.510781523543353e-09 | 3.370257966616085 | 3.0112870619946093 |  |  |  |
| **ICD_B_aggregate** | 85056581.5 | 3.153549686478206e-09 | 0.4675771370763783 | 0.542621293800539 |  |  |  |
| **CRP-HØYSENSITIV** | 88850395.0 | 6.044930220046778e-09 | 0.14437953127634445 | 0.26448618598382745 |  |  |  |
| **procedure_O_aggregate** | 91334139.5 | 8.485669963711247e-09 | 3.240870005058169 | 2.5444743935309972 |  |  |  |
| **ICD_F** | 86612899.0 | 3.042268460629475e-08 | 0.046231664137582194 | 0.06283692722371968 |  |  |  |
| **ICD_K_aggregate** | 84463034.5 | 8.947591688023902e-08 | 2.6250547968302143 | 2.606469002695418 |  |  |  |
| **procedure_A_aggregate** | 90787366.5 | 5.315475851500062e-07 | 0.9134041476985332 | 0.7730795148247979 |  |  |  |
| **procedure_X_aggregate** | 86946140.0 | 6.617840403864482e-07 | 0.033586241780475465 | 0.04834905660377359 |  |  |  |
| **ICD_S** | 86823192.0 | 6.829974207044328e-07 | 0.040161861406170965 | 0.05929919137466307 |  |  |  |
| **procedure_M** | 87459659.0 | 1.3596108131835067e-06 | 0.011970999831394368 | 0.019036388140161724 |  |  |  |
| **procedure_I** | 88488204.0 | 2.62524404434716e-06 | 0.009846568875400438 | 0.0021900269541778976 |  |  |  |
| **procedure_Z** | 89073300.5 | 3.227614742516566e-06 | 0.039217669870173666 | 0.02543800539083558 |  |  |  |
| **ICD_Y_aggregate** | 86714585.0 | 7.011796439142003e-06 | 0.068049232844377 | 0.08507412398921833 |  |  |  |
| **procedure_Z_aggregate** | 90913497.5 | 8.116798941032414e-06 | 3.6532456584049906 | 2.9659703504043127 |  |  |  |
| **Positive_naso_test** | 86439378.5 | 1.2541324555769335e-05 | 0.11832743213623335 | 0.13611859838274934 |  |  |  |
| **Positive_bronki_test** | 87293160.0 | 1.469610037064528e-05 | 0.019119878603945373 | 0.028975741239892182 |  |  |  |
| **procedure_K_aggregate** | 85885606.0 | 2.3938806322046004e-05 | 2.0813690777271963 | 2.942048517520216 |  |  |  |
| **ICD_W** | 87799676.0 | 0.00010684087121850253 | 0.0015848929354240432 | 0.004043126684636119 |  |  |  |
| **procedure_I_aggregate** | 89452041.5 | 0.00034270837855681984 | 1.6148035744393863 | 1.1746967654986522 |  |  |  |
| **ICD_M_aggregate** | 90298472.5 | 0.000353595907986693 | 3.308919237902546 | 2.594676549865229 |  |  |  |
| **ICD_U_aggregate** | 87541042.0 | 0.00037567892296961735 | 0.029539706626201315 | 0.038578167115902964 |  |  |  |
| **procedure_B_aggregate** | 88593390.5 | 0.00047783332652764237 | 0.03277693474962064 | 0.018025606469002694 |  |  |  |
| **ICD_W_aggregate** | 87190105.5 | 0.0006034058608635023 | 0.04242117686730737 | 0.051381401617250674 |  |  |  |
| **LAKTAT BLODGASS VENØST_average** | 88422123.0 | 0.0006228011346631226 | 0.017864007514993854 | 0.010455694070080864 |  |  |  |
| **procedure_Q** | 88615094.0 | 0.0006629914839629783 | 0.024785027819929185 | 0.018025606469002694 |  |  |  |
| **ICD_Q** | 88449669.5 | 0.0010730238440882854 | 0.012780306862249199 | 0.00774932614555256 |  |  |  |
| **procedure_U** | 87613351.5 | 0.0011114678505941098 | 0.009644242117686731 | 0.014487870619946091 |  |  |  |
| **PH** | 88671386.0 | 0.001127274120110212 | 0.20492120496824565 | 0.15018222147349505 |  |  |  |
| **procedure_A** | 88583457.5 | 0.0012613864901442552 | 0.02977575451020064 | 0.018194070080862535 |  |  |  |
| **procedure_T** | 87651396.0 | 0.0016861443302171 | 0.008430281571404486 | 0.013477088948787063 |  |  |  |
| **procedure_L** | 88312928.5 | 0.0018573187647902528 | 0.00900354071825999 | 0.004380053908355795 |  |  |  |
| **procedure_H_aggregate** | 88580664.0 | 0.0020215291461093566 | 0.04130837969988198 | 0.02628032345013477 |  |  |  |
| **ICD_L** | 88696176.5 | 0.002808841351225713 | 0.039318833249030516 | 0.03099730458221024 |  |  |  |
| **sepsis** | 87743571.5 | 0.0028113573760507183 | 0.0048221210588433655 | 0.007917789757412398 |  |  |  |
| **ICD_S_aggregate** | 86254079.0 | 0.0028365998569720263 | 0.9551846231664137 | 0.9075134770889488 |  |  |  |
| **ICD_T** | 87452418.0 | 0.003808081747193096 | 0.02508851795649975 | 0.032513477088948785 |  |  |  |
| **pneumonia** | 87522314.5 | 0.004248283097941105 | 0.018546619457089866 | 0.024090296495956873 |  |  |  |
| **ICD_H_aggregate** | 86422913.5 | 0.004954625859284079 | 1.953667172483561 | 1.9883760107816713 |  |  |  |
| **LAKTAT_average** | 88347935.0 | 0.009492872293577153 | 0.015885666753068347 | 0.01136137316561845 |  |  |  |
| **LAKTAT BLODGASS** | 88323462.0 | 0.009926726518562285 | 0.01832799415500477 | 0.013569743935309972 |  |  |  |
| **procedure_Y_aggregate** | 88102112.0 | 0.015940804248609813 | 0.0015174506828528073 | 0.0 |  |  |  |
| **procedure_N_aggregate** | 89444905.0 | 0.016097341805435362 | 1.1414938458944528 | 0.9733827493261455 |  |  |  |
| **ICD_G_aggregate** | 86749262.5 | 0.018912948519713653 | 1.5591637160681167 | 1.4278975741239892 |  |  |  |
| **procedure_X** | 87971567.5 | 0.02542722256533641 | 0.0001686056314280897 | 0.0008423180592991914 |  |  |  |
| **Positive_ear_test** | 88253733.0 | 0.03279185027354361 | 0.012409374473107401 | 0.008254716981132075 |  |  |  |
| **procedure_M_aggregate** | 88577045.0 | 0.03481765569395891 | 0.37356263699207554 | 0.2929582210242588 |  |  |  |
| **ICD_U** | 87897557.5 | 0.03767811542401563 | 0.0018546619457089867 | 0.003200808625336927 |  |  |  |
| **LAKTAT PNA_average** | 87785714.5 | 0.03792048948039781 | 0.010418984994098799 | 0.011455525606469003 |  |  |  |
| **LAKTAT** | 88113985.5 | 0.03855685648993637 | 0.0019996627887371443 | 0.0012466307277628032 |  |  |  |
| **PH PNA_average** | 87787742.0 | 0.03999197743068937 | 0.055837632776934855 | 0.07513055929919137 |  |  |  |
| **PO2 PNA_average** | 87788501.0 | 0.04065774251199606 | 0.07255774742876414 | 0.09417115902964962 |  |  |  |
| **procedure_N** | 87611852.5 | 0.0532730171901935 | 0.03925139099645928 | 0.04969676549865229 |  |  |  |
| **ICD_O_aggregate** | 88531234.0 | 0.054347285224936745 | 0.38034058337548476 | 0.34754043126684636 |  |  |  |
| **Positive_anus_test** | 87701322.0 | 0.05786967646250166 | 0.019355926487944698 | 0.02324797843665768 |  |  |  |
| **explicitsepsis** | 87924205.0 | 0.07127588213792732 | 0.0015174506828528073 | 0.0026954177897574125 |  |  |  |
| **ICD_B** | 87650774.0 | 0.0745022238878583 | 0.028730399595346484 | 0.032681940700808626 |  |  |  |
| **ICD_L_aggregate** | 87064425.5 | 0.07588444291364484 | 1.6059011970999832 | 1.4142520215633423 |  |  |  |
| **procedure_L_aggregate** | 88697555.0 | 0.07819133792763425 | 0.3219693137750801 | 0.301044474393531 |  |  |  |
| **procedure_B** | 88060560.0 | 0.08307750814225415 | 0.0005058168942842691 | 0.0 |  |  |  |
| **procedure_E** | 88146770.0 | 0.08808939207675169 | 0.004687236553700893 | 0.003200808625336927 |  |  |  |
| **ICD_P** | 87817501.0 | 0.09436418791229256 | 0.013960546282245826 | 0.017014824797843667 |  |  |  |
| **procedure_E_aggregate** | 88474772.0 | 0.11289011634082372 | 0.10237733940313606 | 0.09046495956873316 |  |  |  |
| **procedure_Y** | 88051656.0 | 0.12112615677947058 | 0.0008430281571404484 | 0.0 |  |  |  |
| **ICD_V_aggregate** | 88190204.0 | 0.12354072362021452 | 0.01834429269937616 | 0.02644878706199461 |  |  |  |
| **ppid** | 89122658.0 | 0.12564669459657068 | 18206.21025122239 | 17977.680592991914 |  |  |  |
| **ICD_X** | 87965671.5 | 0.12875589714260807 | 0.0006069802731411229 | 0.0011792452830188679 |  |  |  |
| **BILIRUBIN KONJUGERT** | 88093271.0 | 0.13892995109233436 | 0.048187489462148035 | 0.02341644204851752 |  |  |  |
| **LAKTAT BLODGASS VENØST** | 87947882.0 | 0.16561759261579934 | 0.0028595515090204023 | 0.004750673854447439 |  |  |  |
| **BILIRUBIN UKONJUGERT** | 88072503.0 | 0.1940392830587837 | 0.08582026639689766 | 0.017857142857142856 |  |  |  |
| **procedure_F** | 87711387.5 | 0.19529044717492594 | 0.04265722475130669 | 0.054750673854447436 |  |  |  |
| **procedure_C_aggregate** | 87405751.0 | 0.19886734002739692 | 1.0435002529084472 | 0.9978099730458221 |  |  |  |
| **procedure_K** | 88257767.5 | 0.20563105976211438 | 0.03146181082448154 | 0.028638814016172506 |  |  |  |
| **PO2** | 88004180.0 | 0.20618354778850345 | 0.0003203506997133704 | 0.0017857142857142857 |  |  |  |
| **PO2_average** | 88004180.0 | 0.20618354778850345 | 0.0003203506997133704 | 0.0017857142857142857 |  |  |  |
| **procedure_J_aggregate** | 87288552.5 | 0.23607070692597942 | 1.3163378856853818 | 1.4081873315363882 |  |  |  |
| **CRP-HØYSENSITIV_average** | 87617765.5 | 0.2654821721488153 | 1.4640893065367517 | 2.224602731023469 |  |  |  |
| **ICD_Y** | 87933223.0 | 0.26898329724714587 | 0.0034732760074186477 | 0.004548517520215634 |  |  |  |
| **ICD_G** | 88317845.0 | 0.2827071964360207 | 0.05648288652841005 | 0.05272911051212938 |  |  |  |
| **Positive_eye_test** | 88114345.5 | 0.2872874511680036 | 0.006305850615410555 | 0.00488544474393531 |  |  |  |
| **Positive_tunge_test** | 87942213.0 | 0.3510812241702028 | 0.004552352048558422 | 0.005390835579514825 |  |  |  |
| **ICD_X_aggregate** | 87891930.5 | 0.3618355912235993 | 0.027887371438206035 | 0.04396900269541779 |  |  |  |
| **centralnervoussystem** | 88042777.0 | 0.3790037807614036 | 0.0006407013994267408 | 0.00033692722371967657 |  |  |  |
| **ICD_T_aggregate** | 88505137.0 | 0.37976528571412604 | 0.9032878098128477 | 0.8237870619946092 |  |  |  |
| **Positive_melk_test** | 87892512.0 | 0.4007442109496363 | 0.017096611026808296 | 0.019036388140161724 |  |  |  |
| **skinandsofttissueinfection** | 88207317.0 | 0.412112542310318 | 0.036519979767324226 | 0.034703504043126686 |  |  |  |
| **procedure_H** | 88039803.5 | 0.4230208254178274 | 0.0007418647782835946 | 0.0005053908355795149 |  |  |  |
| **ICD_V** | 88033860.5 | 0.4380057824949589 | 0.0003709323891417973 | 0.00016846361185983828 |  |  |  |
| **ICD_K** | 88302554.5 | 0.44190018413652754 | 0.1080762097454055 | 0.10545822102425877 |  |  |  |
| **Positive_biopsi_test** | 88051764.5 | 0.4633351502519639 | 0.0018209408194233688 | 0.0011792452830188679 |  |  |  |
| **BILIRUBIN KONJUGERT_average** | 88124808.5 | 0.5144208940802919 | 0.6930146201238765 | 0.7162511230907457 |  |  |  |
| **urinarytractinfection** | 88195591.0 | 0.5457448997207798 | 0.06069802731411229 | 0.05862533692722372 |  |  |  |
| **procedure_D_aggregate** | 88301488.0 | 0.5632163501855436 | 1.0305850615410554 | 1.077156334231806 |  |  |  |
| **ICD_R** | 87807835.0 | 0.5946593273371611 | 0.11849603776766145 | 0.12432614555256065 |  |  |  |
| **procedure_W** | 87853178.0 | 0.6107703872755473 | 0.07941325240263025 | 0.08237870619946092 |  |  |  |
| **TROMBOCYTTER_average** | 87659558.5 | 0.6211694732647592 | 218.80814494768194 | 222.34584165442283 |  |  |  |
| **BILIRUBIN UKONJUGERT_average** | 88084285.5 | 0.647637290804868 | 0.6280325304250444 | 0.34199517070979335 |  |  |  |
| **Positive_faeces_test** | 88101985.5 | 0.6676761620977966 | 0.03550834597875569 | 0.0330188679245283 |  |  |  |
| **intraabdominalinfection** | 88030930.0 | 0.6767134862252595 | 0.0008430281571404484 | 0.0006738544474393531 |  |  |  |
| **lung** | 87969503.5 | 0.6791578297041623 | 0.008632608329118193 | 0.00876010781671159 |  |  |  |
| **ICD_Z** | 88168371.5 | 0.7609910384420818 | 0.21011633788568537 | 0.2120956873315364 |  |  |  |
| **Positive_hud_test** | 88055232.5 | 0.8180324214567738 | 0.023166413758219526 | 0.019878706199460916 |  |  |  |
| **Positive_bein_test** | 88013084.5 | 0.8420913587293486 | 0.00013488450514247176 | 0.00016846361185983828 |  |  |  |
| **Positive_blod_test** | 88019020.5 | 0.8651673486032568 | 0.00020232675771370763 | 0.00016846361185983828 |  |  |  |
| **Positive_led_test** | 88025145.5 | 0.8739460319538371 | 0.0036081605125611194 | 0.002526954177897574 |  |  |  |
| **LAKTAT PNA** | 88013096.0 | 0.893559098563987 | 0.00032709492497049397 | 0.0003537735849056604 |  |  |  |
| **PO2 PNA** | 88013099.0 | 0.8936669377504577 | 0.0027044343281065588 | 0.0026785714285714286 |  |  |  |
| **PH PNA** | 88013102.0 | 0.8937747788343426 | 0.0022559433485078405 | 0.002486522911051213 |  |  |  |
| **procedure_Q_aggregate** | 87957270.5 | 0.9093148056456916 | 1.0701062215477997 | 0.9765835579514824 |  |  |  |
| **Positive_annet_test** | 87997693.5 | 0.9255723338991142 | 0.030382734783341765 | 0.03133423180592992 |  |  |  |
| **endocarditis** | 88019095.5 | 0.9439790784907232 | 0.0012139605462822458 | 0.0011792452830188679 |  |  |  |

**Supplementary Table 6. Statistical summary table for PLOS predictors.**

|  | **Stat** | **P-Value** | **Mean (Class 0)** | **Mean (Class 1)** | **Chi2 Stat** | **Proportion (Class 0)** | **Proportion (Class 1)** |
| --- | --- | --- | --- | --- | --- | --- | --- |
| **ICD_N** | 95197989.5 | 0.0 | 0.08213337322811086 | 0.31861305624576464 |  |  |  |
| **urgency_code** |  | 0.0 |  |  | 15060.36740925732 | 0.795190185884729 | 0.8995934041111362 |
| **care_level_code** |  | 0.0 |  |  | 20916.57221538238 | 0.8121704005685005 | 1.0 |
| **CRP** | 79372162.0 | 0.0 | 28.70623418234407 | 72.40364053911603 |  |  |  |
| **age** | 83386041.5 | 0.0 | 56.747952275872386 | 67.99446577817936 |  |  |  |
| **ICD_I** | 84367690.0 | 0.0 | 0.16811908591091 | 0.7656426473910097 |  |  |  |
| **ICD_J** | 78685693.5 | 0.0 | 0.09671990126042562 | 0.5734131466004065 |  |  |  |
| **LEUKOCYTTER** | 84763030.0 | 0.0 | 6.650528107117489 | 9.91832580377982 |  |  |  |
| **ICD_E** | 98548802.0 | 0.0 | 0.056214234955305385 | 0.2744522249830585 |  |  |  |
| **ICD_F** | 106542708.0 | 1.2745780204429456e-298 | 0.03018289262071287 | 0.14253444770725096 |  |  |  |
| **BILIRUBIN TOTAL** | 94728350.0 | 4.574200391106898e-282 | 4.801454912667839 | 8.15608764400271 |  |  |  |
| **ICD_B** | 108816160.5 | 1.0223196160422629e-279 | 0.01540935781875304 | 0.10356900835780439 |  |  |  |
| **ICD_A** | 108370438.5 | 6.312794597103897e-277 | 0.018326663425215994 | 0.10548904449966116 |  |  |  |
| **procedure_G** | 106514862.0 | 2.1319771605649548e-262 | 0.05281071174776527 | 0.18748588208719222 |  |  |  |
| **procedure_R** | 110410605.5 | 2.3871882748127784e-251 | 0.010883793993342558 | 0.08662751298847979 |  |  |  |
| **KREATININ** | 96841681.0 | 1.5650812595997213e-147 | 60.72928613282467 | 81.04289680746933 |  |  |  |
| **ICD_D** | 110840825.0 | 5.36701768876634e-130 | 0.033137599581104835 | 0.10808674045629094 |  |  |  |
| **ICD_Z_aggregate** | 137035771.0 | 7.7865531223005605e-112 | 10.345588510304074 | 7.167494917551389 |  |  |  |
| **procedure_W** | 109376037.5 | 3.2235409976692005e-111 | 0.07020234132475596 | 0.176304495143438 |  |  |  |
| **procedure_Z_aggregate** | 134461366.5 | 4.036035047790224e-100 | 3.988368178928077 | 2.318838942850689 |  |  |  |
| **procedure_A_aggregate** | 132003182.5 | 4.873650626855162e-98 | 1.042824550248719 | 0.5521798057375198 |  |  |  |
| **procedure_P** | 114908585.0 | 7.57862268887255e-96 | 0.007106257246512324 | 0.04653264061441156 |  |  |  |
| **total_los** | 101058314.0 | 9.069419030854631e-95 | 33.26198401715454 | 39.383395452149834 |  |  |  |
| **ICD_C** | 107698966.5 | 3.584919956974932e-90 | 0.15398137412574336 | 0.4174384459001581 |  |  |  |
| **ICD_J_aggregate** | 102611177.5 | 4.893719415120409e-89 | 2.303100572240715 | 3.206460356900836 |  |  |  |
| **procedure_O_aggregate** | 131538439.0 | 2.5163266207155048e-85 | 3.5620675468451957 | 1.937316467133499 |  |  |  |
| **procedure_M** | 115949153.0 | 6.760917548680435e-83 | 0.0025432920671728315 | 0.04382200135531963 |  |  |  |
| **time_to_last** | 133972477.0 | 1.9140843731189273e-77 | 3380.3748737704304 | 3894.319629545968 |  |  |  |
| **ICD_I_aggregate** | 103916774.0 | 2.8905103160342645e-73 | 5.748513296181322 | 6.7627061215269935 |  |  |  |
| **TROMBOCYTTER** | 103857676.0 | 5.629168095094465e-73 | 137.7152516487764 | 167.3941250658836 |  |  |  |
| **ICD_H** | 123538911.0 | 1.7828958375315663e-70 | 0.058084302651755994 | 0.010503727128981252 |  |  |  |
| **ICD_G** | 112654218.0 | 4.3461133769176205e-64 | 0.04720050865841344 | 0.10018070928393946 |  |  |  |
| **procedure_T** | 116259507.0 | 9.3879165765742e-60 | 0.004039346224333321 | 0.022249830585046308 |  |  |  |
| **ICD_R** | 110724681.0 | 7.875897056614064e-59 | 0.1079777087930583 | 0.19426248023492207 |  |  |  |
| **procedure_J_aggregate** | 129921190.0 | 2.7044977772804e-57 | 1.5247783969779707 | 1.0325276711091032 |  |  |  |
| **TROMBOCYTTER_average** | 131369127.0 | 2.3313029955708225e-54 | 240.50388432112962 | 216.66891694531674 |  |  |  |
| **procedure_D** | 121825118.0 | 2.6018103619645317e-53 | 0.04690129782698134 | 0.0036141856787892477 |  |  |  |
| **ICD_M_aggregate** | 129578997.5 | 8.92186923475864e-51 | 3.6551595167745075 | 2.1044725547775016 |  |  |  |
| **PH_average** | 124151535.5 | 6.56448559736074e-47 | 0.7062891269358192 | 0.34687655356278463 |  |  |  |
| **KREATININ_average** | 106348512.0 | 1.1675794572703303e-46 | 73.41531478357591 | 80.08671630165021 |  |  |  |
| **ICD_P** | 116443247.5 | 2.7155530047661525e-44 | 0.006694842353293189 | 0.0536480686695279 |  |  |  |
| **procedure_O** | 121430659.5 | 7.982073452118447e-43 | 0.041889516400493695 | 0.00677659814772984 |  |  |  |
| **procedure_C** | 120816959.5 | 2.7163297708891187e-41 | 0.02883644387926843 | 0.00056471651231082 |  |  |  |
| **ICD_Y** | 117200816.5 | 3.1323880983157707e-41 | 0.0011968433257283913 | 0.0112943302462164 |  |  |  |
| **ICD_U** | 117379500.5 | 3.440966299590122e-41 | 0.000486217601077159 | 0.008922520894510955 |  |  |  |
| **procedure_D_aggregate** | 126090474.0 | 3.0509426289488542e-40 | 1.1728690578598946 | 0.7773887508470748 |  |  |  |
| **ICD_O** | 116481448.0 | 3.810676065797105e-40 | 0.006021617982570969 | 0.04653264061441156 |  |  |  |
| **procedure_R_aggregate** | 110939991.0 | 3.26003846937548e-39 | 1.4999812993230355 | 0.8129658911226564 |  |  |  |
| **procedure_W_aggregate** | 128579306.5 | 1.200883881827528e-37 | 4.898343120020945 | 4.041676078608538 |  |  |  |
| **ICD_T_aggregate** | 126757144.0 | 1.7854166439199112e-37 | 1.0145865280323147 | 0.6268353286650101 |  |  |  |
| **LAKTAT BLODGASS_average** | 121703031.5 | 6.065096181084578e-36 | 0.07741839739704597 | 0.033637168599254495 |  |  |  |
| **procedure_N** | 115593573.0 | 1.2447722330614733e-34 | 0.024759696301006096 | 0.07273548678563362 |  |  |  |
| **ICD_E_aggregate** | 109338121.0 | 4.539791051429353e-34 | 2.45891461270898 | 2.25626835328665 |  |  |  |
| **ICD_Q_aggregate** | 122378244.0 | 8.602968063673555e-34 | 0.4701350188876837 | 0.11192681274000452 |  |  |  |
| **ICD_K_aggregate** | 127666775.0 | 7.456217274288996e-33 | 2.972285596738602 | 2.0134402529929973 |  |  |  |
| **dementia** | 115751727.5 | 7.546192323698887e-32 | 0.01914949321165426 | 0.04212785181838717 |  |  |  |
| **procedure_Y** | 117356660.5 | 1.3695756937912265e-31 | 0.0022814825896697462 | 0.013214366388073188 |  |  |  |
| **procedure_N_aggregate** | 126390922.0 | 1.0920549880088293e-30 | 1.24460485469574 | 0.8649198102552519 |  |  |  |
| **ICD_L_aggregate** | 125651176.5 | 1.177245308964648e-30 | 1.80689680966451 | 1.0319629545967923 |  |  |  |
| **CRP-HØYSENSITIV** | 120078830.0 | 3.192319758235104e-26 | 0.1292605752328233 | 0.129916421956178 |  |  |  |
| **ICD_T** | 115952214.5 | 8.57550140118662e-25 | 0.022253805587762278 | 0.04574203749717642 |  |  |  |
| **procedure_Q_aggregate** | 124602148.0 | 9.542376654562553e-25 | 1.1845756816396753 | 0.7524282810029366 |  |  |  |
| **ICD_F_aggregate** | 112207274.0 | 2.4853319601980505e-24 | 3.6777125331937017 | 2.434831714479331 |  |  |  |
| **procedure_E_aggregate** | 121797146.0 | 2.4050811800215e-23 | 0.11972173392676815 | 0.06595888863790378 |  |  |  |
| **procedure_L_aggregate** | 122793961.0 | 1.694545352171947e-22 | 0.3632419493585668 | 0.21764174384459 |  |  |  |
| **cardiovascular** | 112345447.0 | 2.155818110915417e-21 | 0.26050043011557017 | 0.31680596340637 |  |  |  |
| **procedure_I_aggregate** | 122737783.0 | 9.77311705373912e-21 | 1.7357594344915286 | 0.9762819064829455 |  |  |  |
| **ICD_W** | 117708841.5 | 2.0831699120914233e-20 | 0.0010098365560833303 | 0.006663654845267676 |  |  |  |
| **procedure_Y_aggregate** | 117485122.5 | 3.4650890173771513e-20 | 0.003964543516475297 | 0.013214366388073188 |  |  |  |
| **ICD_K** | 114390313.0 | 1.3876663631424787e-19 | 0.0996746082208176 | 0.14840749943528347 |  |  |  |
| **ICD_R_aggregate** | 125740588.5 | 1.6678699294112032e-19 | 2.927254366608071 | 2.4175513892026204 |  |  |  |
| **ICD_S** | 115868271.5 | 5.593435362540414e-19 | 0.03631671466507087 | 0.07296137339055794 |  |  |  |
| **total_ICU_LOS** | 125467493.5 | 2.4080320622161896e-18 | 2.05670512772562 | 1.993477524282812 |  |  |  |
| **ICD_N_aggregate** | 112014922.0 | 3.449504130781622e-16 | 4.9294984478438115 | 3.4116783374745876 |  |  |  |
| **ICD_B_aggregate** | 113779449.0 | 3.047344106840584e-14 | 0.5234319482365262 | 0.49932234018522703 |  |  |  |
| **procedure_K_aggregate** | 122825414.0 | 5.2103648081532813e-14 | 2.465833863185847 | 1.606166704314434 |  |  |  |
| **ICD_P_aggregate** | 120468592.0 | 4.2477551708534963e-13 | 0.3342184987096533 | 0.0928393946238988 |  |  |  |
| **procedure_H_aggregate** | 119889807.5 | 8.673615302860865e-13 | 0.046003665332685045 | 0.019087418116105714 |  |  |  |
| **ICD_C_aggregate** | 113625989.0 | 1.0661444554588713e-12 | 8.72132251187493 | 10.225547775016942 |  |  |  |
| **Positive_urin_test** | 113018471.0 | 1.9917977627281193e-12 | 1.492875042076523 | 1.7308561102326632 |  |  |  |
| **procedure_B_aggregate** | 119677854.0 | 1.0605687257404573e-11 | 0.036690728204361 | 0.013327309690535352 |  |  |  |
| **Positive_blood_culture_test_test** | 115543049.5 | 3.8167042278419564e-10 | 0.1278378277293638 | 0.15958888637903773 |  |  |  |
| **LAKTAT BLODGASS VENØST_average** | 119246134.5 | 5.386184133886086e-10 | 0.02010272014704075 | 0.009950304946916651 |  |  |  |
| **ICD_H_aggregate** | 122295141.0 | 3.1611893061484266e-09 | 2.13744997568912 | 1.6078608538513666 |  |  |  |
| **LAKTAT_average** | 119223664.0 | 1.152058245330432e-08 | 0.01810639331407534 | 0.008196797429912406 |  |  |  |
| **BILIRUBIN TOTAL_average** | 123115302.0 | 1.1654498207264866e-08 | 14.071635401016888 | 10.99022929888353 |  |  |  |
| **infection** | 122341118.0 | 1.727267496832374e-08 | 0.42529079552679805 | 0.3945109555003388 |  |  |  |
| **urinarytractinfection** | 120328517.0 | 7.358697488484514e-08 | 0.07240902120656768 | 0.05556810481138468 |  |  |  |
| **procedure_F** | 116860696.5 | 7.750297588524213e-08 | 0.03968283651868198 | 0.07363903320533093 |  |  |  |
| **LAKTAT PNA** | 118078812.0 | 7.988092900518435e-08 | 0.0009013726296891948 | 0.004077253218884121 |  |  |  |
| **PH PNA** | 118078884.0 | 8.048159682572808e-08 | 0.005544376706436772 | 0.023493336345154728 |  |  |  |
| **PO2 PNA** | 118078941.5 | 8.096443857468697e-08 | 0.006893069529116953 | 0.028811836458098032 |  |  |  |
| **procedure_I** | 118890379.0 | 8.12172023187473e-08 | 0.006283427460074055 | 0.0028235825615541 |  |  |  |
| **Positive_edta_test** | 116227132.0 | 9.80982098562563e-08 | 0.1379361932901971 | 0.190083578043822 |  |  |  |
| **Positive_annet_test** | 119581045.5 | 1.1708710888154185e-07 | 0.03418483749111718 | 0.022362773887508472 |  |  |  |
| **ICD_M** | 120354319.5 | 1.6275054305295746e-07 | 0.08415304634027752 | 0.06584594533544161 |  |  |  |
| **ICD_W_aggregate** | 116908072.0 | 3.745526202304411e-07 | 0.04304895837229308 | 0.05624576462615767 |  |  |  |
| **Positive_faeces_test** | 119547093.5 | 5.421373292817618e-07 | 0.03979504058046901 | 0.025186356449062572 |  |  |  |
| **procedure_C_aggregate** | 121124123.0 | 6.622121129208306e-07 | 1.1337846430040768 | 0.827874407047662 |  |  |  |
| **skinandsofttissueinfection** | 119761509.0 | 7.986223224331905e-07 | 0.04271234618693197 | 0.030946464874632935 |  |  |  |
| **Positive_ear_test** | 118994121.0 | 2.0189271172738994e-06 | 0.014100310431237611 | 0.006437768240343348 |  |  |  |
| **procedure_U** | 117798451.5 | 2.4876187008266273e-06 | 0.005909413920783932 | 0.011520216851140727 |  |  |  |
| **ICD_A_aggregate** | 115503784.5 | 4.34823895782659e-06 | 0.42487938063357894 | 0.43641292071380167 |  |  |  |
| **ICD_U_aggregate** | 117616050.5 | 4.725322764524521e-06 | 0.03250177656431163 | 0.03704540320758979 |  |  |  |
| **CRP-HØYSENSITIV_average** | 120260252.5 | 6.363461058604621e-06 | 1.5821364758516838 | 1.763917009737119 |  |  |  |
| **ICD_O_aggregate** | 119742479.0 | 1.1661248728231539e-05 | 0.41695029360062835 | 0.31251411791280775 |  |  |  |
| **procedure_M_aggregate** | 119725601.0 | 1.225471584302843e-05 | 0.3984366234057673 | 0.29591145245086964 |  |  |  |
| **cancer** | 116692968.5 | 4.043055995151331e-05 | 0.08688334517709541 | 0.10243957533318274 |  |  |  |
| **ICD_L** | 119440186.0 | 8.456746114730424e-05 | 0.04256274077121592 | 0.03297944431895189 |  |  |  |
| **BILIRUBIN KONJUGERT_average** | 119111476.5 | 0.00014324691828042122 | 0.7934371250796055 | 0.5304794877586836 |  |  |  |
| **ICU_LOS** | 121311716.0 | 0.0002560669292225152 | 0.8228360199972596 | 1.0409372411716018 |  |  |  |
| **procedure_E** | 118725365.0 | 0.0003130360907041235 | 0.006582638291506153 | 0.0040659588886379034 |  |  |  |
| **ICD_X_aggregate** | 118935719.0 | 0.00038147284057993513 | 0.037139544451509146 | 0.013101423085611023 |  |  |  |
| **LEUKOCYTTER_average** | 115413829.5 | 0.00042838870124627717 | 8.811861295371932 | 9.012595321021971 |  |  |  |
| **ICD_Z** | 116307536.0 | 0.000436946418272741 | 0.20589445337921233 | 0.23864919810255253 |  |  |  |
| **ICD_V** | 118235178.5 | 0.00044533591380206683 | 0.0003740135392901223 | 0.0014682629320081318 |  |  |  |
| **Positive_plasma_test** | 119862843.0 | 0.0007745967182708298 | 0.17997531510640685 | 0.15958888637903773 |  |  |  |
| **BILIRUBIN UKONJUGERT_average** | 118948431.5 | 0.0009284026123674081 | 0.6738136174497771 | 0.3126910624199985 |  |  |  |
| **procedure_X** | 118293342.0 | 0.0010391463433691876 | 7.480270785802445e-05 | 0.0006776598147729839 |  |  |  |
| **ICD_Q** | 118874764.5 | 0.0015625111298600254 | 0.014212514493024648 | 0.010955500338829907 |  |  |  |
| **procedure_A** | 118989492.5 | 0.002421293159447823 | 0.033100198227175824 | 0.02371809351705444 |  |  |  |
| **procedure_L** | 118698160.0 | 0.002576012972590941 | 0.00957474660582713 | 0.0054212785181838715 |  |  |  |
| **Positive_melk_test** | 118878761.5 | 0.0027728610669722 | 0.019186894565583274 | 0.01389202620284617 |  |  |  |
| **ICD_G_aggregate** | 120216650.5 | 0.003604564275183135 | 1.7143658600441336 | 1.245199909645358 |  |  |  |
| **Positive_eye_test** | 118680417.5 | 0.003956944491768414 | 0.0070314545386542995 | 0.004178902191100068 |  |  |  |
| **procedure_Z** | 117667248.5 | 0.006396134575241677 | 0.03235217114859558 | 0.040659588886379036 |  |  |  |
| **LAKTAT** | 118519199.0 | 0.006727068678707766 | 0.002633055316602461 | 0.0010164897221594759 |  |  |  |
| **CRP_average** | 116183368.0 | 0.009211567797416978 | 54.46089844400552 | 61.076474013775716 |  |  |  |
| **procedure_G_aggregate** | 116577558.5 | 0.010126399797655014 | 1.422111680442832 | 1.4405918229049017 |  |  |  |
| **Gender** |  | 0.010630521431399689 |  |  | 6.526059351565154 | 0.5285933350787299 | 0.5128755364806867 |
| **procedure_B** | 118448812.0 | 0.012107976666971911 | 0.0007480270785802446 | 0.0 |  |  |  |
| **ICD_V_aggregate** | 118704324.5 | 0.012336056690957527 | 0.020907356846317837 | 0.01863564490625706 |  |  |  |
| **Positive_naso_test** | 119341358.0 | 0.0222308511537434 | 0.131465759060478 | 0.11091032301784504 |  |  |  |
| **centralnervoussystem** | 118444297.5 | 0.027342277562341536 | 0.0007854284325092568 | 0.00011294330246216399 |  |  |  |
| **ICD_S_aggregate** | 119886578.0 | 0.02858702554316921 | 1.0404682649511912 | 0.8483171447933138 |  |  |  |
| **ICD_D_aggregate** | 116833410.0 | 0.034885409721731576 | 1.5434416725885478 | 1.4260221368872825 |  |  |  |
| **LAKTAT BLODGASS VENØST** | 118224968.5 | 0.03825551761110994 | 0.00278079066462206 | 0.008086740456290941 |  |  |  |
| **Positive_led_test** | 118496392.5 | 0.05841374183044616 | 0.0042637543479073945 | 0.0020329794443189517 |  |  |  |
| **procedure_J** | 117762025.0 | 0.06991308124321848 | 0.06466694094326214 | 0.0887734357352609 |  |  |  |
| **organdysfunction** | 117591391.0 | 0.07595536612403558 | 0.10401316527658301 | 0.11203975604246669 |  |  |  |
| **PO2** | 118351330.5 | 0.08226683864783566 | 0.0 | 0.0011971990060989383 |  |  |  |
| **Positive_hud_test** | 118694484.5 | 0.10008115737189566 | 0.02498410442458017 | 0.018861531511181386 |  |  |  |
| **procedure_Q** | 118667309.0 | 0.12139735482286573 | 0.02180498934061413 | 0.024847526541676077 |  |  |  |
| **Positive_biopsi_test** | 118452452.0 | 0.12714060868128027 | 0.0019822717582376483 | 0.001242376327083804 |  |  |  |
| **BILIRUBIN KONJUGERT** | 118460947.0 | 0.13444379474569762 | 0.060627594718928826 | 0.0734131466004066 |  |  |  |
| **procedure_T_aggregate** | 119355658.0 | 0.1354958655131674 | 0.7169465534652354 | 0.6984413824260222 |  |  |  |
| **PH** | 118730968.0 | 0.14696812999364892 | 0.2360427497475404 | 0.21358952639108517 |  |  |  |
| **Positive_anus_test** | 118641341.5 | 0.15676602305759826 | 0.02128137038560796 | 0.01840975830133273 |  |  |  |
| **Positive_bein_test** | 118386834.0 | 0.1981619747409854 | 0.00018700676964506115 | 0.0 |  |  |  |
| **LAKTAT PNA_average** | 118196777.5 | 0.22031269114582175 | 0.01162995100422635 | 0.012900948723740686 |  |  |  |
| **PH PNA_average** | 118201781.5 | 0.2350956535605564 | 0.06444515091446323 | 0.0746126044725548 |  |  |  |
| **PO2 PNA_average** | 118202753.5 | 0.23790053673872802 | 0.08373602124396907 | 0.09208267449740233 |  |  |  |
| **procedure_H** | 118395508.5 | 0.30102401130919487 | 0.0005610203089351835 | 0.00033882990738649197 |  |  |  |
| **Positive_hal_test** | 118049786.0 | 0.3250781646483383 | 0.06264726783109549 | 0.0690083578043822 |  |  |  |
| **explicitsepsis** | 118305384.5 | 0.34577524043785324 | 0.001795264988592587 | 0.0023718093517054437 |  |  |  |
| **PO2_average** | 118355757.0 | 0.41114845963622504 | 0.0003553128623256162 | 0.0011971990060989383 |  |  |  |
| **Positive_blod_test** | 118382319.5 | 0.4180232440332292 | 0.00026180947750308563 | 0.00011294330246216399 |  |  |  |
| **endocarditis** | 118399327.5 | 0.49714012846814815 | 0.001309047387515428 | 0.0010164897221594759 |  |  |  |
| **intraabdominalinfection** | 118395075.5 | 0.5042989701858427 | 0.0010472379100123425 | 0.000790603117235148 |  |  |  |
| **BILIRUBIN UKONJUGERT** | 118399246.0 | 0.5331624599533715 | 0.09058607921606762 | 0.03648068669527897 |  |  |  |
| **prior_comorbidities_counts** | 117890973.5 | 0.548139065282932 | 0.9644313124135093 | 0.9887056697537836 |  |  |  |
| **lung** | 118443351.5 | 0.5500220685373689 | 0.00920073306653701 | 0.00790603117235148 |  |  |  |
| **ICD_Y_aggregate** | 118178540.5 | 0.5887821160279978 | 0.07513932004338557 | 0.07296137339055794 |  |  |  |
| **procedure_U_aggregate** | 117996337.5 | 0.5909049612977192 | 0.8632606500355313 | 0.8851366613959792 |  |  |  |
| **sepsis** | 118315266.5 | 0.6680829726032309 | 0.006246026106145042 | 0.006663654845267676 |  |  |  |
| **ICD_X** | 118350893.0 | 0.69526997147073 | 0.0005610203089351835 | 0.0006776598147729839 |  |  |  |
| **procedure_P_aggregate** | 118579437.0 | 0.7075120564045225 | 0.39495829749036915 | 0.3930426925683307 |  |  |  |
| **LAKTAT BLODGASS** | 118411030.0 | 0.7356228835802239 | 0.015598234656094553 | 0.023911979519614483 |  |  |  |
| **ppid** | 118105057.0 | 0.7566690917349355 | 18158.179750906984 | 18198.03761011972 |  |  |  |
| **pneumonia** | 118428296.0 | 0.7620970185174154 | 0.021617982570969068 | 0.021007454257962504 |  |  |  |
| **Positive_tunge_test** | 118338929.5 | 0.7874408175199241 | 0.0050117814264876385 | 0.0054212785181838715 |  |  |  |
| **Positive_bronki_test** | 118416143.5 | 0.7907694307663935 | 0.020757751430601788 | 0.021007454257962504 |  |  |  |
| **procedure_F_aggregate** | 118426500.5 | 0.9314486029541019 | 1.6005535400381494 | 1.3639033205330924 |  |  |  |
| **procedure_X_aggregate** | 118380923.5 | 0.948342968715609 | 0.03635411601899989 | 0.03602891348543032 |  |  |  |
| **procedure_K** | 118377710.5 | 0.9520387674417476 | 0.02584433556494745 | 0.030720578269708608 |  |  |  |

**Supplementary Table 7. Disease Groups**

| **S/No.** | **Disease** | **ICD-10 codes** |
| --- | --- | --- |
| **1** | **Explicit Sepsis** | 'A021','A207','A217','A227','A241','A267','A282','A327','A394','A40','A41','A427','B007','B377' |
| **2** | **Organ dysfunction** | 'D695','E872','G934','I46','I959','J80','J952','J96','K720','K729','N00', 'N17','R090','R092','R400','R401','R402','R41', 'R55', 'R57', 'R651', 'R572' |
| **3** | **Implicit Sepsis** | Organ dysfunction + Infection |
| **4** | **Infection** | 'A00','A01','A02','A03','A04','A05','A06','A07','A08','A09','A19', 'A20','A21','A22','A23','A24','A25','A26','A27','A28','A30','A31','A32','A36', 'A37','A38','A39','A42', 'A43', 'A44', 'A46','A48','A49','A54','A59','A690','A691','A699','A70','A74', 'A75', 'A77','A78','A79','A80','A81','A83','A84','A85','A86','A87', 'A88','A89','A90','A91','A92','A93','A94','A95','A96','A97','A98', 'A99','B00','B01','B02','B03','B04','B05','B06','B08','B09','B10','B25', 'B26','B27','B33','B34','B37','B38','B39','B40','B41','B42','B43','B44', 'B45','B46','B48','B49','B50','B54','B55','B57','B58','B59','B60','B64', 'B67','B95','B96','B97','B99','G00','G01','G02','G03','G04','G05','G06', 'G07','G08','H050', 'H602','H700','I00','I33','I38','I39','I400','J01','J02','J03',  'J04','J05','J06','J09','J10','J11','J12','J13','J14','J15','J16','J17','J18','J19','J20',  'J21','J22','J36','J390','J391','J85','J86','K35','K36','K37','K61','K630',   'K631','K65','K750', 'K810', 'K830','L02','L03','L030','L04','L08','M00','M01','M86','N10','N151', 'N30','N390','N410','N412','N413','N45','N70','N71','N72','N73','N74', 'N980','O030','O035','O045','O080','O23','O753', 'O85',  'O86','O883','O91','O98','R02','T802','T814','T826''T827','T835', 'T836','T845','T846','T847','T857','T880', 'U04', 'M726', 'N49', 'U071', 'U072' |
| **5** | **Cancer** | 'C00', 'C01', 'C02', 'C03', 'C04', 'C05', 'C06', 'C07', 'C08', 'C09', 'C10', 'C11', 'C12', 'C13', 'C14', 'C15', 'C16', 'C17', 'C18', 'C19','C20', 'C21', 'C22', 'C23', 'C24', 'C25', 'C26', 'C27', 'C28', 'C29',           'C30', 'C31', 'C32', 'C33', 'C34', 'C35', 'C36', 'C37', 'C38', 'C39', 'C40', 'C41', 'C42', 'C43', 'C44', 'C45', 'C46', 'C47', 'C48', 'C49','C50', 'C51', 'C52', 'C53', 'C54', 'C55', 'C56', 'C57', 'C58', 'C59',            'C60', 'C61', 'C62', 'C63', 'C64', 'C65', 'C66', 'C67', 'C68', 'C69', 'C70', 'C71', 'C72', 'C73', 'C74', 'C75', 'C76', 'C77', 'C78', 'C79','C80', 'C81', 'C82', 'C83', 'C84', 'C85', 'C86', 'C87', 'C88', 'C89',           'C90', 'C91', 'C92', 'C93', 'C94', 'C95', 'C96', 'C97', 'D32', 'D33', 'D35', 'D42', 'D43', 'D44', 'D45', 'D46', 'D47' |
| **6** | **Diabetes** | 'E10', 'E11', 'E12', 'E13', 'E14' |
| **7** | **Cardiovascular** | 'G45', 'H34', 'I00', 'I01', 'I02', 'I03', 'I04', 'I05', 'I06', 'I07', 'I08', 'I09', 'I10', 'I11', 'I12', 'I13', 'I14', 'I15', 'I16', 'I17', 'I18', 'I19', 'I20', 'I21', 'I22', 'I23', 'I24', 'I25', 'I26', 'I27', 'I28', 'I29', 'I30',                    'I31', 'I32', 'I33', 'I34', 'I35', 'I36', 'I37', 'I38', 'I39', 'I40', 'I41', 'I42', 'I43', 'I44', 'I45', 'I46', 'I47', 'I48', 'I49', 'I50', 'I51', 'I52', 'I53', 'I54', 'I55', 'I56', 'I57', 'I58', 'I59', 'I60',                    'I61', 'I62', 'I63', 'I64', 'I65', 'I66', 'I67', 'I68', 'I69', 'I70', 'I71', 'I72', 'I73', 'I74', 'I75', 'I76', 'I77', 'I78', 'I79', 'I80', 'I81', '182', 'I83', 'I84', 'I85', 'I86', 'I87', 'I88', 'I89', 'I90', 'I91', 'I92', 'I93',                     'I94', 'I95', 'I96', 'I97', 'I98', 'I99' |
| **8** | **Lung** | 'J41', 'J42', 'J43', 'J44', 'J45', 'J46', 'J47', 'J84', 'J98' |
| **9** | **Dementia** | 'F00', 'F02', 'F03', 'G30', 'G31' |
| **10** | **Kidney** | 'N18' |
| **11** | **Liver** | 'K70', 'K72' |
| **12** | **Immune system** | 'D80', 'D81', 'D82', 'D83', 'D84', 'Z94' |

**Supplementary List 1. Groups of various microbiology tests**

**'annet'**: ['ABSCESS', 'ABSCESS (TBA)', 'ABSCESS (VAB)', 'ACITES PÅ BL.K.FLASKE', 'AMPUTASJONSSTUMP', 'ANNET', 'ANNET (ANS)', 'ANNET (VAN)'],

**'anus'**: ['ANUSSEKRET', 'ANUSSEKRET (ANUM)', 'ANUSSEKRET (ANUP)', 'ANUSSEKRET (ANUS)', 'ANUSSEKRET (VANU)', 'ASCITES', 'ASPIRAT', 'ASPIRAT (VAS)', 'ASPIRAT PÅ BL.K.FLASKE', 'AUTOPSIMATRIALE', 'AUTOPSIMATRIALE (VAU)', 'AXILLE', 'AXILLE (MRSA)', 'BAKTERIESTAMME'],

**'bein'**: ['BEIN FRA BEINBANK', 'BEIN TIL BEINBANK', 'BEINMARG', 'BEINMARG (BEM)', 'BEINVEV'],

**'biopsi'**: ['BIHULESEKRET (BIH)', 'BIOPSI', 'BIOPSIMATERIALE', 'BIOPSIMATERIALE (TBI)', 'BIOPSIMATERIALE (VBI)'],

**'blod'**: ['BLOD - ISOLATOR', 'BLOD - ISOLATOR (TBLI)', 'BLODKULTUR (BLS)', 'BLODKULTUR (BLS1)'],

**'blood_culture_test': ['BLODKULTUR'],**

**'bronki'**: ['BRONKIALBØRSTE (VBR)', 'BRONKIALSKYLLEVÆSKE', 'BRONKIALSKYLLEVÆSKE (TBS)', 'BRONKIALSKYLLEVÆSKE (VBS)'],

**'melk'**: ['BRYSTMELK', 'BURSAVÆSKE', 'CERVIX (CERC)', 'CERVIX (CERM)', 'CERVIX-/URETHRASEKRET (CU', 'CERVIX-/VAGINALSEKRET', 'CERVIXSEKRET', 'CERVIXSEKRET (VCE)', 'CH-UROGENITALSEKRET', 'CH-UROGENITALSEKRET (UROM', 'CORNEAAVSKRAP', 'CYSTEINNHOLD', 'DIALYSAT PÅ BL.K.FLASKE', 'DIALYSEVÆSKE', 'DRENSPISS', 'DRENSVÆSKE', 'DRENSVÆSKE PÅ BL.K.FLASKE'],

**'edta'**: ['EDTA-BLOD', 'EDTA-BLOD (BEDT)', 'EDTA-BLOD (EDTA)', 'EDTA/UTSTRYK MALARIA', 'EJAKULAT (EJA)', 'EKSPEKTORAT', 'EKSPEKTORAT (EXS)', 'EKSPEKTORAT (TEX)', 'EKSPEKTORAT (VEK)', 'ELUAT', 'FISTEL', 'FOSTERVANN', 'FOSTERVANN (VFO)'],

**'faeces'**: ['FÆCES', 'FÆCES (FÆ)', 'FÆCES (FÆCB)', 'FÆCES (FÆCD)', 'FÆCES (FÆCP)', 'FÆCES (FÆD)', 'FÆCES (FÆFP)', 'FÆCES (FÆP)', 'FÆCES (VFÆ)', 'FÆCES (VFÆB)', 'FÆCES (VFÆN)', 'FÆCES (VFÆV)', 'GALLEVEISPRØVE', 'GENITALSEKRET (VGF)'],

**'hal'**: ['HALSSEKRET', 'HALSSEKRET (HALC)', 'HALSSEKRET (HALG)', 'HALSSEKRET (HALM)', 'HALSSEKRET (HAS)', 'HALSSEKRET (MRSA)', 'HALSSEKRET (VHA)', 'HALSSEKRET (VHAR)'],

**'hud'**: ['HUD', 'HUD (MRSA)', 'HUDAVSKRAP', 'HUDAVSKRAP (VHU)', 'HÅR', 'INDUSERT SPUTUM', 'INDUSERT SPUTUM (TSPU)', 'INNSTIKKSTED', 'KATETERSPISS', 'LARYNXSEKRET'],

**'led'**: ['LEDDVÆSKE', 'LEDDVÆSKE - ANRIKET', 'LGV (LYMFOGRAN. VENEREUM)', 'LYSAT', 'LYSKE (LYS)', 'MELK', 'MORSMELK'],

**'naso'**: ['MRSA REFERANSESTAMME (MRS', 'MUNNHULE', 'MUNNSEKRET', 'MUNNSEKRET (VMU)', 'NASOFARYNKS- OG HALSPRØVE', 'NASOPHARYNXASPIRAT (LUFA)', 'NASOPHARYNXASPIRAT (LUFT)', 'NASOPHARYNXASPIRAT (NAI)', 'NASOPHARYNXSEKRET', 'NASOPHARYNXSEKRET (NAS)', 'NASOPHARYNXSEKRET (VNAR)', 'NAVLESEKRET', 'NAVLESTRENG - BIT', 'NEGL', 'NEGL (NEGL)', 'NESESEKRET', 'NESESEKRET (DIA)', 'NESESEKRET (MRSA)'],

**'plasma'**: ['NONHUMANT MATERIALE', 'OPERASJONS-SÅR', 'OPPKAST', 'PACEMAKERTRÅD', 'PARAFININNSTØPT VEV', 'PD-DIALYSAT', 'PERICARDVÆSKE', 'PERICARDVÆSKE (VPEC)', 'PERINEUM', 'PERINEUM (MRSA)', 'PERITONEALVÆSKE', 'PERITONSILLÆRABSESS', 'PLACENTA', 'PLASMA', 'PLASMA (PLAS)', 'PLEURAV. PÅ BL.K.FLASKE', 'PLEURAVÆSKE', 'PLEURAVÆSKE (TPL)', 'PLEURAVÆSKE (VPLE)', 'PUSS (PSD)', 'PUSS/SEKRET', 'PUSS/SEKRET (TPU)', 'PUSS/SEKRET (VPUS)', 'RECTUMSEKRET (PREC)', 'SEKRET (SEK)', 'SERUM', 'SERUM (SSE)', 'SKYLLEVÆSKE (VSK)', 'SOPPKULTUR', 'SPINALVÆSKE', 'SPINALVÆSKE (VSP)', 'SPIRAL', 'SPISS AV CVK', 'SPUTUM (VSPU)', 'SÅRSEKRET', 'SÅRSEKRET (MRSA)', 'SÅRSEKRET (SÅS)', 'SÅRSEKRET (VSÅ)', 'TRACHEALASPIRAT', 'TRANSPLANTATMEDIUM', 'TRANSTRACHEALT ASPIRAT (T', 'TUBESEKRET (VTU)'],

**'tunge'**: ['TUNGESEKRET', 'TUNGESEKRET (TUNS)', 'TUNGESEKRET (VTUN)', 'TÅREVÆSKE'],

**'urin'**: ['URETHRA', 'URETHRA (UREM)', 'URETHRASEKRET', 'URETHRASEKRET (VUR)', 'URIN', 'URIN (CHLAMYDIA)', 'URIN (CHU)', 'URIN (CLUM)', 'URIN (CLUR)', 'URIN (TUR)', 'URIN (URS)', 'URIN (VURI)', 'URIN BLÆREPUNKSJON', 'URIN/TRANSPORTAGAR (URC)', 'UROGENITALSEKRET', 'UROGENITALSEKRET (URO)', 'USPESIFISERT (USP)', 'USPESIFISERT (VUS)', 'USPESIFISERT(MRSA)', 'UTERUSSEKRET (PUTE)', 'UTSTRYK', 'VAGINA', 'VAGINA (VAGM)', 'VAGINALPENSEL/URIN', 'VAGINALSEKRET', 'VAGINALSEKRET (CHP)', 'VAGINALSEKRET (VVG)', 'VESIKKELINNHOLD', 'VESIKKELINNHOLD (VESB)', 'VEV (TVV)', 'VEV/BIOPSI', 'VEV/BIOPSI (BIO)', 'VULVA (VVU)'],

**'ear'**: ['ØRESEKRET', 'ØRESEKRET (ØRS)', 'ØRESEKRET, HØYRE ØRE', 'ØRESEKRET, VENSTRE ØRE'],

**'eye'**: ['ØYEKAMMERVÆSKE', 'ØYESEKRET', 'ØYESEKRET (VØY)', 'ØYESEKRET (ØYC)', 'ØYESEKRET (ØYM)', 'ØYESEKRET - HØYRE ØYE', 'ØYESEKRET - HØYRE ØYE (VØ', 'ØYESEKRET - VENSTRE ØYE', 'ØYESEKRET - VENSTRE ØYE (' ]}

**Supplementary Table 8. Contaminant microbes:** List of microbes identified as contaminants

| **Contaminants** | 'BACILLUS CEREUS', 'STREPTOCOCCUS EQUI SSP EQUI', 'BACILLUS CIRCULANS', 'STREPTOCOCCUS EQUI SSP ZOOEPIDEMICUS', 'BACILLUS FIRMUS',​ 'STREPTOCOCCUS GORDONII', 'BACILLUS LICHENIFORMIS', 'STREPTOCOCCUS INTERMEDIUS', 'BACILLUS MEGATERIUM • STREPTOCOCCUS MITIS', 'BACILLUS PUMILUS', ​ 'STREPTOCOCCUS MITIS', 'STREPTOCOCCUS ORALIS', 'BACILLUS SPECIES', 'STREPTOCOCCUS MUTANS', 'BACILLUS SPHAERICUS', 'STREPTOCOCCUS PYOGENES', ​ 'BACILLUS SUBTILIS', 'STREPTOCOCCUS SALIVARIUS', 'COAGULASE NEGATIVE STAPHYLOCOCCUS', 'STREPTOCOCCUS SANGUINIS', 'CORYNEBACTERIUM JEIKEIUM',​ 'STREPTOCOCCUS VESTIBULARIS', 'CORYNEBACTERIUM SPECIES', 'STREPTOCOCCUS VIRIDANS GROUP', 'CORYNEBACTERIUM XEROSIS', 'MICROCOCCUS LUTEUS', ​ 'MICROCOCCUS LYLAE', 'MICROCOCCUS LUTEUS', 'STAPHYLOCOCCUS ARLETTAE','MICROCOCCUS SPECIES', 'STAPHYLOCOCCUS CAPRAE', 'STAPHYLOCOCCUS SPP', 'STAPHYLOCOCCUS CARNOSUS SSP CARNOSUS', 'STAPHYLOCOCCUS AUREUS', 'STAPHYLOCOCCUS GALLINARUM', 'STAPHYLOCOCCUS AURICULARIS', ​ 'STAPHYLOCOCCUS HOMINIS SSP HOMINIS','STAPHYLOCOCCUS CAPITIS', 'STREPTOCOCCUS ALACTOLYTICUS', 'STAPHYLOCOCCUS COHNII SSP COHNII', 'STREPTOCOCCUS CRISTATUS',​ 'STAPHYLOCOCCUS COHNII SSP UREALYTICUS',​ 'STREPTOCOCCUS CONSTELLATUS SSP CONSTELLATUS','STAPHYLOCOCCUS EPIDERMIDIS', 'STREPTOCOCCUS CONSTELLATUS SSP PHARYNGIS', 'STAPHYLOCOCCUS HAEMOLYTICUS', ​ 'STREPTOCOCCUS HYOINTESTINALIS', 'STAPHYLOCOCCUS HOMINIS', 'STREPTOCOCCUS MITIS/STREPTOCOCCUS ORALIS', 'STAPHYLOCOCCUS INTERMEDIUS', 'STREPTOCOCCUS PARASANGUINIS', 'STAPHYLOCOCCUS KLOOSII', 'STREPTOCOCCUS PLURANIMALIUM', 'STAPHYLOCOCCUS LENTUS', ​ 'STREPTOCOCCUS SOBRINUS', 'STAPHYLOCOCCUS LUGDUNENSIS','STREPTOCOCCUS THERMOPHILUS', 'STAPHYLOCOCCUS SACCHAROLYTICUS', 'STREPTOCOCCUS THORALTENSIS', 'STAPHYLOCOCCUS SAPROPHYTICUS', 'STREPTOCOCCUS SPP', 'STAPHYLOCOCCUS SCHLEIFERI', 'DIPHTHEROIDS SPP', 'STAPHYLOCOCCUS SCIURI', 'CORYNEBACTERIUM STRIATUM', 'STAPHYLOCOCCUS SIMULANS', 'NON HAEMOLYTIC STREPTOCOCCIS', 'STAPHYLOCOCCUS SPECIES', 'BABESIA SPP', 'STAPHYLOCOCCUS WARNERI', ​ 'CORYNEBACTERIUM MINUTISSMUM', 'STAPHYLOCOCCUS XYLOSUS','CORYNEBACTERIUM AMYCOLATUM', 'STREPTOCOCCUS AGALACTIAE', 'MICROMONAS MICROS',​ 'STREPTOCOCCUS ANGINOSUS',​ 'STAPHYLOCOCCUS PASTEURI', 'STREPTOCOCCUS CONSTELLATUS' |
| --- | --- |
