# Supplementary material for: In-hospital mortality, readmission, and prolonged length of stay risk prediction leveraging historical electronic patient records
Source: JAMIA Open. 2024 Sep 14;7(3):ooae074. doi: 10.1093/jamiaopen/ooae074 (PMC11401612; doi:10.1093/jamiaopen/ooae074)
Supplement: ooae074_Supplementary_Data [file ooae074_supplementary_data.zip › Supplementary_Information_1.docx]

Inhospital Mortality, Readmission, and Prolonged Length of Stay Risk Prediction Leveraging Historical Electronic Patient Records

Supplementary Information 1

* corresponding author: rajeev.bopche@ntnu.no

**1. SUPPLEMENTARY METHODS**

## **1.1. Data preprocessing:**

Initially stored in a Postgres database, the medical data was converted into CSV files to facilitate easier manipulation and access. Utilizing Python libraries such as Pandas and NumPy, the CSV files were converted to dataframes for further processing. The episode discharge summaries required several data cleaning steps to ensure the quality and relevance of the data: Relevant patient information such as identifiers, admission and discharge times, and diagnostic codes were retained. Instances of missing identifiers were addressed by replacing empty strings with NaN values and removing these records. Data were organized by patient identifier and admission/discharge times to maintain coherent episode tracking. Non-standard characters within diagnostic codes, such as semicolons and commas, were standardized to spaces, and any duplicates were removed. The timestamps were converted into datetime format, facilitating the calculation of the length of stay in hours for each episode. The duration of each ICU stay was calculated in hours, along with the total count of each type of hospital admission per patient. The request dates in laboratory and microbiology test results were standardized to datetime objects and used to create event logs of tests per patient. Specialized functions were designed to clean the laboratory and microbiology test table entries. This function performed tasks to remove any non-numeric characters, which could represent encoding errors or artifacts from data entry. It standardized decimal point characters by replacing commas with periods, necessary for consistent numerical representation across different regions that may use varying formats for decimal points. The microbiology tests results column was processed to standardize and clean the values, categorizing them as ‘positive’, ‘negative’, or ‘contaminant’ based on the list of contaminants in Supplementary Table 7. Four event logs were created for each patient ID, from episode discharge summaries, ICU admissions, laboratory tests, and microbiology tests, where hospital visits/admissions, ICU admissions, and particular laboratory and microbiology tests were considered separate and overlapping medical events, respectively.

*Discharge Summaries Event Log:* Captures patient discharge information, including admission and discharge times, diagnostic and procedural codes, urgency, and care level codes.

*ICU Admissions Event Log:* Records details of each ICU stay, including the duration in hours, the total count of ICU admissions per patient, and the total length of ICU stays per patient.

*Laboratory Tests Event Log:* Includes results of various laboratory tests standardized and organized chronologically for each patient.

*Microbiology Tests Event Log:* Consists of microbiology test results, grouped by collection sample type, and categorizes them based on outcomes such as ‘positive’, ‘negative’, or ‘contaminant’.

**1.2. Data Enhancement:**

This approach enhanced the dataset with additional attributes derived from diagnostic and procedure codes and laboratory and microbiology test results. From the discharge summaries, counts of the occurrences of different ICD and procedure codes were classified according to the initial character (alphabetic) corresponding to the various chapters of the ICD-10. Each patient record was expanded with new columns corresponding to each initial character, incrementing the count for each instance where a character leading the code was present in the primary and secondary diagnosis codes for the corresponding discharge summary entry. Further stratification was conducted by categorizing the ICD-10 codes in the discharge summaries into disease groups pertinent to clinical significance, such as 'explicit sepsis', 'infection', and 'organ dysfunction', among others. The table depicting the ICD-10 codes selected for different disease groups is given in (Supplementary Table 6). Laboratory test results were organized using pivot tables, ensuring a structured format for analysis. Tests such as 'Bilirubin' (total, conjugated, and unconjugated), 'C-Reactive Protein' (CRP), and 'Lactate' (various measurements) were included, alongside white blood cell count (leukocytes), platelet count (thrombocytes), and blood gas measurements (pH, PO2).

Similarly, microbiology test results were consolidated to reflect various sample types such as blood, urine, and other fluids, employing a dictionary mapping to streamline similar kinds. The resulting pivot table included columns for diverse samples, ranging from 'blood culture tests' to 'urine', 'feces', and 'nasal swabs'. The groups of microbiology tests based on sample type are given in Supplementary List 1. Comorbidities were extracted and processed to identify unique diseases from patient records. New columns were created for each disease, and the counts were updated to represent the number of previous disease episodes in the patient records. Finally, aggregate columns were added for ICD and procedure codes to calculate cumulative sums leading up to an episode. This method enabled capturing each patient's cumulative history of medical conditions and procedures.

**1.3. Model Development and Interpretations:**

The development phase employed tree-based ML algorithms eXtreme Gradient Boosting (XGBoost), Light Gradient Boosting Machine (LightGBM), Category Boosting (CatBoost), and Random Forest (RF) and constructed baseline models using Neural Networks (NN) and Logistic Regression (LR) for comparative analysis. Tree-based ML models were selected primarily for their ability to handle tabular data effectively, owing to their inherent characteristics such as insensitivity to feature scaling and rotational variance and robustness against irrelevant features (refer to Supplementary Methods for model selection rationale). Model performance was assessed using various metrics, including Accuracy, Precision, Recall (Sensitivity), F1-score, Specificity, Area Under the Precision-Recall Curve (AUPRC), and Area Under the Receiver Operating Characteristic (AUROC), with definitions for each metric detailed in Supplementary Methods. For model interpretation, the framework integrated SHapley Additive exPlanations (SHAP) to attribute model outputs to individual features, leveraging a game-theoretic approach to ensure equitable feature importance distribution [23]. SHAP's suitability for tree-based models was enhanced by its efficient computation of exact SHAP values using the TreeExplainer algorithm [37], significantly reducing computational complexity by exploiting the structural properties of decision trees [38].

**1.4. ML Models:**

1.4.1. LightGBM Model: The LightGBM model, a gradient boosting framework, is utilized with the scale_pos_weight parameter adjusted to address class imbalances. This model was selected for its efficiency and speed in handling large-scale data. Its gradient-boosting framework can process the extensive dataset with remarkable accuracy and minimal computational resources. LightGBM's use of histogram-based decision trees allows for faster training speed and lower memory usage, making it highly suitable for the complex task of BSI prediction.

LightGBM builds the model in a stage-wise fashion and generalizes it by optimizing a loss function:

$$\hat{y}(x)= \sum_{k=1}^{K} f_{k}(x)$$

Where $f_{k}$ are the individual decision trees and$k$ is the number of boosting stages (trees).

1.4.2. CatBoost Model: CatBoost is another gradient-boosting model that is particularly effective in processing categorical features. Tuned for binary classification, it also handles class imbalance, a critical aspect in predicting BSIs.

CatBoost builds the model in a stage-wise fashion and generalizes them by optimizing a loss function:

$$\hat{y}(x)= \sum_{k=1}^{K} f_{k}(x)$$

Where $f_{k}$ are the individual decision trees and$k$ is the number of boosting stages (trees).

1.4.3. XGBoost Model: The XGBoost (Extreme Gradient Boosting) model, stands out for its advanced regularization features is employed with a focus on performance and speed. It uses the scale_pos_weight parameter for class imbalance and a logloss evaluation metric, making it suitable for binary classification.

XGBoost builds the model in a stage-wise fashion and generalize them by optimizing a loss function:

$$\hat{y}(x)= \sum_{k=1}^{K} f_{k}(x)$$

Where $f_{k}$ are the individual decision trees and$k$ is the number of boosting stages (trees).

1.4.4. ANN Model: A simple Artificial Neural Network (ANN) with three dense layers (128, 64, and 1 unit) is used, featuring ReLU activation for the first two layers and sigmoid activation for the output layer. This structure allows for modeling complex non-linear relationships in the data. The model is compiled using the Adam optimizer and binary cross-entropy loss. The ANN defined by the following equations, layer-wise:

$$z^{[l+1]}= W^{[l]} a^{[l]}+ b^{[l]}$$

$$a^{[l+1]}= g^{[l]} (z^{\left[ l+1 \right]})$$

Where $W^{[l]}$ and $b^{[l]}$are the weights and biases at layer $l$, $a^{[l]}$ is the activation from the previous layer, $g^{[l]}$

is the activation function, and $z^{\left[ l+1 \right]}$ is the linear combination input to the activation function at layer $l+1$.

1.4.5. RF Model: The Random Forest (RF) classifier with 100 estimators is used for its robustness and effectiveness in handling high-dimensional data, making it suitable for complex medical datasets. A Random Forest aggregates the predictions of multiple decision trees, typically constructed with some form of randomness and then averaged to improve the predictive accuracy and control over-fitting.

The prediction for a new sample $x$ is:

$$\hat{y}= \frac{1}{N} \sum_{i=1}^{N} t_{i}(x)$$

Where $t_{i}$ are the individual decision trees and$N$ is the number of trees in the forest.

1.4.6. LR Model: A Logistic Regression (LR) model is employed for binary classification, known for its simplicity and interpretability, which is crucial in medical settings where understanding model decisions is essential. The logistic function can be represented mathematically as:

$$P\left( y=1 | x \right)= \sigma(W^{T}x+b)$$

Where $\sigma\left( z \right)= \frac{1}{1+e^{-z}}$ is the logistic (sigmoid) function, $W$ is the weight vector, $x$ is the input feature vector, and b is the bias.

**1.5. Performance Metrics:**

1.5.1. Accuracy**:**

This is the ratio of correctly predicted observations (true positives and negatives) to the total observations. It’s a general indicator of a model’s performance.

$$Accuracy=\frac{TP+TN}{TP+FP+FN+TN}$$

Where TP = True Positives, TN = True Negatives, FP = False Positives, FN = False Negatives.

1.5.2. Precision:

Also called Positive Predictive Value, it is the ratio of correctly predicted positive observations to the total predicted positive observations. It shows the model’s ability to return relevant results.

$$Precision= \frac{TP}{TP+FP}$$

1.5.3. Recall (Sensitivity or True Positive Rate):

This is the ratio of correctly predicted positive observations to all actual positives. It measures the model's capability to find all relevant cases.

​

$$Recall= \frac{TP}{TP+FN}$$

1.5.4. F1-score:

The F1-score is the harmonic mean of precision and recall, providing a balance between the two metrics. It’s especially useful when the class distribution is uneven.

​

$$F1-score=2 \times\frac{Precision \times Recall}{Precision+Recall}$$

1.5.5. Specificity (True Negative Rate):

This is the ratio of correctly predicted negative observations to all actual negatives. It measures the model's ability to identify negative results.

$$Specificity= \frac{TN}{TN+FP}$$

1.5.6. Area Under the Precision-Recall (PR) Curve (AUPRC):

This metric summarizes the trade-off between the true positive rate (recall) and the positive predictive value (precision) for a predictive model using different probability thresholds. The AUPRC is particularly insightful in the case of imbalanced datasets. The PR curve plots precision (y-axis) and recall (x-axis) for different threshold values, and AUPRC is the area under this curve.

1.5.7. Area Under the Receiver Operating Characteristic (AUROC):

This is used to evaluate the performance of a binary classification system by plotting the true positive rate (recall) against the false positive rate (1 - specificity) at various threshold settings. The AUROC is the area under the ROC curve, which ranges from 0 to 1. A model that predicts perfectly has an AUROC of 1, while a model that predicts randomly has an AUROC of 0.5. The ROC curve plots sensitivity (y-axis) versus 1-specificity (x-axis) for different threshold values, and AUROC is the area under this curve.

**1.6. Training Procedure**

The training of the ML models followed a structured and systematic process to ensure robustness and generalizability. The procedure incorporated several key steps.

1.6.1 Data Splitting:

The initial step involved splitting the dataset into training and testing subsets to validate the model’s performance. We used the **train_test_split** method from the **sklearn.model_selection** library, setting aside 20% of the data for testing. This split was performed without shuffling to preserve the temporal nature of the data. Further, the training data was divided into a smaller training set and a validation set, with 20% of the training data allocated for validation.

1.6.2. Data Scaling:

Given the sequential and tabular nature of the data, the necessary transformation steps were employed to prepare it for the corresponding learning algorithms. The data was reshaped and scaled using the StandardScaler from sklearn.preprocessing. This normalization step is crucial for models sensitive to input features' scale. After scaling, the data was reshaped to its original form, ensuring compatibility with the machine learning models.

## **1.7. Prediction modeling**

For each predicted adverse outcome. We constructed a dataset *X^∗^* from the event logs using the feature engineering steps. A positive label indicated that patients had adverse outcome at the index episode, which is denoted as *X^+^ ∈ X^∗^* . Negative labels indicated that the patient did not have a adverse outcome at the index episode, which is denoted as *X^−^∈ X^∗^.* During the training phase, and validation phase, we used the data *X^∗^* that contains both *X^+^* and *X*^−^ to train our model Z. For testing, we applied the trained models to predict the patient’s risk of adverse outcomes.

**1.8. Neural Network Parameters**: For the Neural Network (NN) model, we utilized a simple feedforward architecture with three dense layers. The parameters for the NN were selected through a combination of empirical testing and hyperparameter tuning to achieve optimal performance. Below are the details of the parameters used and the process for their selection:

- **Architecture**:
  - **Input Layer**: The input layer size was determined by the number of features in the dataset.
  - **Hidden Layers**:
    - **First Hidden Layer**: 128 units with ReLU (Rectified Linear Unit) activation function.
    - **Second Hidden Layer**: 64 units with ReLU activation function.
  - **Output Layer**: A single unit with a Sigmoid activation function for binary classification.
- **Hyperparameters**:
  - **Optimizer**: Adam optimizer was selected for its efficiency and adaptive learning rate capabilities.
  - **Learning Rate**: Initially set to 0.001. This value was chosen based on standard practice and further fine-tuned during training.
  - **Batch Size**: 32. This parameter was chosen to balance between training speed and convergence stability.
  - **Epochs**: 100. Early stopping was implemented to monitor validation loss and prevent overfitting. Training was halted if validation loss did not improve for ten consecutive epochs.
  - **Loss Function**: Binary cross-entropy, suitable for binary classification tasks.
- **Regularization and Dropout**:
  - **Dropout**: Dropout layers were added after each hidden layer to prevent overfitting, with a dropout rate of 0.5.
  - **L2 Regularization**: Applied to the weights of the hidden layers to further reduce the risk of overfitting.
- **Initialization**:
  - **Weights Initialization**: He normal initialization was used to initialize the weights, which is well-suited for layers with ReLU activation.
- **Activation Functions**:
  - **ReLU Activation**: Chosen for hidden layers due to its effectiveness in handling vanishing gradient problems.
  - **Sigmoid Activation**: Used in the output layer to produce probabilities for the binary classification task.

## **1.9. Class Imbalance Handling**

The primary method used to handle class imbalance was the adjustment of the **scale_pos_weight** parameter in gradient boosting models and using oversampling and undersampling techniques.

- **Gradient Boosting Models**:
  - For the **XGBoost** and **LightGBM** models, the **scale_pos_weight** parameter was tuned to balance the positive and negative classes. This parameter adjusts the weight of positive class samples to counter the imbalance.
  - In **XGBoost**, **scale_pos_weight** was set based on the ratio of negative to positive samples:

scale_pos_weight = number_of_negative_samples / number_of_positive_samples

- - Similarly, in **LightGBM**, the **is_unbalance** parameter was set to **true**, enabling automatic adjustment of class weights based on the imbalance ratio.
- **CatBoost**:
  - The **CatBoost** model inherently handles class imbalance using its internal mechanisms. We set the **class_weights** parameter to explicitly define the weights for each class based on their frequencies.
- **Random Forest**:
  - For the **Random Forest** model, we used the **class_weight** parameter set to **balanced**, which adjusts the weights inversely proportional to the class frequencies in the input data.

**2. Supplementary Results**

For comprehensive details on the ICD-10 codes used in this study, please refer to the Norwegian Directorate of eHealth's ICD-10 code directory: [https://finnkode.ehelse.no/#icd10/0/0/0/-1](file:////Users/rajeevbopche/Library/Containers/com.microsoft.Word/Data/Library/Preferences/AutoRecovery/For%20comprehensive%20details%20on%20the%20ICD-10%20codes%20used%20in%20this%20study,%20please%20refer%20to%20the%20Norwegian%20Directorate%20of%20eHealth's%20ICD-10%20code%20directory:%20https:/finnkode.ehelse.no#icd10/0/0/0/-1    Additional information on clinical procedures relevant to our methodology can be found at the Norwegian Clinical Procedure Code Directory (NKPK): https://www.ehelse.no/kodeverk-og-terminologi/Norsk-klinisk-prosedyrekodeverk-(NKPK))

Additional information on clinical procedures relevant to our methodology can be found at the Norwegian Clinical Procedure Code Directory (NKPK): [https://www.ehelse.no/kodeverk-og-terminologi/Norsk-klinisk-prosedyrekodeverk-(NKPK)](file:////Users/rajeevbopche/Library/Containers/com.microsoft.Word/Data/Library/Preferences/AutoRecovery/For%20comprehensive%20details%20on%20the%20ICD-10%20codes%20used%20in%20this%20study,%20please%20refer%20to%20the%20Norwegian%20Directorate%20of%20eHealth's%20ICD-10%20code%20directory:%20https:/finnkode.ehelse.no#icd10/0/0/0/-1    Additional information on clinical procedures relevant to our methodology can be found at the Norwegian Clinical Procedure Code Directory (NKPK): https://www.ehelse.no/kodeverk-og-terminologi/Norsk-klinisk-prosedyrekodeverk-(NKPK))

**Supplementary Table 1.** **Mean values of the predictors (Top 10 most significant) for the four prediction tasks.**

| **Predictors** | | | | |
| --- | --- | --- | --- | --- |
| **Task 1:** **30 day mortality prediction at discharge** | | **Unit** | **Mean (Class 0)** | **Mean (Class 1)** |
| Urgency_code (Emergency code) | | Category | 0.68 * | 0.63 * |
| ICD_J_aggregate (Diseases of the respiratory system) | | Counts | 2.10 | 4.13 |
| ICD_I_aggregate (Diseases of the circulatory system) | | Counts | 5.21 | 8.98 |
| CRP (Inflammation indicator) | | mg/L | 20.84 | 82.77 |
| ICD_C_aggregate (Cancer) | | Counts | 5.71 | 21.92 |
| Procedure_R_aggregate (Blood with components) | | Counts | 1.20 | 1.80 |
| Care_level_code (Care level code) | | Category | 0.70 * | 0.69 * |
| LOS (Length of stay of the index episode) | | Days | 1.17 | 5.03 |
| Time_to_last (Time the last episode) | | Hours | 4029.56 | 1528.22 |
| Leukocytes (LEUKOCYTTER) | | 10^9^/L | 5.59 | 11.08 |
|  | **Task 2:** **30 day mortality prediction at admission** | | | |
| ICD_C (Cancer related) | | Counts | 0.10 | 0.66 |
| CRP (C-Reactive Protein) | | mg/L | 30.33 | 74.67 |
| Leukocytes (LEUKOCYTTER) | | 10^9^/L | 6.57 | 10.83 |
| ICD_I (Diseases of the circulatory system) | | Counts | 0.20 | 0.74 |
| ICD_J (Diseases of the respiratory system) | | Counts | 0.14 | 0.49 |
| Procedure_R_aggregate (Blood with components) | | Counts | 1.20 | 1.80 |
| ICD_J_aggregate (Diseases of the respiratory system) | | Counts | 2.10 | 4.13 |
| ICD_I_aggregate (Diseases of the circulatory system) | | Counts | 5.21 | 8.98 |
| Age | | Years | 55.44 | 75.13 |
| Total_los (Total cumulative hospital Length of Stay) | | Days | 29.37 | 55.32 |
| **Task 3:** **Readmission prediction at discharge** | |  |  |  |
| Urgency_code | | Category | 0.68 * | 0.49 * |
| Care_level_code | | Category | 0.67 * | 0.50 * |
| Readmission_flag (Flag to denote the current episode being a readmission) | | Flag | 0.10 | 0.27 |
| CRP (C-Reactive Protein) | | mg/L | 24.32 | 52.30 |
| LOS (Length of stay of the index episode) | | Days | 1.53 | 2.15 |
| Age | | Years | 55.24 | 67.17 |
| ICD_C (Cancer related) | | Counts | 0.15 | 0.37 |
| ICD_C_aggregate (Cancer related) | | Counts | 7.42 | 16.12 |
| Leukocytes () | | 10^9^/L | 5.75 | 8.49 |
| Total_los (Total cumulative hospital Length of Stay) | | Days | 30.80 | 42.78 |
| **Task 4:** **PLOS prediction at admission** | |  |  |  |
| ICD_N (Diseases of the urinary and genital organs) | | Counts | 0.08 | 0.31 |
| Urgency_code | | Category | 0.79 * | 0.89 * |
| Care_level_code | | Category | 0.81 * | 1.0 * |
| CRP (C-Reactive Protein) | | mg/L | 28.70 | 72.40 |
| Age | | Years | 56.74 | 67.99 |
| ICD_I (Diseases of the circulatory system) | | Counts | 0.16 | 0.76 |
| ICD_J (Diseases of the respiratory system) | | Counts | 0.09 | 0.57 |
| Leukocytes () | | 10^9^/L | 6.65 | 9.91 |
| ICD_E (Endocrine diseases, nutritional diseases and metabolic disorders) | | Counts | 0.05 | 0.27 |
| ICD_F (Mental disorders and behavioral disorders) | | Counts | 0.03 | 0.14 |

Table 5: Mean values of the predictors (Top 10 most significant) for the four prediction tasks.
Note: * Represents class proportions for the respective categorical variables.

Note: The items listed for each outcome are in order of decreasing significance or predictiveness. This ordering helps highlight the most impactful predictors for each adverse outcome, providing valuable insights for clinical decision-making.

**TRIPOD checklist**

| **Section/Topic** | **It** | **Checklist Item** | **Page** |
| --- | --- | --- | --- |
| **Title and abstract** | | | |
| Title | 1 | Identify the study as developing and/or validating a multivariable prediction model, the target population, and the outcome to be predicted. | 1 |
| Abstract | 2 | Provide a summary of objectives, study design, setting, participants, sample size, predictors, outcome, statistical analysis, results, and conclusions. | 1 |
| **Introduction** | | | |
| Background and objectives | 3a | Explain the medical context (including whether diagnostic or prognostic) and rationale for developing or validating the multivariable prediction model, including references to existing models. | 2 |
|  | 3b | Specify the objectives, including whether the study describes the development or validation of the model or both. | 5 |
| **Methods** | | | |
| Source of data | 4a | Describe the study design or source of data (e.g., randomized trial, cohort, or registry data), separately for the development and validation data sets, if applicable. | 6 |
|  | 4b | Specify the key study dates, including start of accrual; end of accrual; and, if applicable, end of follow-up. | 6 |
| Participants | 5a | Specify key elements of the study setting (e.g., primary care, secondary care, general population) including number and location of centres. | 6 |
|  | 5b | Describe eligibility criteria for participants. | 6 |
|  | 5c | Give details of treatments received, if relevant. |  |
| Outcome | 6a | Clearly define the outcome that is predicted by the prediction model, including how and when assessed. | 8 |
|  | 6b | Report any actions to blind assessment of the outcome to be predicted. | 8 |
| Predictors | 7a | Clearly define all predictors used in developing or validating the multivariable prediction model, including how and when they were measured. | 8 |
|  | 7b | Report any actions to blind assessment of predictors for the outcome and other predictors. | 8 |
| Sample size | 8 | Explain how the study size was arrived at. | 8 |
| Missing data | 9 | Describe how missing data were handled (e.g., complete-case analysis, single imputation, multiple imputation) with details of any imputation method. | 7 |
| Statistical analysis methods | 10a | Describe how predictors were handled in the analyses. | 8 |
|  | 10b | Specify type of model, all model-building procedures (including any predictor selection), and method for internal validation. | 7 & 8 |
|  | 10d | Specify all measures used to assess model performance and, if relevant, to compare multiple models. | 7 & 8 |
| Risk groups | 11 | Provide details on how risk groups were created, if done. | 8 |
| **Results** | | | |
| Participants | 13a | Describe the flow of participants through the study, including the number of participants with and without the outcome and, if applicable, a summary of the follow-up time. A diagram may be helpful. | 8 |
|  | 13b | Describe the characteristics of the participants (basic demographics, clinical features, available predictors), including the number of participants with missing data for predictors and outcome. | 9 |
| Model development | 14a | Specify the number of participants and outcome events in each analysis. | 8 |
|  | 14b | If done, report the unadjusted association between each candidate predictor and outcome. | 11 & 12 |
| Model specification | 15a | Present the full prediction model to allow predictions for individuals (i.e., all regression coefficients, and model intercept or baseline survival at a given time point). | 11 & 12 |
|  | 15b | Explain how to the use the prediction model. | 7 |
| Model performance | 16 | Report performance measures (with CIs) for the prediction model. | 10 |
| **Discussion** | | | |
| Limitations | 18 | Discuss any limitations of the study (such as nonrepresentative sample, few events per predictor, missing data). | 14 |
| Interpretation | 19b | Give an overall interpretation of the results, considering objectives, limitations, and results from similar studies, and other relevant evidence. | 13 |
| Implications | 20 | Discuss the potential clinical use of the model and implications for future research. | 14 & 15 |
| **Other information** | | | |
| Supplementary information | 21 | Provide information about the availability of supplementary resources, such as study protocol, Web calculator, and data sets. | 16 |
| Funding | 22 | Give the source of funding and the role of the funders for the present study. | 16 |
